# *N*-Arylsulfonamidocalix[4]arenes with narrow pH-responsive binding near neutral pH

**DOI:** 10.1039/d5sc07965a

**Published:** 2025-12-15

**Authors:** Carlos Alarcon-Miranda, Isis A. Middleton, Olivia Rusli, Nicolas Caceres-Herrera, Mohan Bhadbhade, Nicole J. Rijs, Pall Thordarson, Marcelo J. Kogan, Claudio Saitz

**Affiliations:** a Facultad de Ciencias Químicas y Farmacéuticas Universidad de Chile Santiago 8380494 Chile mkogan@ciq.uchile.cl clsaitz@ciq.uchile.cl; b School of Chemistry, The University of New South Wales Sydney NSW 2052 Australia p.thordarson@unsw.edu.au; c UNSW RNA Institute, The University of New South Wales Sydney NSW 2052 Australia; d Mark Wainwright Analytical Centre, The University of New South Wales Sydney NSW 2052 Australia; e Advanced Center for Chronic Diseases (ACCDiS), Universidad de Chile Santiago 8380494 Chile

## Abstract

We report a series of *N*-arylsulfonamidocalix[4]arene hosts with tunable acidity and conformational flexibility designed to explore narrow pH-responsive binding. Using two cationic dyes as model guests, we performed a systematic analysis across pH 5.8–10.2 in a 1 : 1 (v/v) water : acetonitrile mixture (*χ*_H_2_O_ 0.74). Fluorescence experiments confirmed pH- and conformation-dependent affinity, with host Tf-SA4 (bearing the most acidic sulfonamide groups in the *cone* conformer) inducing a sharp fluorescence enhancement within a narrow range of pH_*app*_ 7.8 and 8.2 (ΔpH = 0.4). Detailed analysis revealed a 1 : 2 complex with coupled charge and geometry changes in the host that are pH-dependent and induce a steep increase in binding stability, consistent with a proposed “binding switch” over the narrow ΔpH = 0.4. This study introduces a *proof-of-concept* for a synthetic host with narrow pH responsiveness near neutral pH, offering a foundation for further research into *N*-arylsulfonamidocalixarenes for potential biomedical applications.

## Abstract 2

Ionization-driven conformational changes in *N*-arylsulfonamidocalix[4]arenes enable a narrow-range, pH-controlled “binding switch” for cationic dyes near neutral pH.

## Graphical abstract

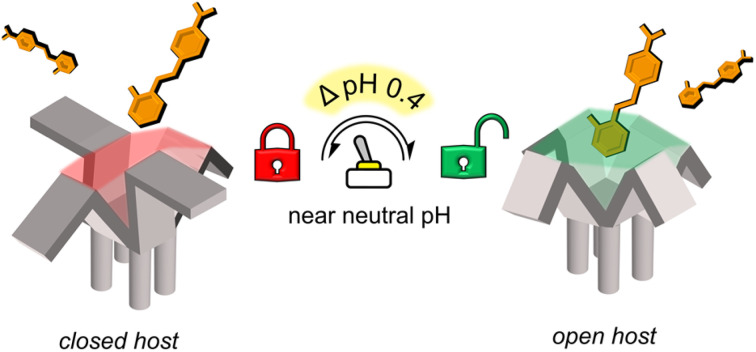

## Introduction

The extracellular pH of healthy tissues (including blood) ranges around neutral pH, from 7.0 to 7.6, while tumor tissues show a slightly acidic difference of ΔpH 0.3 to 0.7.^1^ This difference occurs in solid tumors as they exhibit altered metabolism, leading to increased lactic acid production and an acidic tumor microenvironment.^2^ In that sense, pH-controlled release in drug delivery systems is a current research approach for targeted cancer treatment.^3^ However, these largely rely on polymers or aggregates, using acid-labile linkers^4^ or charge reversal^5^ to form supramolecular structures that disassemble in response to large pH changes, typically within a broad experimental range (ΔpH 1–3.4)^3^ from pH 7.4. In that context, host–guest chemistry has been less explored as a platform for pH-controlled release. For these systems to be effective in *in vivo* pH gradients, an acidic host must ionize within ΔpH 0.3 to 0.7 near neutral pH, enabling differential binding toward cationic guests between healthy and tumor microenvironments. Examples of acidic hosts include sugammadex,^6^ a γ-cyclodextrin containing carboxylic acids; WP6;^7^ and Pillar[6]MaxQ,^8^ which are pillararenes featuring carboxylic and sulfonic acids respectively. These hosts form strong complexes with cationic guests but their application in pH-controlled release is limited, because their ionizable functional groups are mostly deprotonated above pH 5.0 due to their p*K*_a_ values.

**Scheme 1:**
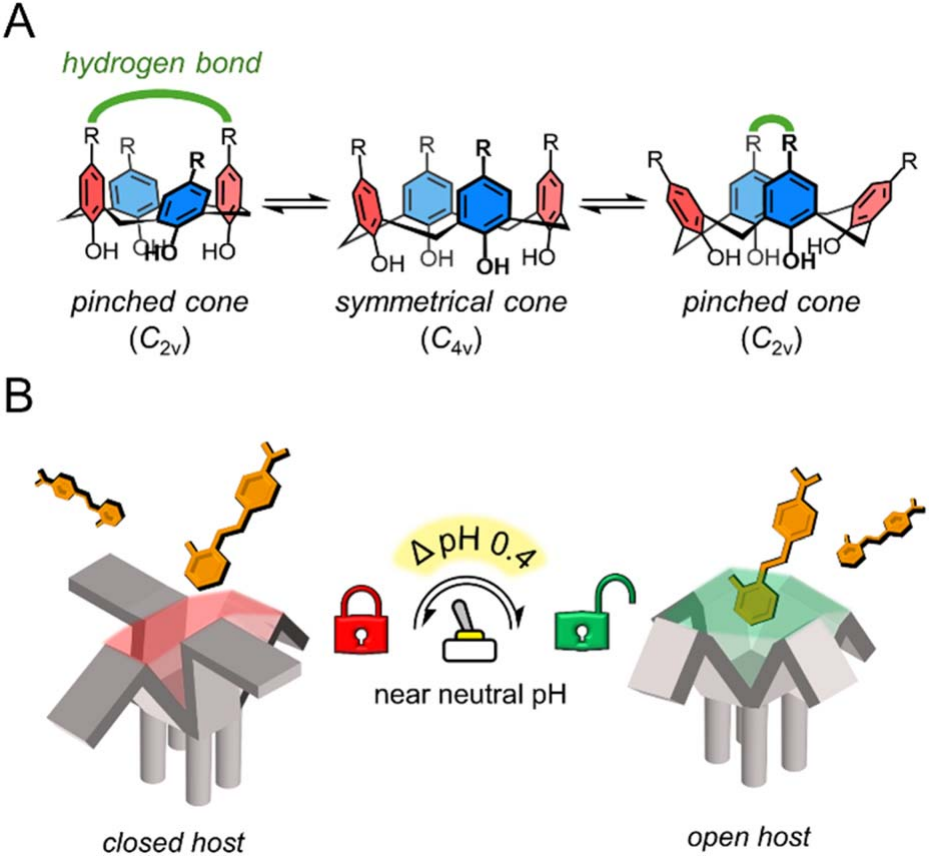
(A) Conformational dynamics of calix[4]arenes in the *cone* conformation. Groups with hydrogen bonding capabilities (green) can stabilize the pinched cone conformation (*C*_2v_). (B) pH-triggered “binding switch” model proposed based on the findings of this work.

*N*-arylbenzenesulfonamides (Ar–SO_2_NH–Ar) are of particular interest in this regard, as they can have different p*K*_a_ values ranging from 5.7 to 10.2,^9,10^ by simply varying the substituent electronic nature of the *N*-aryl moiety. These compounds don't require *N*-heterocycles to modulate acidity like classical sulfonamides (commonly found in antibiotics and some drug delivery systems), which are known to cause severe allergic reactions in susceptible individuals.^11^ Additionally, calix[*n*]arenes are a class of macrocyclic structures (made of *n* phenol subunits connected by methylene bridges at-*ortho* positions) used previously in biomedical settings^12,13^ with excellent synthetic versatility compared with other hosts:^14^ they can undergo transformations such as electrophilic aromatic substitutions while the phenol moieties can be alkylated or esterified. Calix[4]arenes bearing –SO_2_NRR' substituents at the -*para* positions have been previously reported by several research groups.^15–18^ However, calix[4]arenes functionalized with *N*-arylsulfonamide groups have only been described twice for anion recognition in their neutral form, either in acetonitrile^19^ or in heterophasic extraction,^20^ with no subsequent studies reported to date. These calix[4]arenes can bear tunable ionizable groups with p*K*_a_ values near neutral pH. Additionally, groups with hydrogen bonding capabilities in the upper rim can alter the host geometry by stabilizing an pinched *cone* conformer (Scheme 1A), controlling the cavity's availability.^21,22^ The combination of these features has the potential of modulating the binding of this class of hosts toward cationic guests in a pH-dependent manner, extending our prior work on cation recognition with calix[4]arenes.^23,24^

In this study, we address the gap in pH-responsive host–guest chemistry by designing *N*-arylsulfonamidocalix[4]arene hosts with tunable ionization properties in a near-aqueous environment (water mole fraction (*χ*_H_2_O_) = 0.74, *χ*_CH_3_CN_ = 0.26). Through fluorescence assays, NMR, statistical analysis, nESI-MS, and X-ray crystallography, we show that these hosts follow a pH-triggered “binding switch” model, controlled by changes in the ionization state and cavity availability, with a significant increase in stability within a narrow ΔpH = 0.4 between pH_*app*_ 7.8 and 8.2 (Scheme 1B). This work highlights the potential of macrocycles to undergo both charge and geometrical changes in response to narrow pH variations, particularly near pH neutrality, offering a new platform for the design of highly responsive supramolecular systems with potential biomedical applications.

## Results and discussion

### *N*-Arylsulfonamidocalix[4]arenes have tunable pH-dependent and conformation-dependent binding with cationic dyes

**Fig. 1:**
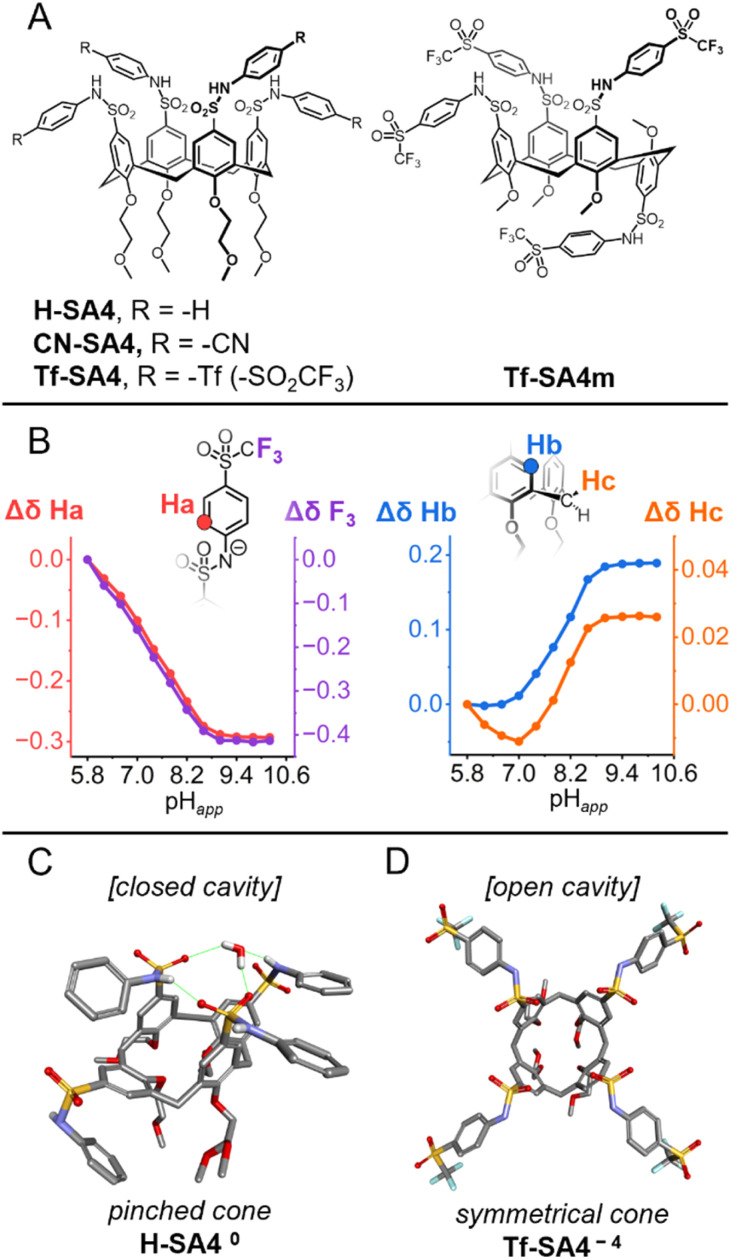
(A) *N*-Arylsulfonamidocalix[4]arenes synthesized in this study. (B) The ionization behavior of Tf-SA4 (0.1 mM). Δ*δ* values of selected protons (600 MHz, 298 K) and the fluorines (^19^F NMR experiments, 565 MHz, 298 K), measured from pH_*app*_ 5.8 to 10.2, using a mixture of 1 : 1 (v/v) H_2_O : CD_3_CN (*χ*_H_2_O_ 0.74), with 10 mM buffer (phosphate buffer for pH_*app*_ 5.8 to 7.8 and borate buffer for pH_*app*_ 8.2 to 10.2) (C and D). The conformational differences between ionization states of H-SA4 (C) and Tf-SA4 (D) based on the X-ray resolved crystal structures. (C) H-SA4^0^ in the protonated state (top view), showing the hydrogen bonding network (shown in green) that alters its shape to the *pinched cone* conformation (non-polar hydrogens omitted for clarity). (D) The tetrasodium salt of Tf-SA4^−4^ (top view), displaying the *symmetrical cone* conformation (solvent molecules, hydrogens and sodium ions omitted for clarity).

Achieving differential guest binding near neutral pH requires host systems with finely tuned ionization behavior. To this end, we synthesized a series of *N*-arylsulfonamidocalix[4]arenes *via para*-sulfonamide functionalization of tetraalkylated calix[4]arene in the *cone* conformation, spanning a systematic increase in sulfonamide acidity *via N*-aryl substitution (SI, Scheme S1). As shown in Fig. 1A, H-SA4 (S2.3) features unsubstituted *N*-aryl groups, CN-SA4 (S2.4) contains a cyano substituent at the 4-position, and Tf-SA4 (S2.5) carries triflyl (–SO_2_CF_3_) groups, a strong electron-withdrawing substituent to significantly lower the sulfonamides' p*K*_a_. A conformational analogue, Tf-SA4m, was also prepared and isolated in the *partial cone* form (S2.8) to evaluate the role of geometry in guest recognition. In the design of the *cone*-shaped hosts, we aimed to take advantage of the unique conformational flexibility of calix[4]arenes, which can exist dynamically in solution as two different “*cone* symmetries” (Scheme 1A):^21^ In the *symmetrical cone* (*C*_4v_ symmetry), the aromatic subunits form an open cavity, but they move quickly to the *pinched cone* (*C*_2v_ symmetry), in which two opposite subunits move closer to each other, narrowing the cavity.

The pinched conformer interconverts into another *pinched cone* through the *symmetrical cone*. This equilibrium can be shifted to the *C*_2v_ symmetry by stabilizing the pinching with intramolecular hydrogen bonds between distal substituents at the upper rim.^22^

To validate the design of Tf-SA4, its ionization and conformational behavior were studied across a pH range of 5.8–10.2 by NMR. Experiments were conducted in a 1 : 1 (v/v) mixture of water and deuterated acetonitrile to ensure sufficient solubility of the host across the studied pH range. Although this mixture contains 50% water by volume, the molar fraction of water (*χ*_H_2_O_) is 0.74, resulting in a solvent environment in which water-like properties such as polarity and hydrogen bonding networks are largely preserved.^25^ All reported pH values correspond to the values measured in the aqueous part of the mixture before mixing, termed *apparent* pH (pH_*app*_, see SI, S1.1 for details). However, due to the reduced dielectric constant relative to pure water, the operational pH scale in this medium is slightly shifted to higher values.^26^

^1^H and ^19^F NMR experiments showed continuous upfield shifts from pH_*app*_ 5.8 in the sulfonamide-linked nuclei (Ha, F_3_, Fig. 1B), indicating a gradual deprotonation of the *N*-arylsulfonamide groups from pH_*app*_ 5.8 to 9.0, with no evidence of distinct deprotonation states (Fig. 1B). This confirmed that the triflyl group in Tf-SA4 provides sufficient acidity to ionize the sulfonamides at near neutral pH as desired.

In parallel, subtle changes were observed in the chemical shifts of the calix[4]arene core protons (Hb and Hc), suggesting an ionization-dependent reorganization of the *cone* geometry (Fig. 1B). The interconversion between the *C*_4v_ and *C*_2v_ symmetries occurs in fast exchange on the NMR time scale at 25 °C, making them difficult to distinguish under standard conditions.^22^ Nevertheless, conformational insights can be extracted from this data. Notably, Hc initially shifts upfield slightly from pH_*app*_ 5.8 to 7.0. Since sulfonamides deprotonation is detected at pH_*app*_ ∼6.2, this trend likely reflects a stronger averaged shielding due to the stabilization of the *pinched cone* geometry by charge-assisted intramolecular hydrogen bonding between a protonated sulfonamide and a negatively charged distal sulfonamide. Above pH_*app*_ 7.0, Tf-SA4 gets more ionized and both Hc and Hb move downfield, consistent with a population shift toward a more open, *symmetrical cone* geometry. This reorganization increases the distance between opposing aromatic rings, reducing their mutual shielding.

To demonstrate the effect of charge in *N*-arylsulfonamidocalix[4]arene geometry, we obtained crystal structures of H-SA4^0^ (protonated) and Tf-SA4^−4^ (fully deprotonated). H-SA4^0^ adopts a *pinched cone* conformation (distances between distal subunits: 5.0 Å, 7.6 Å), stabilized by intramolecular hydrogen bonds between distal sulfonamide groups (N–H⋯O=S, 2.1 Å), including one bridging water molecule (Fig. 1C). In contrast, fully deprotonated Tf-SA^−4^ (tetrasodium salt) adopts a *symmetrical cone* conformation (distances between distal subunits: 6.2 Å, 6.8 Å), consistent with coulombic repulsion between the negatively charged sulfonamide groups (Fig. 1D). Together, crystallographic and NMR data show that this class of hosts restricts cavity access in the neutral state and opens it upon full ionization, supporting the geometric rationale behind the molecular design.

**Fig. 2:**
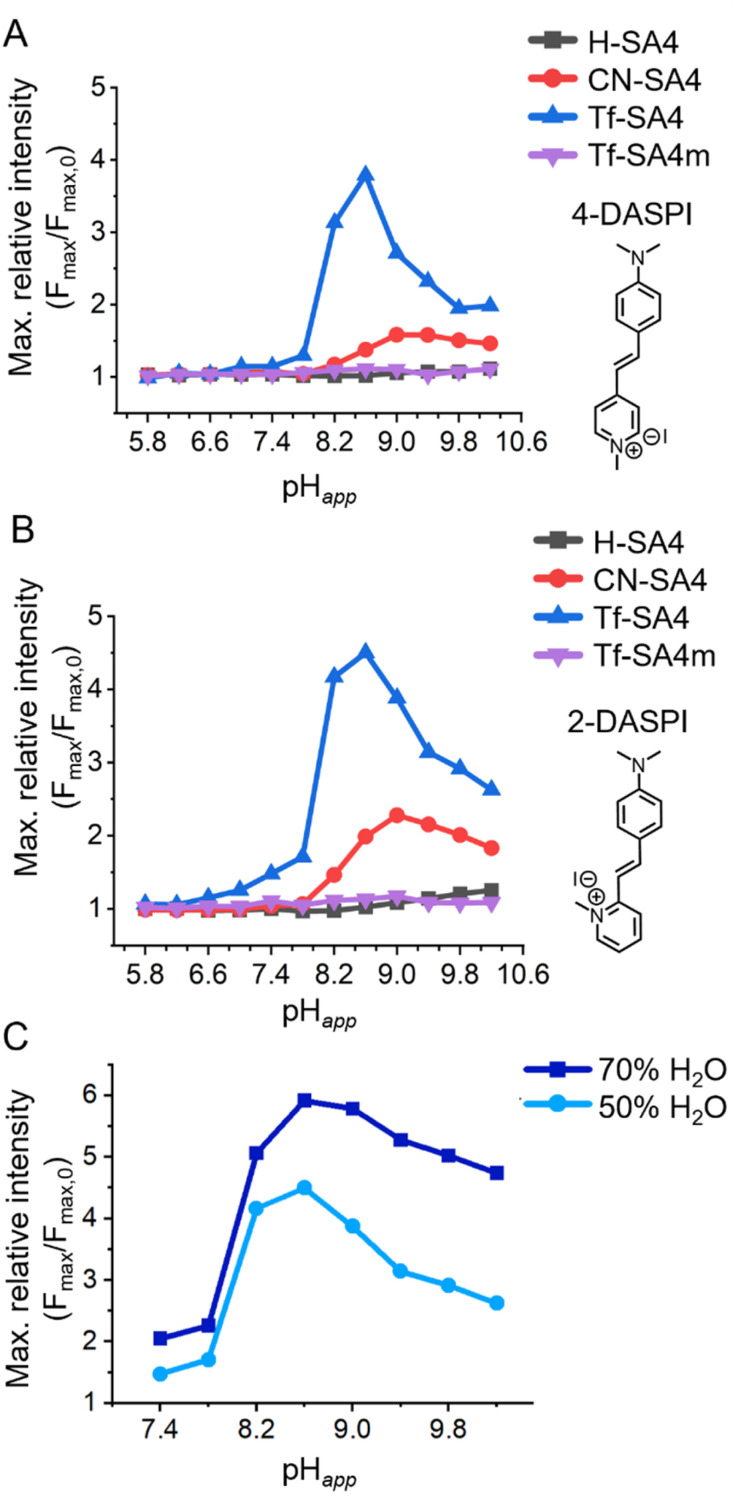
The sharp binding response between pH_*app*_ 7.8 and 8.2 for Tf-SA4. Changes in maximum relative fluorescence intensity of (A) 4-DASPI (2 µM, *λ*_ex_ 460 nm) and (B) 2-DASPI (2 µM, *λ*_ex_ 450 nm) in absence and presence of 50 eq. (0.1 mM) of each host at 12 different pH_*app*_ points in the range of 5.8 to 10.2. Solutions were prepared using a 1 : 1 (v/v) H_2_O : CH_3_CN mixture (*χ*_H_2_O_ 0.74), with 10 mM buffer (sodium phosphate buffer for pH_*app*_ range 5.8–7.8 and sodium borate buffer for 8.2–10.2). (C) Effect of the water content on the sharp fluorescence enhancement of Tf-SA4 (0.1 mM) over 2-DASPI (2 µM, *λ*_ex_ 450 nm) in 1 : 1 (v/v) H_2_O : CH_3_CN (light blue line, data from Fig. 2B, *χ*_H_2_O_ 0.74) and 7 : 3 (v/v) H_2_O : CH_3_CN (blue line, *χ*_H_2_O_ 0.87), from pH_*app*_ 7.4–10.2.

The binding capabilities of these hosts at different pH values were studied using a semi-quantitative fluorescence assay employing two dyes used as model cationic guests, 2-DASPI and 4-DASPI (Fig. 2). These dyes were selected for their simple structure, bearing a *N*-methyl pyridinium core that combines positive charge and hydrophobic character. The pyridinium moiety, with its appropriate size and differing substitution patterns in 2-DASPI and 4-DASPI, provides suitable fluorescent probes that allow the hosts to engage in different potential binding modes.

The results of this screening assay for each dye are presented in Fig. 2A and B. H-SA4 is the least acidic host and shows practically no response with either dye in the studied pH_*app*_ range. CN-SA4 is the second most acidic host and induces a weak fluorescence enhancement in both dyes that peaks at pH_*app*_ ≈9.0. Tf-SA4, the most acidic host of the series, induces a sharper and stronger fluorescence response in both dyes also, between pH_*app*_ 7.8 and 8.2 and with a maximum relative intensity at around pH_*app*_ 8.6 of ≈ 4.5 for 2-DASPI and ≈ 4.0 for 4-DASPI. As a control, the *partial cone* analogue Tf-SA4m showed no fluorescence change with either dye, reinforcing the importance of the *cone* conformation in enabling binding-induced response.

To investigate whether the observed pH-dependent binding is linked to the cosolvent system, fluorescence assays with Tf-SA4 and 2-DASPI were performed at both 50% and 70% water by volume (*χ*_H_2_O_ 0.74 and 0.87, respectively) (Fig. 2C). The sharp enhancement in dye fluorescence intensity induced by Tf-SA4 between pH_*app*_ 7.8–8.2 is preserved and even increased in the more aqueous medium, suggesting that the binding behavior arises from specific ionization and conformational changes in the host that respond to pH changes in an aqueous environment. Modest fluorescence enhancements and hypsochromic shifts (SI, Fig. S26–S29) point to partial inclusion of the dye. The response is consistent with the limited fit between host and guest, as full encapsulation would restrict styryl rotation and further enhance fluorescence.

### Tf-SA4 forms 1 : 2 complexes with 2-DASPI of varying strengths at different pH_*app*_ values

**Fig. 3:**
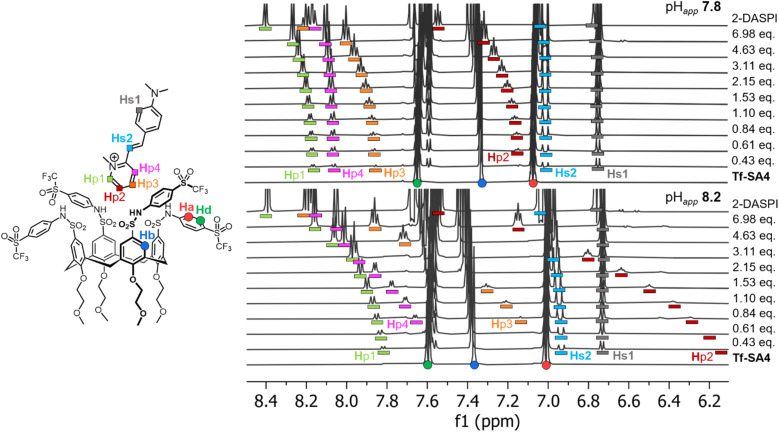
The 2-DASPI guest binds Tf-SA4 through the pyridinium moiety and in a stronger manner at pH_*app*_ 8.2 *vs.* 7.8, based in Δ*δ* magnitudes. Partial ^1^H NMR titrations (600 MHz, 298 K) in 1 : 1 (v/v) H_2_O : CD_3_CN (*χ*_H_2_O_ 0.74), 50 mM buffer (sodium phosphate buffer for pH_*app*_, 7.8 and sodium borate buffer for pH_*app*_ 8.2) of Tf-SA4 with 2-DASPI (guest equivalents added to host (constant concentration, 1 mM)). Host and guest protons are assigned with coloured shapes in their respective structures: Tf-SA4 signals as (●) and 2-DASPI signals as (■). In both spectra, 2-DASPI resonances are displayed with bar below with their assigned color.

^1^H NMR titrations were used to confirm and quantify the binding events of Tf-SA4 with 2-DASPI, at pH_*app*_ 7.4, 7.8, 8.2, and 8.6 (SI, Fig. S33–S36). We attempted to perform these experiments using 70% water but precipitation was observed during the titrations, therefore 50% water was used. The aromatic region of the experiments at pH_*app*_ 7.8 and 8.2 was monitored, where specific protons of 2-DASPI exhibited perturbations in their chemical shift (*δ*), particularly the pyridinium moiety, which moved upfield in a pH-dependent manner (Fig. 3). The Δ*δ* of the pyridinium proton (Hp2) increases substantially (from free guest signals) after only a small increase in the pH_*app*_, shifting 1.48 ppm at pH_*app*_ 8.2 compared to 0.42 ppm at pH_*app*_ 7.8, likely due to stronger shielding from the host cavity. This clearly supports that 2-DASPI interacts through this moiety. Tf-SA4 signals were also monitored for changes in the chemical shift, and the calix[4]arene core protons (Hb and Hc from Fig. 1A) exhibited clear perturbations upon the addition of 2-DASPI and were used for the fitting process (SI, Fig. S37).

The titrations were completed in triplicate at the different pH_*app*_ values, and each dataset was analyzed using the http://supramolecular.org/ (Bindfit)^27^ web tool. All 9 available models were evaluated to determine the complex stoichiometry (SI, Tables S4–S11) but the fitting results of 2 : 1 host–guest models were rejected due to poor fits or non-physical (negative) constants. Results based on 1 : 1, 1 : 2 full, and 1 : 2 non-cooperative models (*K*_1_ = 4·*K*_2_)^28^ were compared using the covariance of fit (cov_fit_)^29^ and Bayesian Information Criterion (BIC)^30^ values (SI, Table S12) to determine which binding model best described the observed data.^31^ At all pH_*app*_ values, both 1 : 2 models gave significantly lower cov_fit_ and BIC than the 1 : 1 model, supporting higher-order complex formation. As the full and non-cooperative 1 : 2 models performed similarly, the simpler non-cooperative model was chosen as the one that best describes this system for its statistical robustness.

The 1 : 2 complex stoichiometry was experimentally corroborated by nanoelectrospray ionisation-mass spectrometry (nESI-MS). nESI is a softer ionisation technique than ESI, which helps preserve the binding modes from solution during the ionisation process.^32,33^ Stoichiometric determination of a host–guest complex is also unambiguous, as MS is very sensitive to *m*/*z* changes.^34^ At pH_*app*_ 8.2, both the 1 : 1 and the 1 : 2 complexes were detected in nESI-MS spectrum, as expected in a stepwise binding process (SI, Fig. S38).

The calculated binding constants for the 1 : 2 Tf-SA4·(2-DASPI)_2_ complex are presented in Table 1. Weak binding is observed at pH_*app*_ 7.4 and 7.8, as reflected by low microscopic binding constants (*K*_m_ = √*β*_12_ = √*K*_1_*K*_2_, allowing straightforward comparison with 1 : 1 host–guest systems).^29^ Between pH_*app*_ 7.8 and 8.2, the *K*_m_ increases 3.9-fold, indicating stronger binding and matching the observed pH range in which the sharp fluorescence response occurs. At pH_*app*_ 8.6, however, the binding constant decreases.

**Table 1:** Macroscopic non-cooperative stepwise 1 : 2 (*K*_1_ and *K*_2_) and microscopic (*K*_m_ = √*β*_12_ = √*K*_1_*K*_2_) binding constants and the corresponding microscopic Gibbs free energies (Δ*G*_m_) between Tf-SA4 and 2-DASPI, as determined by ^1^H NMR titrations. All values shown are averages with *n* = 3 (SD = standard deviation)

pH_*app*_	*K*_1_/M^−1^ (SD)	*K*_2_/M^−1^ (SD)	*K*_m_/M^−1^	Δ*G*_m_/kJ mol^−1^
7.4	264 (38)	66 (10)	130	−12.1
7.8	381 (100)	95 (25)	190	−13.0
8.2	1480 (430)	370 (110)	740	−16.4
8.6	598 (100)	150 (25)	300	−14.1

Notably, the maximum binding detected by NMR occurs at pH_*app*_ 8.2, which is shifted from the fluorescence maximum at pH_app_ 8.6 (Fig. 2). This is likely due to the higher concentrations used in the NMR titration, particularly the buffer (50 mM *vs.* 10 mM), which increases ionic strength. As shown by Gibb and co-workers, charge screening and ion competition can attenuate binding between charged species.^35^ In our system, the reduced dielectric constant and sodium competition likely contribute to the decreased NMR binding constants and fluorescence responses observed at high pH_*app*_ values. In fact, the fluorescence assay in 70% water from Fig. 2C supports this interpretation, as it shows increased fluorescence responses and less pH-related attenuation (compared to 50% water), due to a higher dielectric constant and therefore, less charge screening.

It is known that water : acetonitrile mixtures exhibit microheterogeneity (*i.e.*, acetonitrile pockets in the water bulk) across a wide mole fraction range (*χ*_CH_3_CN_ ≈ 0.1–0.9).^25^ However, that phenomenon only has significant effect on the ionization of organic acids when the acetonitrile content is above 80% by volume^36^ (*χ*_CH_3_CN_ > 0.58, see SI, Fig. S32), a threshold far above the solvent composition employed in our experiments (*χ*_CH_3_CN_ ≈ 0.26 and 0.13 for 50% and 70% water respectively). The sharp binding transition observed over a narrow ΔpH ≈ 0.4 window in both 50% and 70% water/acetonitrile mixtures and the fact that this was observed using two independent techniques (fluorescence and NMR), confirms that the binding behavior of Tf-SA4 is intrinsic to its molecular properties and not related to the cosolvent system. This highlights the robustness and potential applicability of this new pH-responsive binding mechanism in aqueous environments.

### Tf-SA4 follows a pH-triggered “binding switch” model

**Fig. 4:**
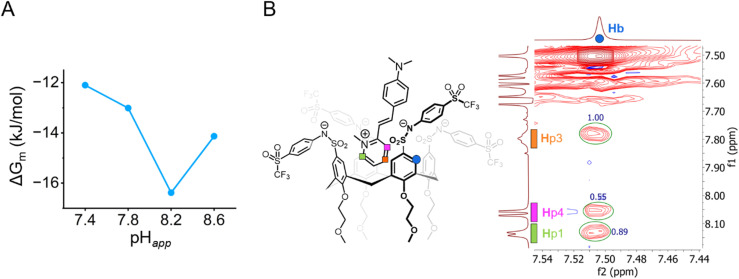
(A) There is a 26% increase (−3.3 kJ mol^−1^ or 1.4 kT) in complex stability from pH_*app*_ 7.8 and 8.2. Measured by the changes in microscopic Gibbs free energy (Δ*G*_m_) from the corresponding microscopic binding constant *K*_m_ (Table 1) for the 1 : 2 non-cooperative stepwise equilibria between Tf-SA4 and 2-DASPI. (B) Partial NOESY NMR (600 MHz, 278 K) spectrum of Tf-SA4 (1 mM) and 2-DASPI (4.5 mM) at pH_*app*_ 8.2 (sodium borate buffer 50 mM) in 1 : 1 (v/v) H_2_O : CD_3_CN (*χ*_H_2_O_ 0.74). 2-DASPI, through the pyridinium moiety, interacts with calix[4]arene cavity (Hb proton). Hp1 and Hp3 show the strongest NOE interaction (based on crosspeaks normalized integration), followed by Hp4, consistent with the proposed binding mode displayed on the left side of the spectrum. No intermolecular NOE contacts were detected in the lower rim of the host.

The increase in binding strength is more readily evaluated in terms of the Gibbs free energy for the microscopic binding events Δ*G*_m_ = –*RT* ln(*K*_m_) as shown in Table 1 at each pH_*app*_ value measured. Between pH_*app*_ 7.8 and 8.2, the Δ*G*_m_ of Tf-SA4 in forming the Tf-SA4·(2-DASPI)_2_ complex increases by −3.3 kJ mol^−1^ (∼1.4 kT) corresponding to a 26% enhancement in binding free energy (Fig. 4A). These calculations, NMR experiments, and X-ray structures point to a pH-triggered “binding switch” model. At pH_*app*_ 7.4–7.8, Tf-SA4 is partially deprotonated and adopts a *pinched cone* stabilized by intramolecular hydrogen bonding, which limits cavity availability and yielding weak 1 : 2 complexes dominated by electrostatic interactions. As pH_*app*_ rises to 8.2, further deprotonation shifts the host toward a *symmetrical cone*, opening the cavity by charge repulsion and enabling stronger binding through both electrostatic and hydrophobic interactions. Crystallographic data from Tf-SA4^−4^ (SI, Fig. S24) show that, in its fully deprotonated state, the host cavity is accessible and capable of accommodating small molecules such as methanol. Following this rationale, nESI-MS at pH_*app*_ 8.2 detects complexes with the host in the 3^−^ and 4^−^ ionization states, indicating that a higher degree of deprotonation correlates with complexation. (SI, Fig. S39). To further support this model, a NOESY NMR experiment was acquired at pH_*app*_ 8.2 (Fig. 4B). The 2D spectrum revealed spatial proximity (<5 Å) between the pyridinium moiety of 2-DASPI and the host cavity, which is consistent with its inclusion. The compilation of these experimental observations reveals that the conformational change triggered by pH modulates access to the binding site.

To put this pH-responsiveness in context and with pH-controlled release applications in mind, there are few examples of acidic hosts for which binding constants to cationic guests have been measured across therapeutically relevant pH ranges. In studies employing WP6 (pillar[6]arene with 12 carboxylic acids) and dicationic guest paraquat, binding constant decreases of about one order of magnitude have been reported^37^ between pH 7.8 and 4.8, equivalent to *a* ≈17% loss in complex stability (SI, Table S13). However, because the pH scale is logarithmic, a ΔpH of 3 in that range actually corresponds to a 1000-fold increase in proton concentration ([H^+^]), far larger than the ΔpH value alone might suggest.

In contrast, in a narrow ΔpH_*app*_ 0.4 window (pH_*app*_ 8.2 to 7.8), Tf-SA4 is capable of reducing its complex stability with a cationic guest by 21% in response to just *a* ≈2.5-fold increase in [H^+^]. This responsiveness is particularly promising given that tumor tissues typically exhibit only a ΔpH 0.3–0.7 relative to normal tissue near neutral pH,^1^ precisely where our system demonstrates maximum responsiveness. This ability to respond to physiologically relevant pH changes, rather than requiring the larger pH shifts needed by existing systems, positions our work as a compelling prototype for advancing pH-controlled release mechanisms through calixarene chemistry. Future research will focus on exploiting their synthetic versatility into developing more sensitive, fully water-soluble hosts or conjugable analogues that can be attached to drug delivery platforms to enable direct biomedical applications.

## Conclusions

In this study, we designed and synthesized a set of *N*-arylsulfonamidocalix[4]arenes with variable acidity to explore their pH-dependent binding capabilities toward cationic guests. Using structural and spectroscopic tools across a range of pH values, we demonstrated that ionization of the sulfonamide groups not only tunes the charge of the hosts, but also controls their geometry. This class of macrocycles shifts from a hydrogen-bonded *pinched cone* in the neutral state to a *symmetrical cone* upon full ionization, effectively controlling access to the cavity. Systematic fluorescence and NMR studies revealed that Tf-SA4 showed a pronounced binding response over a narrow ΔpH_*app*_ window (7.8–8.2) near pH neutrality. This host forms 1 : 2 complexes with 2-DASPI, with measurable variations in stability as a function of pH. The observed behavior arises from a proposed pH-triggered “binding switch”, where synergy between host ionization and geometric reorganization, offers a strategy to finely tune pH-dependent binding through structural design. This work establishes a proof-of-concept for highly pH-responsive host–guest systems using *N*-arylsulfonamidocalix[4]arenes and provides a basis for developing functional macrocycles with physiologically relevant, narrow pH responsiveness, suited for biomedical applications.

## Author contributions

C. A.-M., P. T., M. J. K. and C. S. designed the research. C. A.-M. performed synthesis of calixarenes, fluorescence and NMR experiments. I. A. M. and P. T. guided experiment design and assisted in data interpretation. O. R. and N. J. R. designed and performed nESI-MS experiments. N. C. assisted in the synthesis of Tf-SA4m. M. B. performed X-ray crystallography analyses. C. A.-M and P. T. analysed binding data. Figures were designed by C. A.-M, M. B., I. A. M. and P. T. All the authors discussed the results and commented on the manuscript prior to submission.

## Conflicts of interest

There are no conflicts to declare.

## Supplementary Material

SC-017-D5SC07965A-s001

SC-017-D5SC07965A-s002

## Data Availability

CCDC 2478311 (H-SA0) and 2478312 (Tf-SA4−4) contain the supplementary crystallographic data for this paper.^38*a*,*b*^ The data supporting this article have been included as part of the supplementary information (SI). Supplementary information: synthetic procedures, NMR spectra of all compounds, crystallographic data and NMR titration data. See DOI: https://doi.org/10.1039/d5sc07965a.
