# Supplementary material for: N-Arylsulfonamidocalix[4]arenes with narrow pH-responsive binding near neutral pH
Source: Chem Sci. 2025 Dec 15;17(6):3293–9. doi: 10.1039/d5sc07965a (PMC12720450; doi:10.1039/d5sc07965a)
Supplement: SC-017-D5SC07965A-s001 [file SC-017-D5SC07965A-s001.pdf]

Supplementary Information (SI) for Chemical Science.
This journal is © The Royal Society of Chemistry 2025

Electronic Supporting Information

N-arylsulfonamidocalix[4]arenes with Narrow pH-Responsive Binding near Neutral pH

Carlos Alarcon-Miranda,[a,b,c] Isis A. Middleton,[b,c] Olivia Rusli,[b] Nicolas Caceres,[a] Mohan Bhadbhade,[d] Nicole J. Rijs,[b] Pall Thordarson,*[b,c] Marcelo J. Kogan,*[a] Claudio Saitz*[a].

[a] *Facultad de Ciencias Químicas y Farmacéuticas*
Universidad de Chile
Santiago, 8380494, Chile.
E-mail: mkogan@ciq.uchile.cl, clsaitz@ciq.uchile.cl
[b] *School of Chemistry*
The University of New South Wales
Sydney, NSW 2052, Australia
E-mail: p.thordarson@unsw.edu.au
[c] *UNSW RNA Institute*
The University of New South Wales
Sydney, NSW 2052, Australia
[d] *Mark Wainwright Analytical Centre*
The University of New South Wales
Sydney, NSW 2052, Australia

*

Table of Contents

S1.1 General Experimental ... 3
S1.2 Synthetic overview ... 3

S2. Synthesis and characterizations of H-SA4, CN-SA4, Tf-SA4 and Tf-SA4m ... 5
S2.1 Compound 2a ... 5
S2.2 Compound 3a ... 5
S2.3 H-SA4 ... 6
S2.4 CN-SA4 ... 7
S2.5 Tf-SA4 ... 8
S2.6 Compound 2b ... 9
S2.7 Compound 3b ... 9
S2.8 Tf-SA4m ... 10
S2.9 ^{1}H NMR spectrum of H-SA4 in DMSO-d_6 ... 12
S2.1 ^{13}C{^{1}H} NMR spectrum of H-SA4 in DMSO-d_6 ... 13
S2.2 ^{1}H NMR spectrum of CN-SA4 in DMSO-d_6 ... 14
S2.3 ^{13}C{^{1}H} NMR spectrum of CN-SA4 in DMSO-d_6 ... 15
S2.4 ^{1}H NMR spectrum of Tf-SA4 in CD_3CN ... 16
S2.5 ^{13}C{^{1}H} NMR spectrum of Tf-SA4 in CD_3CN ... 17
S2.6 ^{1}H-^{13}C HSQC NMR spectrum of Tf-SA4 in CD_3CN ... 18
S2.7 DEPT-135 NMR spectrum of Tf-SA4 in CD_3CN ... 19
S2.8 ^{19}F NMR spectrum of Tf-SA4 in CD_3CN ... 20
S2.9 ^{1}H NMR spectrum of Tf-SA4m in CD_3CN ... 21
S2.10 ^{13}C{^{1}H} NMR spectrum of Tf-SA4m in CD_3CN ... 22
S2.11 ^{1}H-^{13}C HSQC NMR spectrum of Tf-SA4m in CD_3CN ... 23
S2.12 DEPT-135 NMR spectrum of Tf-SA4m in CD_3CN ... 24
S2.13 ^{19}F NMR spectrum of Tf-SA4m in CD_3CN ... 25
S2.14 ESI-HRMS spectrum of H-SA4 ... 26
S2.15 ESI-HRMS spectrum of CN-SA4 ... 26
S2.16 ESI-HRMS spectrum of Tf-SA4 ... 27
S2.17 ESI-HRMS spectrum of Tf-SA4m ... 27

S3. ^{1}H and ^{19}F NMR spectrum of Tf-SA4 at different pH$_{app}$ values ... 28

S4. X-ray crystallography data ... 29
S4.1 Single crystal X-ray structure of H-SA4$^{(0)}$ ... 29
S4.2 Single crystal X-ray structure of Tf-SA4$^{(-4)}$ ... 32

S5. 2-DASPI and 4-DASPI emission spectra from the semi-quantitative fluorescence assay 35

S6. NMR titrations and statistical analysis ... 38
S6.1 NMR titration of Tf-SA4 and 2-DASPI at pH$_{app}$ 7.4 ... 39
S6.2 NMR titration of Tf-SA4 and 2-DASPI at pH$_{app}$ 7.8 ... 40
S6.3 NMR titration of Tf-SA4 and 2-DASPI at pH$_{app}$ 8.2 ... 41
S6.4 NMR titration of Tf-SA4 and 2-DASPI at pH$_{app}$ 8.6 ... 42
S6.5 Binding isotherms of Tf-SA4 protons Hb and Hc ... 43
S6.1 Analysis for experiment at pH$_{app}$ 7.4 ... 44
S6.2 Analysis for experiment at pH$_{app}$ 7.8 ... 45
S6.3 Analysis for experiment at pH$_{app}$ 8.2 ... 46
S6.4 Analysis for experiment at pH$_{app}$ 8.6 ... 47
S6.5 Comparative analysis across studied pH$_{app}$ range ... 48

S7. nESI-MS spectra of mixture of Tf-SA4 with 2-DASPI at pH$_{app}$ 8.2 ... 48

S8. NOESY NMR experiment of Tf-SA4·(2-DASPI)$_2$ complex at pH$_{app}$ 8.2 ... 49

S9. Comparative analysis of pH responsiveness of WP6 and Tf-SA4 ... 50

S10. References ... 51

S1.1 General Experimental

Reagents and solvents were purchased from Sigma-Aldrich, Merck, Chem Supply, AkScientific, Enamine, and were used without purification unless stated otherwise. 2-DASPI and 4 DASPI were recrystallized on ethanol/water mixture and dried in vacuum.

Microwave synthesis was performed in a CEM Discover SP reactor, in 10 ml reaction vessels with magnetic stirrers. A constant temperature/variable power mode in closed-vessel conditions were used for the reactions.

NMR spectroscopy was performed using a Bruker Avance III HD 300, Bruker Avance III 400, Bruker Avance NEO 600 and a Bruker Avance III HD 600 with a TCI helium CryoProbe. Samples were prepared using either DMSO-*d6*, CD_3CN or $CDCl_3$, purchased from Cambridge Isotope Laboratories, Inc and Merck. In H_2O/CD_3CN mixtures, ^{1}H NMR spectra were recorded with water suppression using the excitation sculping method. All chemical shifts were calibrated against residual solvent signals. All coupling constants (*J*) are reported in Hertz. Signals in the NMR spectra are reported as broad (br), singlet (s), doublets (d), triplets (t), quartets (q), quintets (qu), sextets (sx), septets (sept), or unclear multiplets (m). NMR spectra were processed with MestReNova 12.0.0 software or Bruker TopSpin 4.5. All NMR data are assigned unambiguously, except where specified.

Buffered solutions of 1:1 (v/v) water:acetonitrile (CH_3CN or CD_3CN) containing hosts and/or dyes were prepared as follows: sodium phosphate for pH range 5.8 to 7.8 and boric acid for pH range 8.2 to 10.2 were dissolved in MilliQ water at 20 mM or 100 mM (depending on the experiment) and the solution pH was monitored with a glass electrode probe (SevenDirect SD2, Mettler Toledo) calibrated in aqueous buffers. The final pH was adjusted with HCl 1M/0.1M and NaOH 1M/0.1M. Then, the buffered aqueous solutions were mixed with CH_3CN or CD_3CN, as pure solvents or as stock solutions of hosts and dyes as needed.

Fluorescence experiments were performed using a HORIBA Duetta Fluorescence and Absorbance Spectrometer equipped with a Peltier-based temperature-controlled sample holder and a 1 cm pathlength quartz cuvette. Emission spectra were recorded at 25 °C from 480 to 750 nm using excitation and emission slit widths of 5 nm and 10 nm, respectively. Each spectrum represents the average of three consecutive scans and was smoothed using a 50-point weighted adjacent-averaging method (applied to facilitate clear identification of the maximum emission intensity).

Data processing and analysis for fluorescence and NMR experiments were performed using Microsoft Excel for data organization and preliminary calculations, and Origin software for advanced data analysis and visualization.

ElectroSpray Ionization High-Resolution Mass Spectrometry (ESI-HRMS) analyses to confirm the molecular formula of **H-SA4**, **CN-SA4**, **Tf-SA4** and **Tf-SA4m** were performed on high resolution mass spectrometer Bruker Compact QTOF using electrospray ionization.

S1.2 Synthetic overview

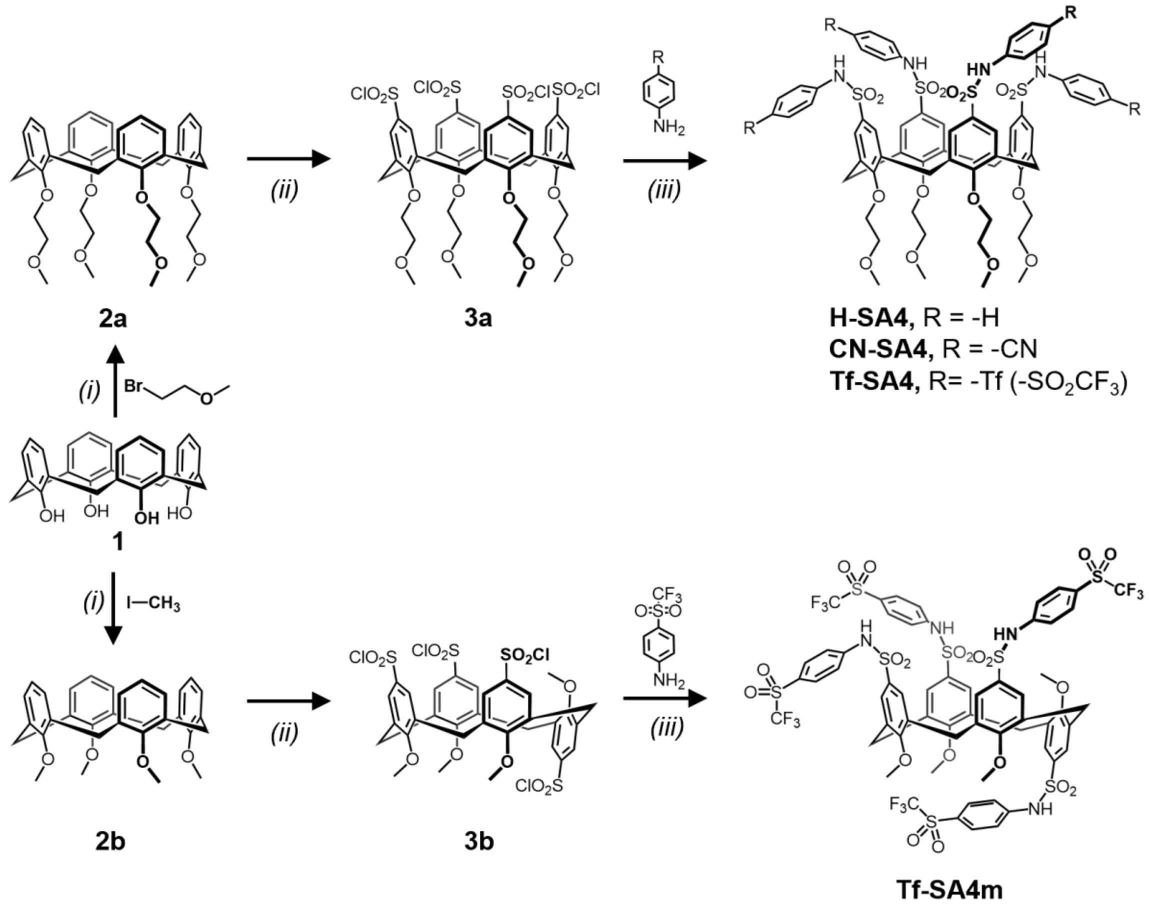

Scheme S1 Synthetic scheme **for H-SA4, CN-SA4, Tf-SA4** and **Tf-SA4m**. *(i)* NaOH (aq) 50% w/w, TBAB, toluene, reflux, 12 h. *(ii)* HSO_3Cl, CH_2Cl_2, rt, 15 m. *(iii)* Pyridine, microwaves, 70 °C, 30 m.

S2. Synthesis and characterizations of H-SA4, CN-SA4, Tf-SA4 and Tf-SA4m

p-tertbutylcalix[4]arene[38] and calix[4]arene (compound **1**)[39] were prepared according to synthetic protocols reported by Gutsche.

S2.1 Compound 2a

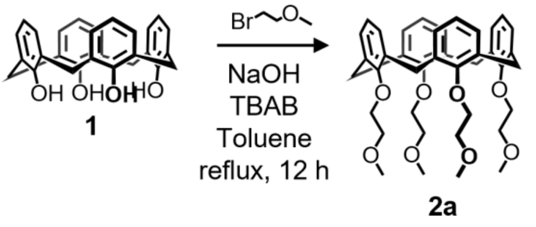

25,26,27,28-tetrakis(methoxyethoxy)calix[4]arene

Method based on Bitter (1995)[40].

425 mg of **1** (1 mmol) and 97 mg of TBAB (1.5 mmol) are completely dissolved in 25 mL of toluene. Then, 1.45 mL of 2-bromoethyl methyl ether (100 mmol) are added. The reaction mixture is brought to reflux, after which 3.3 mL of 50% w/w aqueous NaOH are added (through the top opening of the condenser). The mixture is allowed to react under reflux for 12 hours. After cooling to room temperature, it is poured into a separatory funnel, and the aqueous phase is discarded. The organic phase is washed with 2 × 20 mL of 1 M HCl, 20 mL of distilled water, and 5 mL of brine. It is then dried over anhydrous sodium sulfate and concentrated under reduced pressure. The pale yellow solid obtained was purified by DCVC[41] using 33% ethyl acetate in hexane. White powder, 355 mg, 54% yield.

NMR

^{1}H NMR (300 MHz, DMSO-*d$_6$*) δ 6.65 (d, *J* = 8.1 Hz, 8H, *m*-C**H**), 6.54 (t, *J* = 7.6 Hz, 4H, *p*-C**H**), 4.40 (d, *J* = 13.1 Hz, 4H, C-C**H$_2$**-C), 4.02 (t, *J* = 5.5 Hz, 8H, C-OC**H$_2$-**), 3.79 (t, *J* = 5.5 Hz, 8H, -C**H$_2$**-OCH$_3$), 3.34 (s, 12H, O**CH$_3$**), 3.14 (d, *J* = 13.1 Hz, 4H, C-C**H$_2$**-C).

^{13}C{^{1}H} NMR (75 MHz, DMSO-d$_6$) δ 155.95 (**C**-O), 134.52 (**C**-CH$_2$-**C**), 127.98 (*m*-C**H**), 122.00 (*p*-C**H**), 72.93 (C-O**C**H$_2$-), 71.30 (-**C**H$_2$-OCH$_3$), 58.00 (-O**C**H$_3$), 29.99 (C-**C**H$_2$-C).

The spectroscopic data are consistent with those previously reported[42] for this compound.

S2.2 Compound 3a

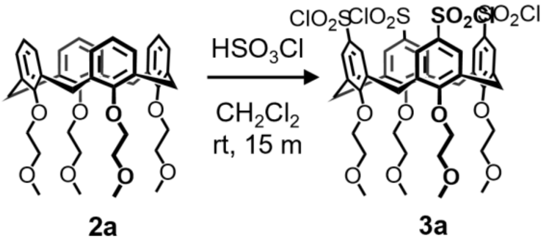

5,11,17,23-tetrakis(chlorosulfonyl)-25,26,27,28-tetrakis(methoxyethoxy)calix[4]arene

Modified method based on Morzherin (1993)[15].

1314 mg of **2a** (2 mmol) are dissolved in 4 mL of CH_2Cl_2 and cooled to 0 °C in an ice-water bath. Then, 5.3 mL of chlorosulfonic acid (80 mmol) are added dropwise, avoiding boiling of the CH_2Cl_2. Once the acid has been fully added, the reaction is stirred vigorously at room temperature for 15 minutes under an inert atmosphere. After this time, the reaction mixture is added dropwise to 50 mL of ice-water with stirring. The suspension is transferred to a separatory funnel and 50 ml of CH_2Cl_2 are added. The organic phase is separated and immediately dried over anhydrous sodium sulfate, and the aqueous phase is extracted twice more with 50 mL of CH_2Cl_2. The combined organic phases are dried again, filtered, and concentrated at 40 °C under reduced pressure (maximum 300 mbar). The solid is finally dried under a gentle stream of nitrogen. White solid, 2.081 grams, near quantitative yield. The crude product was used without further purification in the next step.

NMR

^{1}H NMR (300 MHz, DMSO-*d6*) δ 7.20 (s, 8H, C**H** calix), 4.45 (d, *J* = 12.7 Hz, 4H, C-C**H₂**-C), 4.10 (t, *J* = 5.2 Hz, 8H, C-OC**H₂**-), 3.80 (t, *J* = 5.2 Hz, 8H, -C**H₂**-OCH₃), 3.33 (m, 16H, C-C**H₂**-C + OC**H₃**).

^{13}C{^{1}H} NMR (75 MHz, DMSO-*d6*) δ 156.97 (**C**-O), 140.87 (**C**-SO₂Cl), 134.09 (C-**C**H₂-C), 126.15 (**C**H calix), 73.43 (C-O**C**H₂-), 71.22 (-**C**H₂-OCH₃), 58.09 (-O**C**H₃), 30.41 (C-**C**H₂-C).

The spectroscopic data are consistent with those previously reported[43] for this compound.

S2.3 H-SA4

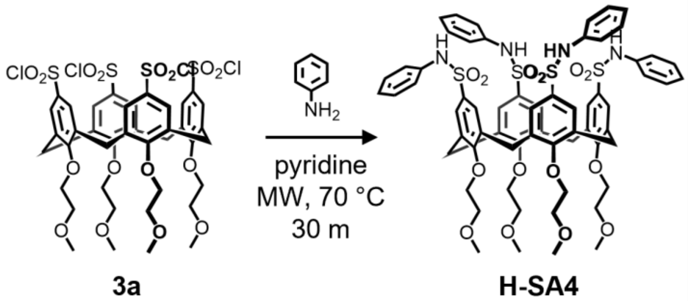

25,26,27,28-tetrakis(methoxyethoxy)-5,11,17,23-tetrakis(phenylsulfamoyl)calix[4]arene

Modified method based on Pinter (2011)[19]

0.3 mL of aniline (3.48 mmol, 3 equivalents per subunit) are dissolved in 2 mL of anhydrous pyridine in a 10 mL microwave tube equipped with a magnetic stir bar. Then, 300 mg of compound **3a** (0.29 mmol) are added and completely dissolved. The tube is purged with nitrogen and irradiated in a microwave reactor at 70 °C for 30 minutes. The reaction mixture is then cooled and added dropwise to 50 mL of 0.5 M HCl under vigorous stirring in an ice-water bath. The resulting suspension is transferred to a separatory funnel and extracted three times with 50 mL of CH_2Cl_2. The organic phase is dried over anhydrous sodium sulfate and concentrated under reduced pressure. The crude product is purified by DCVC using 3% methanol in chloroform. Crystalline white solid, 211 mg. 66% yield.

NMR

^{1}H NMR (600 MHz, DMSO-*d6*) δ 9.79 (s, 4H, SO₂N**H**), 7.21 (t, *J* = 7.4 Hz, 8H, *m*-C**H** N-aryl), 7.18 (s, 8H, C**H** calix), 7.04 (t, *J* = 7.4 Hz, 4H, *p*-C**H** N-aryl), 6.96 (d, *J* = 8.7 Hz, 8H, *o*-C**H** N-aryl), 4.39 (d, *J* = 13.2 Hz, 4H, C-C**H₂**-C), 4.10 – 4.05 (m, 8H, C-OC**H₂**-), 3.62 – 3.57 (m, 8H, -C**H₂**-OCH₃),

3.26 (d, *J* = 13.2 Hz, 4H, C-C**H₂**-C), 3.08 (s, 12H, -OC**H₃**).

$^{13}C\{^{1}H\}$ NMR (151 MHz, DMSO-*d6*) δ 159.29 (**C**-O), 137.68 (**C**-NH), 134.74 (**C**-CH_2-**C**), 134.02 (**C**SO_2NH calix), 129.01 (*m*-**C**H N-aryl), 126.90 (**C**H calix), 124.15 (*p*-**C**H N-aryl), 120.77 (*o*-**C**H N-aryl), 73.11(C-O**C**H_2-), 70.93 (-**C**H_2OCH_3), 57.65 (-O**C**H_3), 30.35 (C-**C**H_2-C).

ESI-HRMS

Formula: $C_{64}H_{67}N_4O_{16}S_4$. Expected mass [M-H]$^-$: 1275.3440 *m/z*. Experimental mass [M-H]$^-$: 1275.3354 *m/z*.

S2.4 CN-SA4

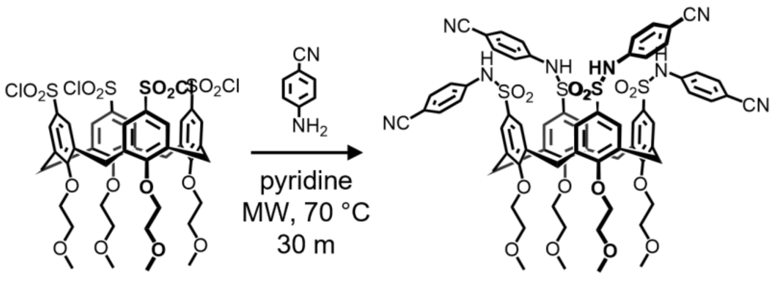

3a CN-SA4

25,26,27,28-tetrakis(methoxyethoxy)-5,11,17,23-tetrakis(4-cyanophenylsulfamoyl)calix[4]arene

Modified method based on Pinter (2011)[19]

354 mg of 4-aminobenzonitrile (3 mmol, 3 equivalents per subunit) are dissolved in 3 mL of anhydrous pyridine in a 10 mL microwave tube equipped with a magnetic stir bar. Then, 260 mg of compound **3a** (0.25 mmol) are added and completely dissolved. The tube is purged with nitrogen and irradiated in a microwave reactor at 70 °C for 30 minutes. The reaction mixture is cooled and added dropwise to 50 mL of 0.5 M HCl under vigorous stirring in an ice-water bath. The suspension is then transferred to a separatory funnel and extracted three times with 50 mL of DCM. The organic phase is dried over anhydrous sodium sulfate and concentrated under reduced pressure. The crude product is purified by DCVC using 5% methanol in chloroform. 181 mg of a white amorphous solid were obtained, with a 52% yield.

NMR

^{1}H NMR (300 MHz, DMSO-*d6*) δ 10.69 (s, 4H, SO_2N**H**), 7.67 (d, *J* = 8.8 Hz, 8H, *m*-C**H** N-aryl), 7.28 (s, 8H, C**H** calix), 7.11 (d, *J* = 8.8 Hz, 8H, *o*-C**H** N-aryl), 4.41 (d, *J* = 13.5 Hz, 4H, C-C**H₂**-C), 4.09 (t, *J* = 4.5 Hz, 8H, C-OC**H₂**-), 3.62 (t, *J* = 4.5 Hz, 8H, -C**H₂**OCH₃), 3.38 (d, *J* = 13.5 Hz, 4H, C-C**H₂**-C), 3.08 (s, 12H, -OC**H₃**).

$^{13}C\{^{1}H\}$ NMR (75 MHz, DMSO-*d6*) δ 159.71 (**C**-O), 142.45 (**C**-NH), 135.06 (**C**-CH_2-**C**), 133.56 (*m*-**C**H N-aryl), 133.48 (**C**SO_2NH calix), 126.84 (**C**H calix), 118.91 (C**C**N), 118.49 (*o*-**C**H N-aryl), 105.14 (**C**CN), 73.31 (C-O**C**H_2-), 71.03(-**C**H_2OCH_3), 57.61 (-O**C**H_3), 30.12 (C-**C**H_2-C).

ESI-HRMS

Formula: $C_{68}H_{63}N_8O_{16}S_4$. Expected mass $[M-H]^-$: 1375.3250 *m/z*. Experimental mass $[M-H]^-$: 1375.3169 *m/z*.

S2.5 Tf-SA4

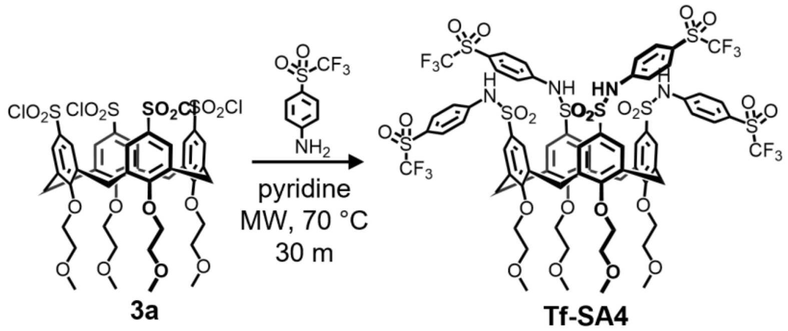

25,26,27,28-tetrakis(methoxyethoxy)-5,11,17,23-tetrakis[4-(trifluoromethylsulfonyl)phenylsulfamoyl]calix[4]arene

Modified method based on Pinter (2011)[19]

864 mg of 4-(trifluoromethylsulfonyl)aniline (3 mmol, 3 equivalents per subunit) are dissolved in 2 mL of anhydrous pyridine in a 10 mL microwave tube equipped with a magnetic stir bar. Then, 500 mg of compound **3a** (0.48 mmol) are added and completely dissolved. The tube is purged with nitrogen and irradiated in a microwave reactor at 70 °C for 30 minutes. The reaction mixture is then cooled and added dropwise to 50 mL of 0.5 M HCl under vigorous stirring in an ice-water bath. The resulting suspension is transferred to a separatory funnel and extracted three times with 50 mL of ethyl acetate. The organic phase is dried over anhydrous sodium sulfate and concentrated under reduced pressure. The crude product is purified by DCVC using 5% methanol in chloroform. White solid, 259 mg. 30% yield.

NMR

^{1}H NMR (600 MHz, CD_3CN) δ 7.94 (d, *J* = 9.1 Hz, 8H, *m*-C**H** N-aryl), 7.37 (d, *J* = 9.1 Hz, 8H, *o*-C**H** N-aryl), 7.31 (s, 8H, C**H** calix), 4.52 (d, *J* = 13.6 Hz, 4H, C-C**H$_2$**-C), 4.13 (t, *J* = 4.7 Hz, 8H, C-OC**H$_2$**-), 3.65 (t, *J* = 4.7 Hz, 8H, -C**H$_2$**OCH_3), 3.33 (d, *J* = 13.6 Hz, 4H, C-C**H$_2$**-C), 3.13 (s, 12H, -OC**H$_3$**).

^{13}C{^{1}H} NMR (151 MHz, CD_3CN) δ 161.68 (**C**-O), 147.07 (**C**-NH), 136.80 (**C**-CH_2-**C**), 134.04 (**C**-SO_2NH calix), 133.68 *m*-**C**H N-aryl, 128.32 (**C**H calix), 124.73 (**C**-SO_2CF_3), [124.09, 121.93, 119.78, 117.63, (**C**F_3)] 119.74 (*o*-**C**H N-aryl), 74.93 (C-O**C**H_2-), 72.46 (-**C**H_2OCH_3), 58.52 (-O**C**H_3), 31.28 (C-**C**H_2-C).

^{19}F NMR (565 MHz, CD_3CN) δ -79.98 (-SO_2C**F$_3$**).

ESI-HRMS

Formula: $C_{68}H_{63}F_{12}N_4O_{24}S_8$. Expected mass $[M-H]^-$: 1803.1412 m/z. Experimental mass $[M-H]^-$: 1803.1417 m/z.

S2.6 Compound 2b

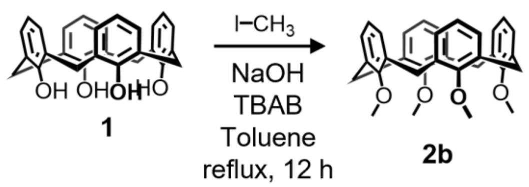

25,26,27,28-tetramethoxycalix[4]arene

Method based on Bitter (1995)[40].

2.1 grams of compound **1** (5 mmol) and 483 mg of TBAB (1.5 mmol) are completely dissolved in 125 mL of toluene. Then, 6.23 mL of iodomethane (100 mmol) are added. The reaction mixture is brought to reflux, and subsequently, 16.6 mL of 50% w/w aqueous NaOH are added through the top opening of the condenser. The mixture is allowed to react under reflux for 12 hours. After cooling to room temperature, it is poured into a separatory funnel, and the aqueous phase is discarded. The organic phase is washed with 2 × 100 mL of 1 M HCl, 100 mL of distilled water, and 25 mL of brine. It is then dried over anhydrous sodium sulfate and concentrated under reduced pressure. The resulting pale yellow solid is recrystallized in acetone and dried under vacuum. White crystals, 2 grams, 86% yield.

NMR

^{1}H NMR (400 MHz, $CDCl_3$) δ 7.55 – 6.25 (m, 12H, C**H** calix), 4.55 – 2.94 (m, 20H, C-C**H$_2$**-C + OC**H$_3$**).

^{13}C{^{1}H} NMR (101 MHz, $CDCl_3$) δ 158.04 (**C**-OCH_3), 135.19 (**C**-CH_2-**C**), 129.58-127.02 (*m*-**C**H), 122.72-121.59 (*p*-**C**H), 61.64 (C-O**C**H_3), 30.73 (C-**C**H_2-C).

The spectroscopic data are consistent with those previously reported[43] for this compound.

S2.7 Compound 3b

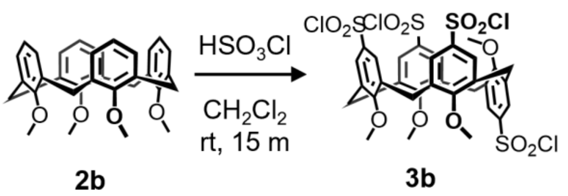

5,11,17,23-tetrakis(chlorosulfonyl)-25,26,27,28-tetramethoxycalix[4]arene

Modified method based on Morzherin (1993)[15].

962 mg of compound **2b** (2 mmol) are dissolved in 2 mL of CH_2Cl_2 and cooled to 0 °C in an ice-water bath. Then, 5.3 mL of chlorosulfonic acid are added dropwise, avoiding boiling of the CH_2Cl_2. After complete addition of the acid, the reaction mixture is stirred vigorously for 15 minutes at room temperature under an inert atmosphere. After this time, the reaction mixture is added dropwise to 50

mL of ice-water under stirring. The resulting suspension is transferred to a separatory funnel and 50 ml of CH_2Cl_2 are added. The organic phase is separated and immediately dried over anhydrous sodium sulfate. The aqueous phase is washed two more times with 50 mL of CH_2Cl_2. The combined organic extracts are dried again, filtered, and concentrated at 40 °C under reduced pressure (maximum 300 mbar). The resulting solid is finally dried under a gentle stream of nitrogen. White solid, 1.61 grams, 92% yield. The crude product was used without further purification in the next step.

NMR

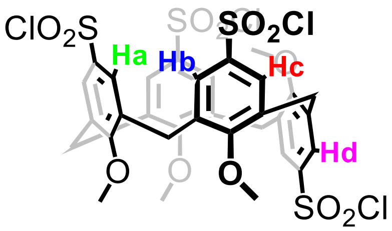

^{1}H NMR (300 MHz, $CDCl_3$) δ 8.05 (s, 2H, C-**Ha**), 7.93 (s, 2H, C-**Hd**), 7.68 (d, *J* = 2.4 Hz, 2H, C-**Hc**), 7.17 (d, *J* = 2.4 Hz, 2H, C-**Hb**), 4.17 (d, *J* = 14.1 Hz, 2H, C-C**H₂**-C), 3.91 (m, 7H, 2 x C-C**H₂**-C + OC**H₃**), 3.86 (s, 6H, 2 x OC**H₃**), 3.42 (d, *J* = 14.1 Hz, 2H, C-C**H₂**-C), 3.19 (s, 3H, OC**H₃** in cavity).

The spectroscopic data are consistent with those previously reported[44] for this compound.

S2.8 Tf-SA4m

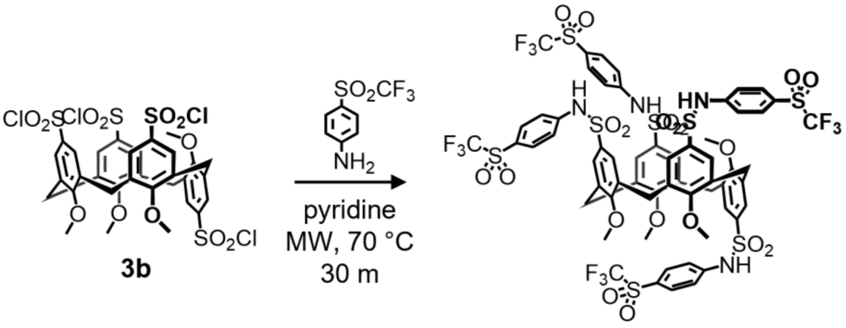

25,26,27,28-tetramethoxy-5,11,17,23-tetrakis(4-(trifluoromethylsulfonyl)phenylsulfamoyl)calix[4]arene

Modified method based in Pinter (2011)[19]

621 mg of 4-(trifluoromethylsulfonyl)aniline (2.76 mmol, 3 eq. per subunit) are dissolved in 2 mL of anhydrous pyridine in a 10 mL microwave tube with magnetic stir bar. Subsequently, 200 mg of compound **3b** (0.23 mmol) are added and completely dissolved. The tube is purged with nitrogen and irradiated under microwave at 70 °C for 30 minutes. The reaction mixture is cooled and added dropwise over 50 mL of 0.5 M HCl under vigorous stirring and in an ice-water bath. The resulting suspension is transferred to a separatory funnel and extracted 3 times with 50 mL of ethyl acetate. The organic phase is dried over anhydrous sodium sulfate and concentrated under reduced pressure. The crude product is purified by DCVC using 5% methanol in chloroform. White solid, 35 mg, 10% yield.

NMR

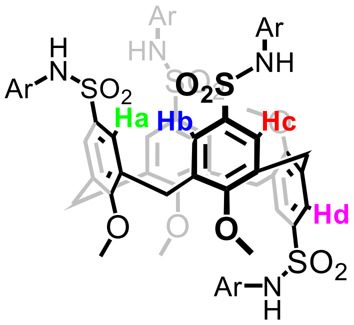

^{1}H and ^{13}C NMR resonances were assigned based in ^{1}H-^{13}C HSQC and DEPT-135 experiment. The product retained the *partial cone* conformation from compound **3b**.

^{1}H NMR (600 MHz, CD_3CN) δ 7.96 – 7.89 (m, 10H, *m*-C**H** N-aryl + C**Ha** calix), 7.78 (s, 2H, C**Hd** calix), 7.57 (d, *J* = 8.8 Hz, 2H, *o*-C**H** N-aryl), 7.51 (d, *J* = 2.5 Hz, 2H, C**Hc** calix), 7.50 – 7.45 (m, 6H, o-CH N-aryl), 7.05 (d, *J* = 2.5 Hz, 2H, C**Hb** calix), 3.87 – 3.81 (m, 4H, C-C**H2**-C), 3.72 (d, *J* = 14.4 Hz, 2H, C-C**H2**-C), 3.58 (s, 6H, -OC**H3**), 3.32 – 3.26 (m, 5H, C-C**H2**-C + -OC**H3**), 2.47 (s, 3H, -OC**H3**)

$^{13}C\{^{1}H\}$ NMR (151 MHz, CD_3CN) δ [163.12, 162.85, 162.45 (**C**-O)], [147.67, 147.40, 147.03 (**C**-NH)], [137.96, 135.87, 135.43 (**C**-CH_2-**C**)], [134.67, 134.36, 134.34 (**C**-SO_2NH)], [133.76, 133.66, 133.64 (*m*-**C**H N-aryl)], [130.70 (**C**Ha), 129.52 (**C**Hd), 128.64 (**C**Hc), 128.19 (**C**Hb)], [124.93, 124.32, 124.21, 121.94, 121.85 (**C**-SO_2CF_3 + C-SO_2**C**F_3], [119.97, 119.79, 119.60, 119.39 (*o*-**C**H N-aryl], [61.91, 60.52, 59.31 (-O**C**H_3], [36.10, 30.64 (C-**C**H_2-C)].

^{19}F NMR (565 MHz, CD_3CN) δ [-79.96, -79.99, -80.03 (-SO_2C**F3**)].

ESI-HRMS

Formula: $C_{60}H_{47}F_{12}N_4O_{20}S_8$. Expected mass $[M-H]^-$: 1627.0363 *m/z*. Experimental mass $[M-H]^-$: 1627.0349 *m/z*.

S2.9 ^{1}H NMR spectrum of H-SA4 in DMSO-d_6

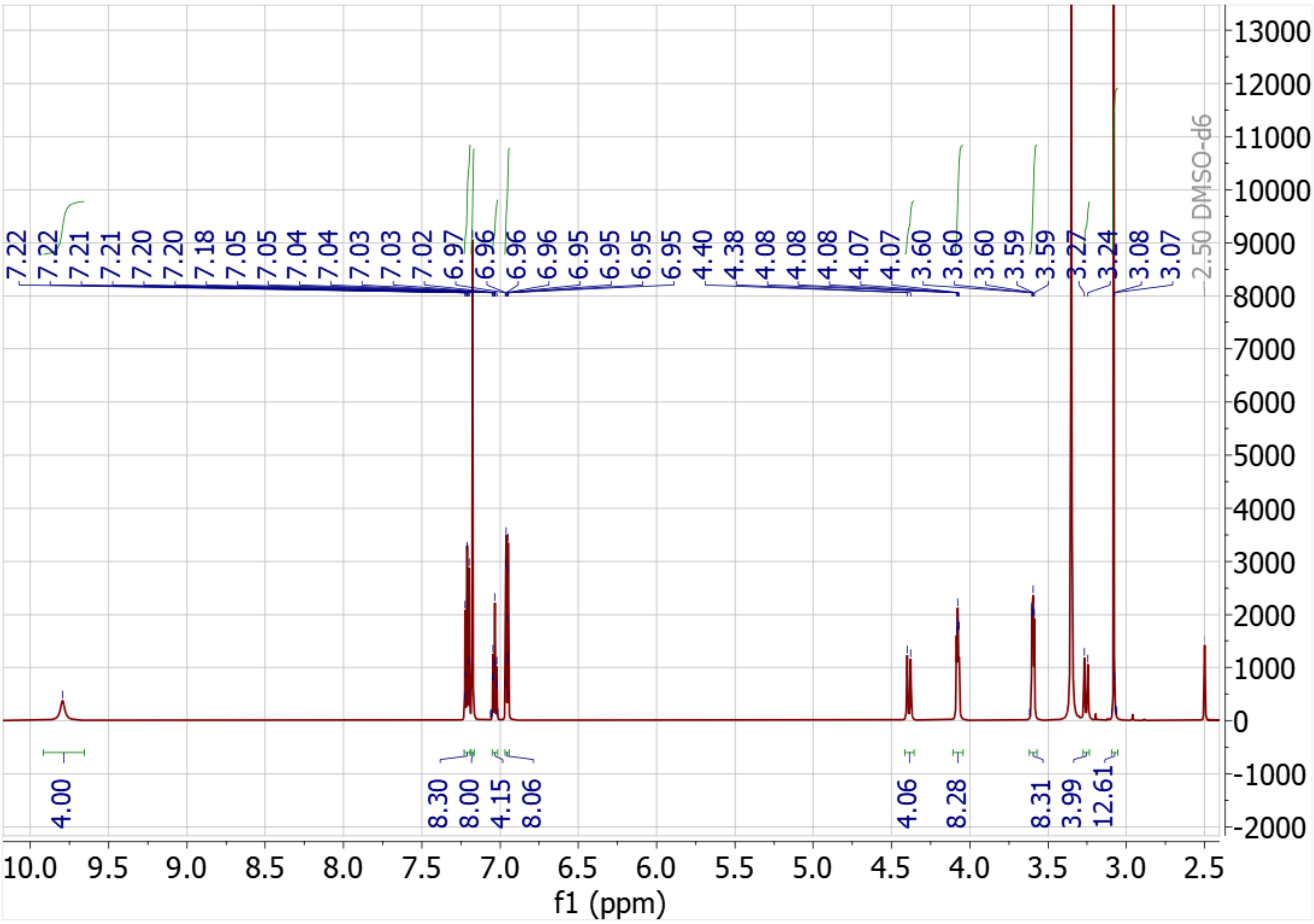

Figure S1. ^{1}H NMR (600 MHz, DMSO-d_6, 298 K) spectrum of **H-SA4**.

S2.1 $^{13}C\{^1H\}$ NMR spectrum of H-SA4 in DMSO-d_6

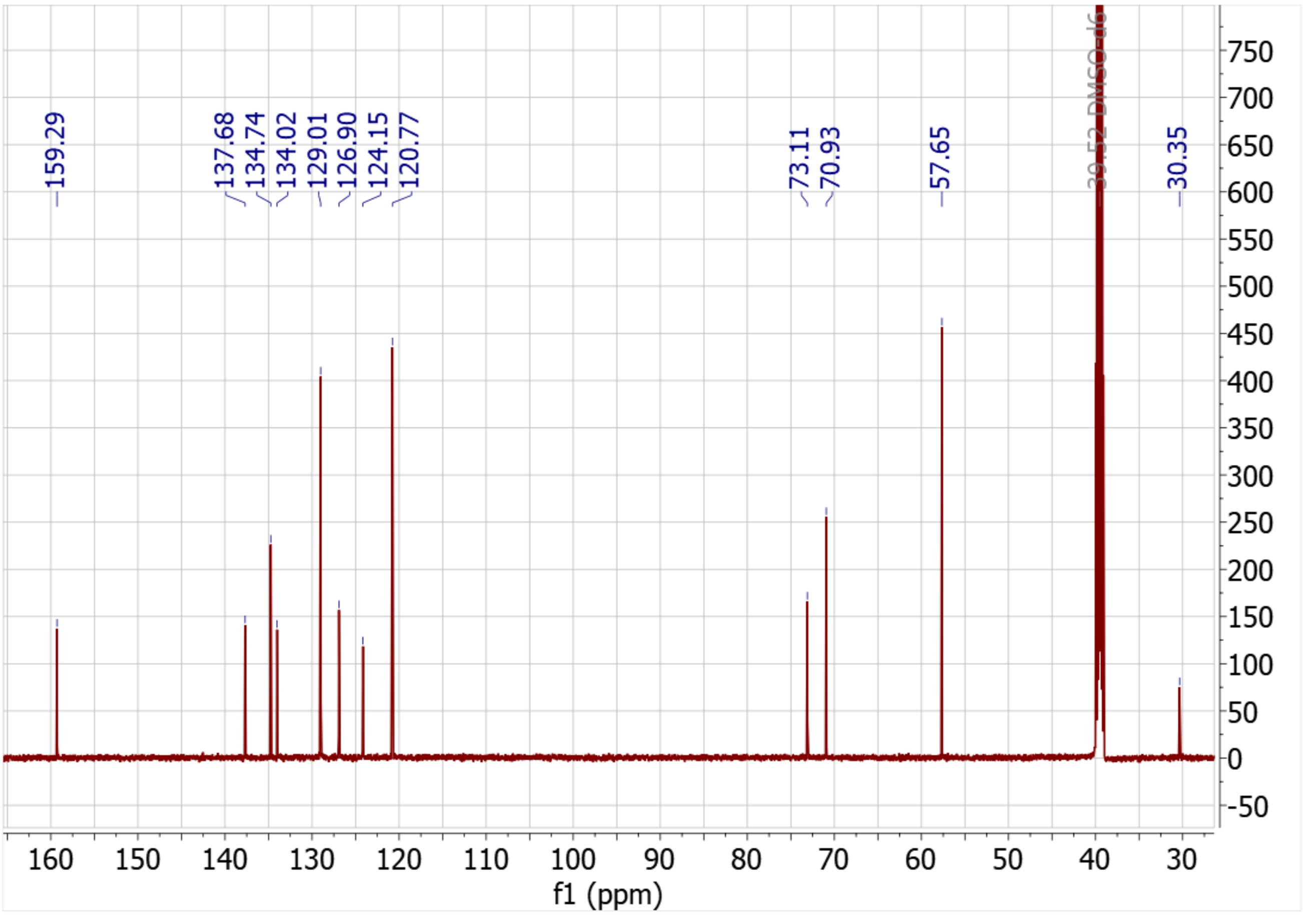

Figure S2. $^{13}C\{^1H\}$ NMR (151 MHz, DMSO-d_6, 298 K) spectrum of **H-SA4**.

S2.2 ^{1}H NMR spectrum of CN-SA4 in DMSO-d_6

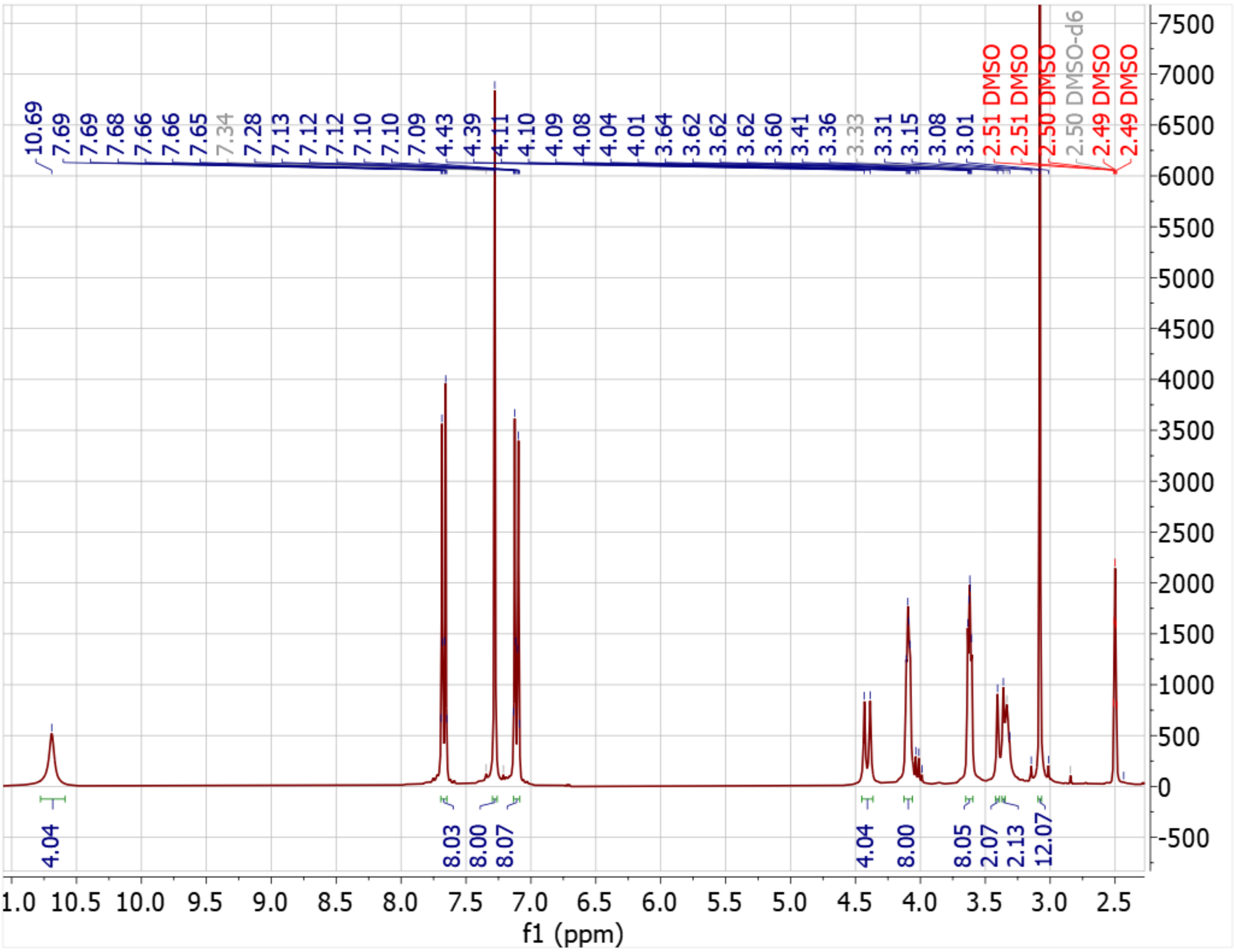

Figure S3 ^{1}H NMR (151 MHz, DMSO-d_6, 298 K) spectrum of **CN-SA4**.

S2.3 $^{13}C\{^{1}H\}$ NMR spectrum of CN-SA4 in DMSO-d_6

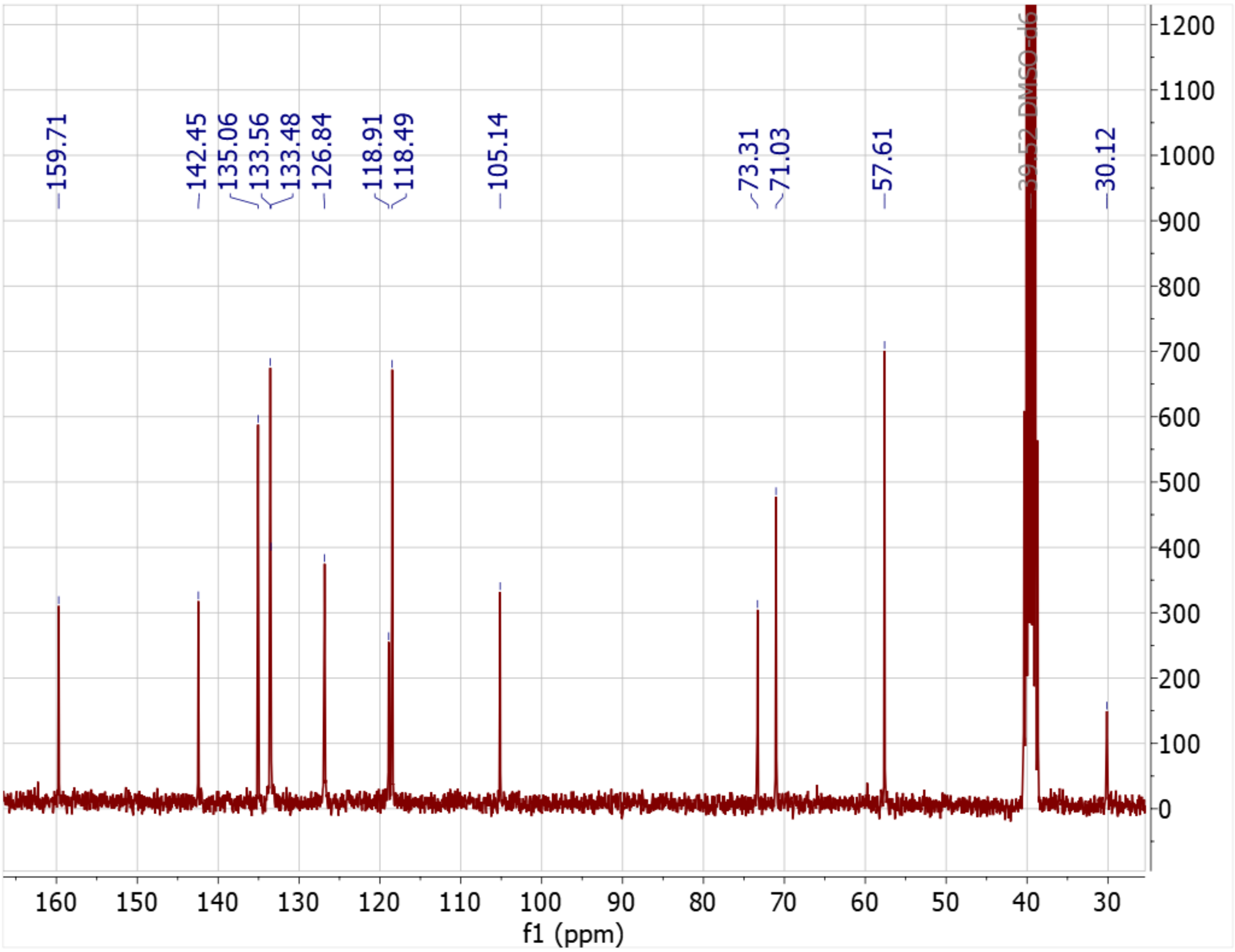

Figure S4 $^{13}C\{^{1}H\}$ NMR (151 MHz, DMSO-d_6, 298 K) spectrum of **CN-SA4**.

S2.4 ^{1}H NMR spectrum of Tf-SA4 in CD_3CN

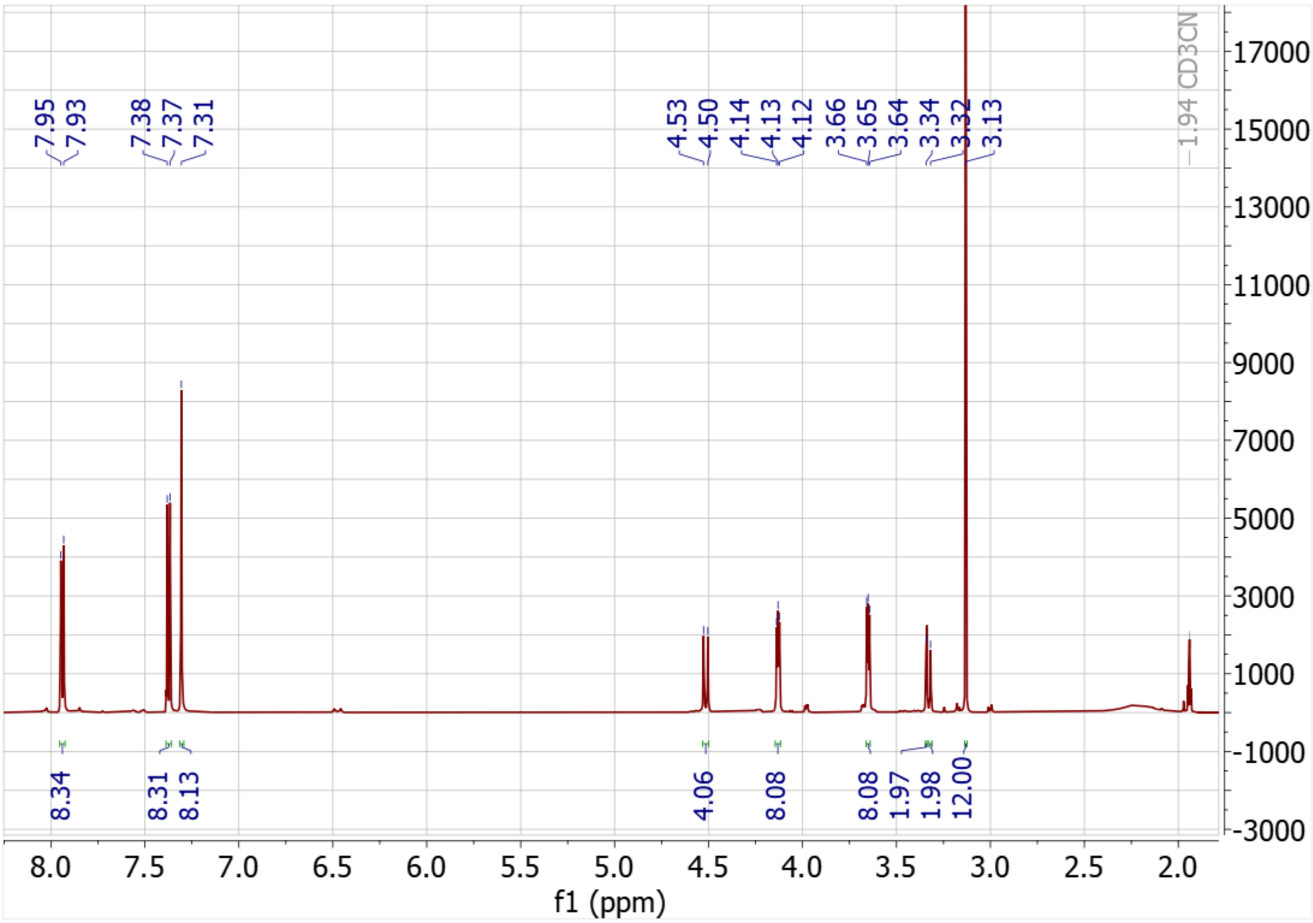

Figure S5 ^{1}H NMR (151 MHz, CD_3CN, 298 K) spectrum of **Tf-SA4**.

S2.5 $^{13}C\{^1H\}$ NMR spectrum of Tf-SA4 in CD_3CN

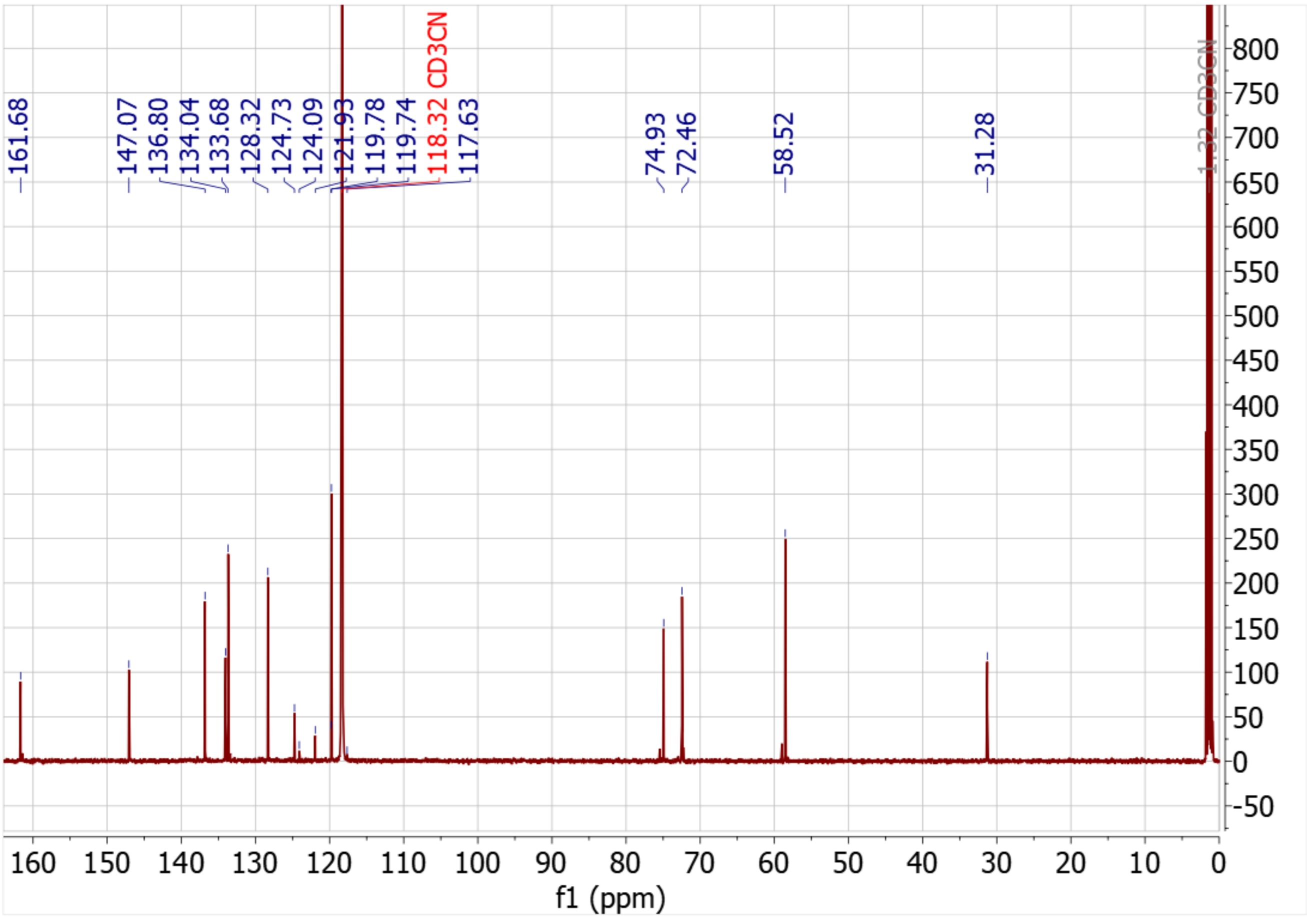

Figure S6 $^{13}C\{^1H\}$ NMR (151 MHz, CD_3CN, 298 K) spectrum of **Tf-SA4**.

S2.6 ^{1}H-^{13}C HSQC NMR spectrum of Tf-SA4 in CD_3CN

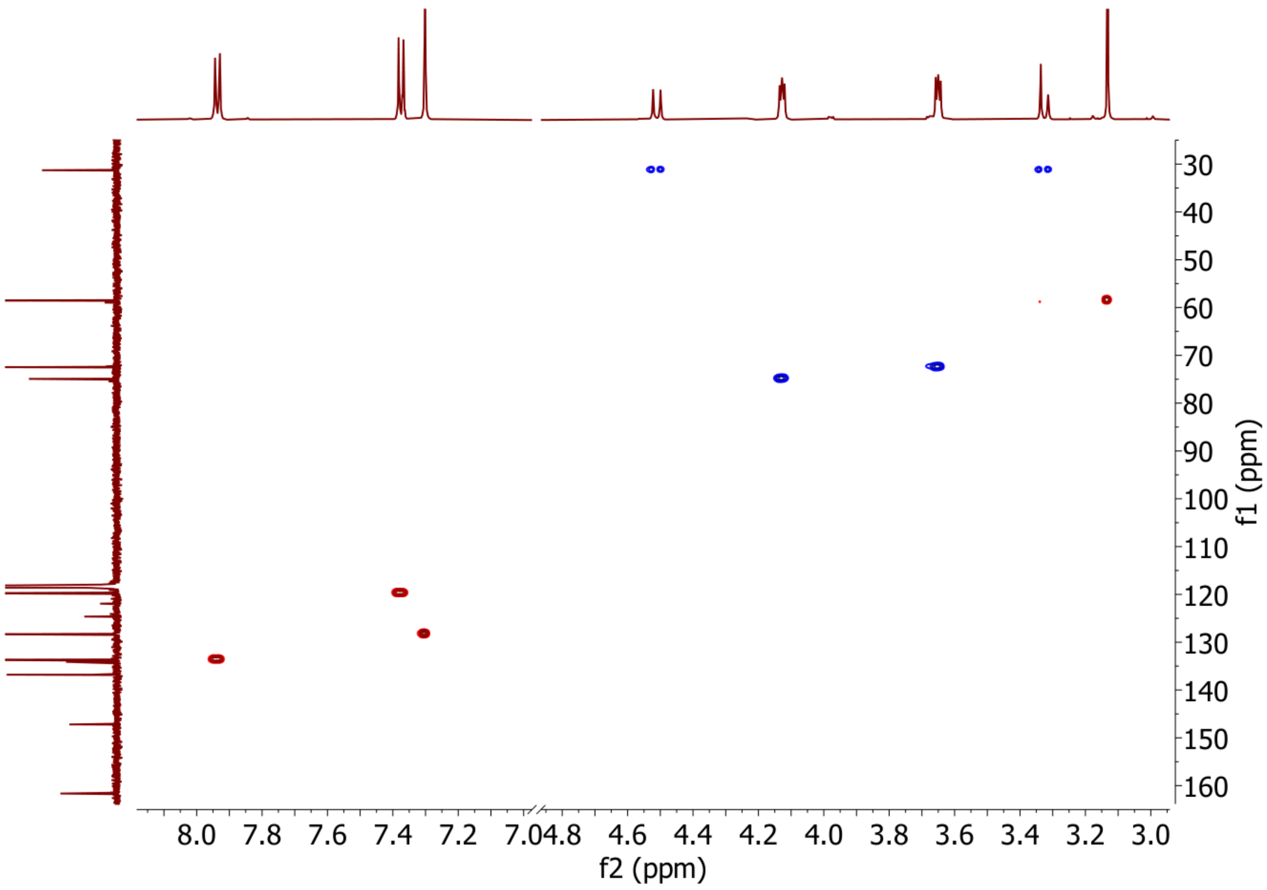

Figure S7 ^{1}H-^{13}C HSQC NMR (151 MHz, CD_3CN, 298 K) spectrum of **Tf-SA4**.

S2.7 DEPT-135 NMR spectrum of Tf-SA4 in CD_3CN

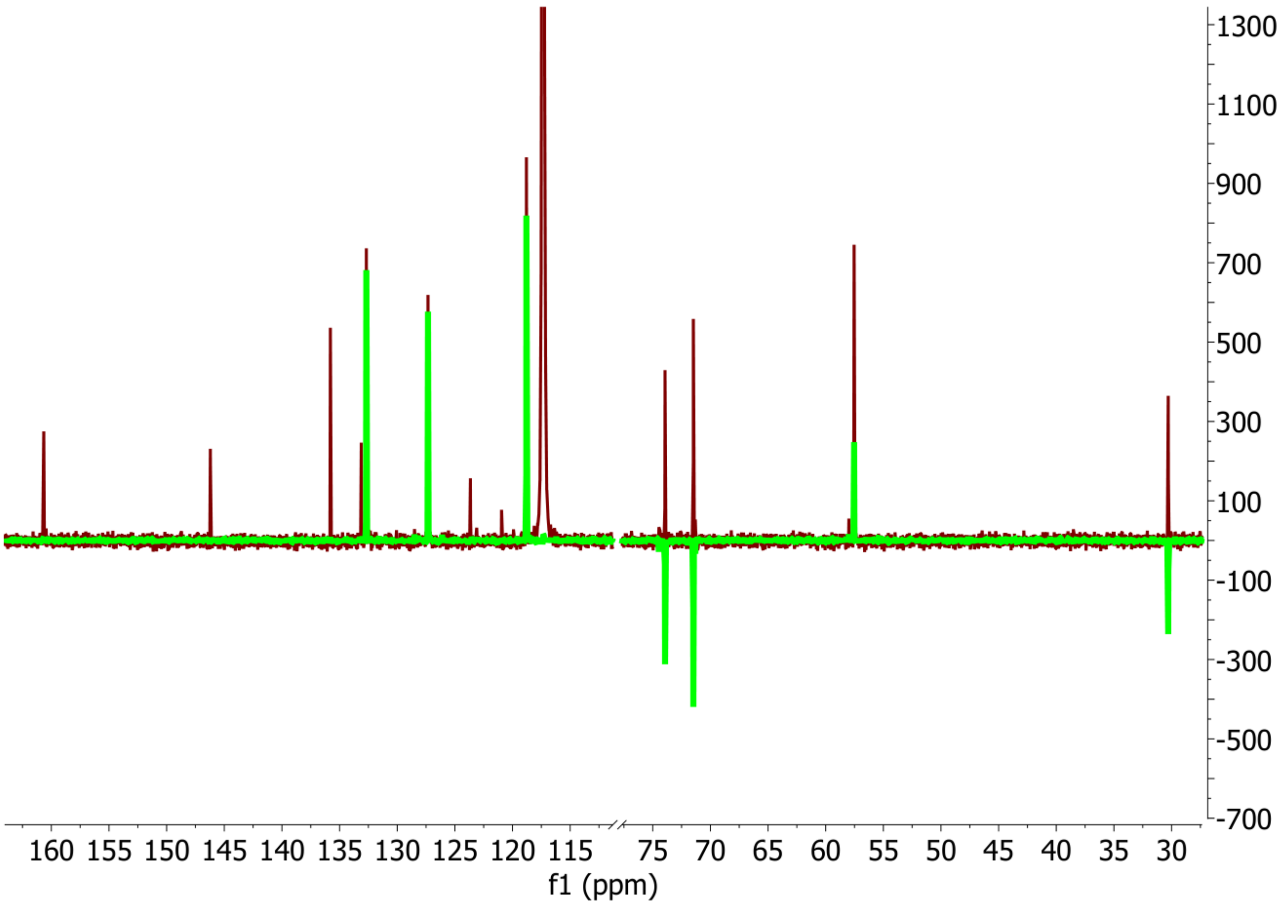

Figure S8 ^{13}C DEPT-135 NMR (151 MHz, CD_3CN, 298 K, in green over the standard ^{13}C spectrum in dark red) spectrum of **Tf-SA4**.

S2.8 ^{19}F NMR spectrum of Tf-SA4 in CD_3CN

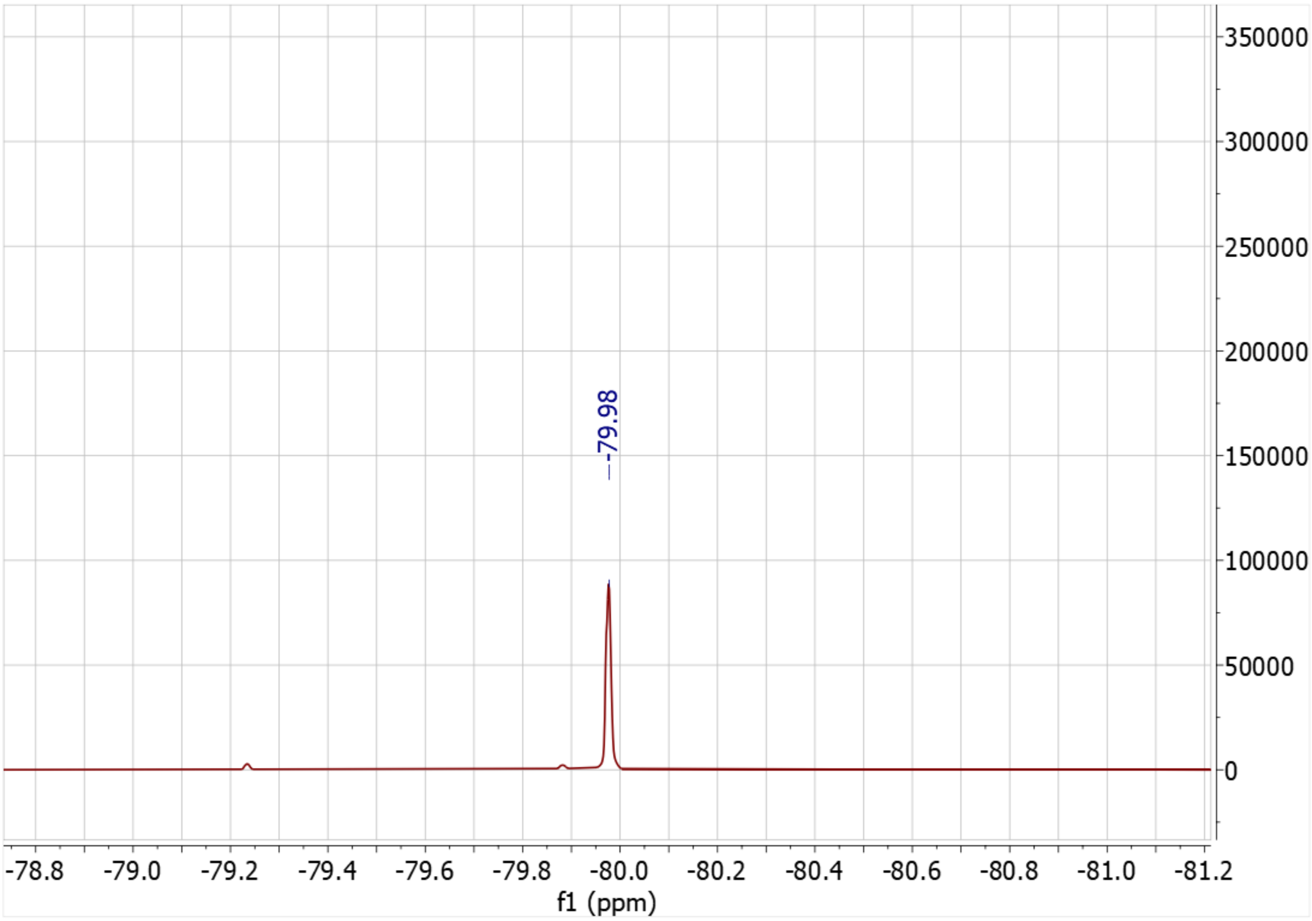

Figure S9 ^{19}F NMR (595 MHz, CD_3CN, 298 K) spectrum of **Tf-SA4**.

S2.9 ^{1}H NMR spectrum of Tf-SA4m in CD_3CN

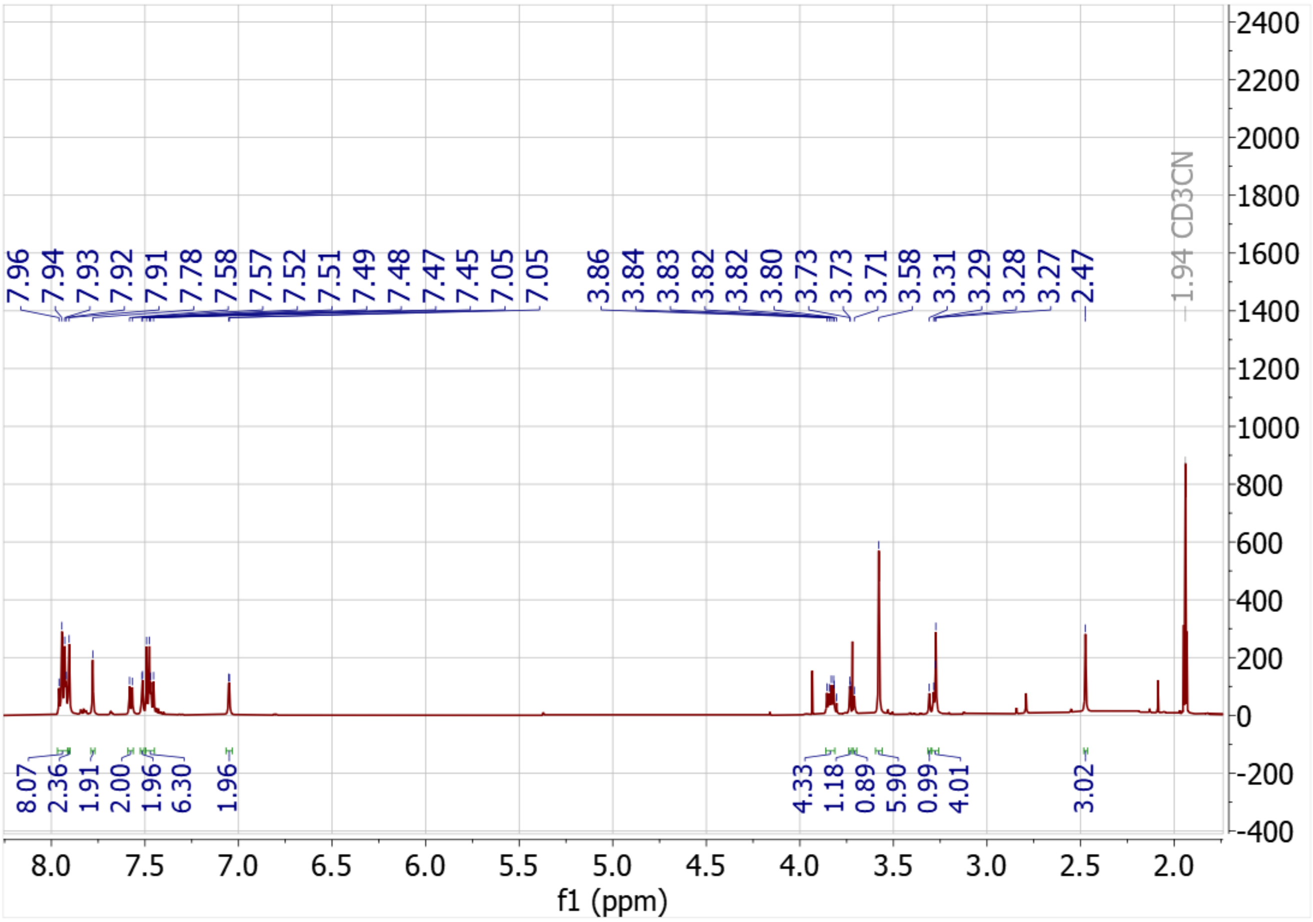

Figure S10 ^{1}H NMR (151 MHz, CD_3CN, 298 K) spectrum of **Tf-SA4m**.

S2.10 $^{13}C\{^1H\}$ NMR spectrum of Tf-SA4m in CD_3CN

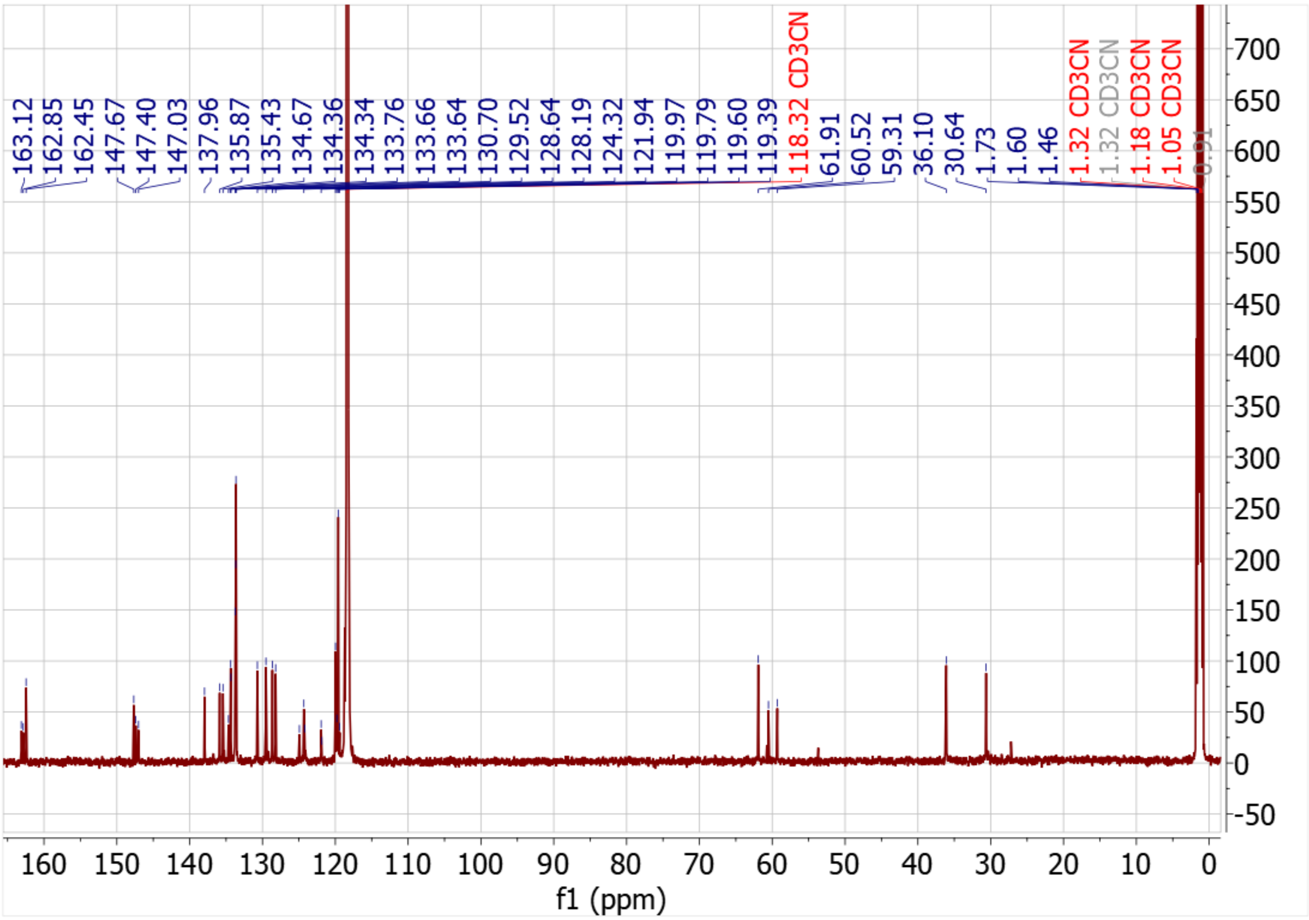

Figure S11 $^{13}C\{^1H\}$ NMR (151 MHz, CD_3CN, 298 K) spectrum of **Tf-SA4m**.

S2.11 ^{1}H-^{13}C HSQC NMR spectrum of Tf-SA4m in CD_3CN

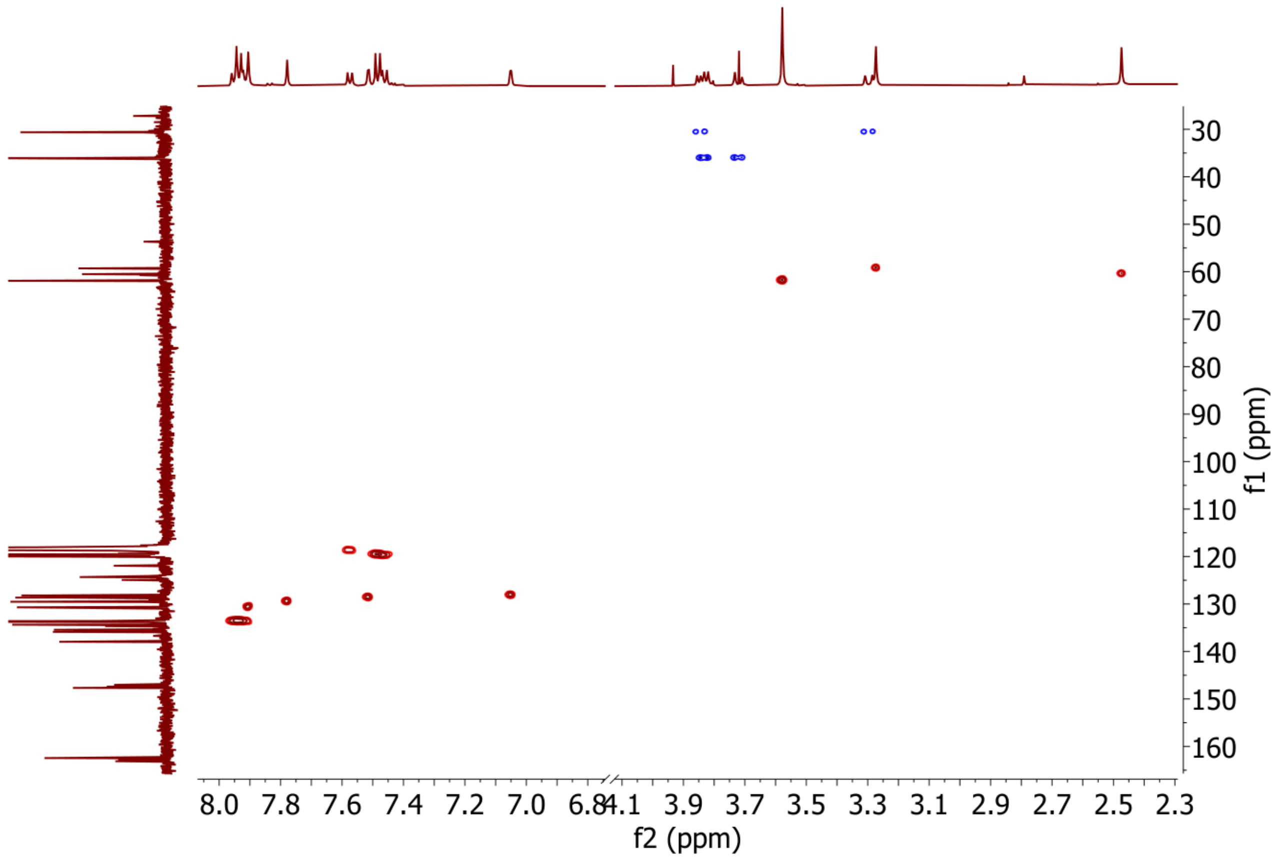

Figure S12 ^{1}H-^{13}C HSQC NMR (151 MHz, CD_3CN, 298 K) spectrum of **Tf-SA4m**.

S2.12 DEPT-135 NMR spectrum of Tf-SA4m in CD_3CN

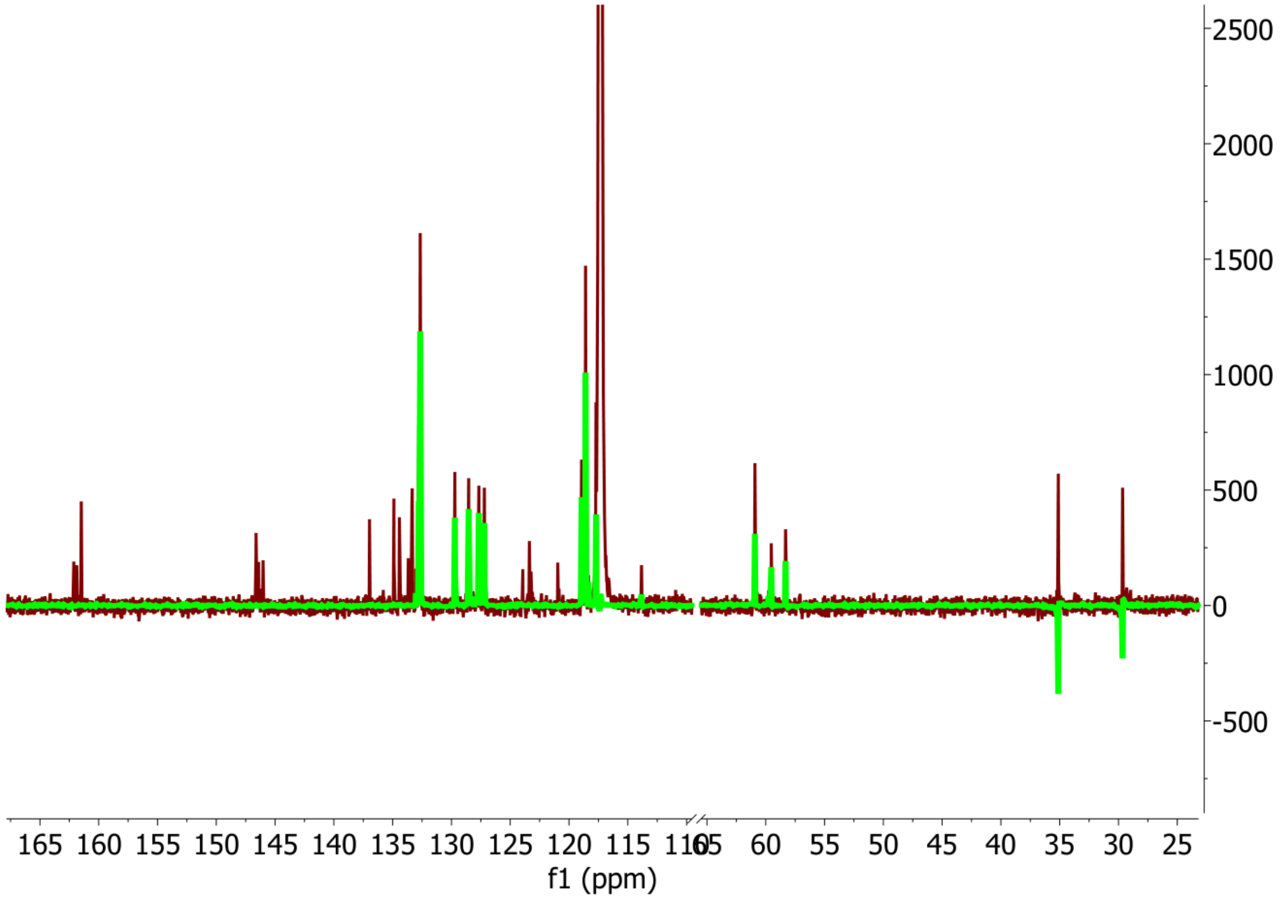

Figure S13 ^{13}C DEPT-135 NMR (151 MHz, CD_3CN, 298 K, in green over the standard ^{13}C spectra in dark red) spectra of **Tf-SA4m**.

S2.13 ^{19}F NMR spectrum of Tf-SA4m in CD_3CN

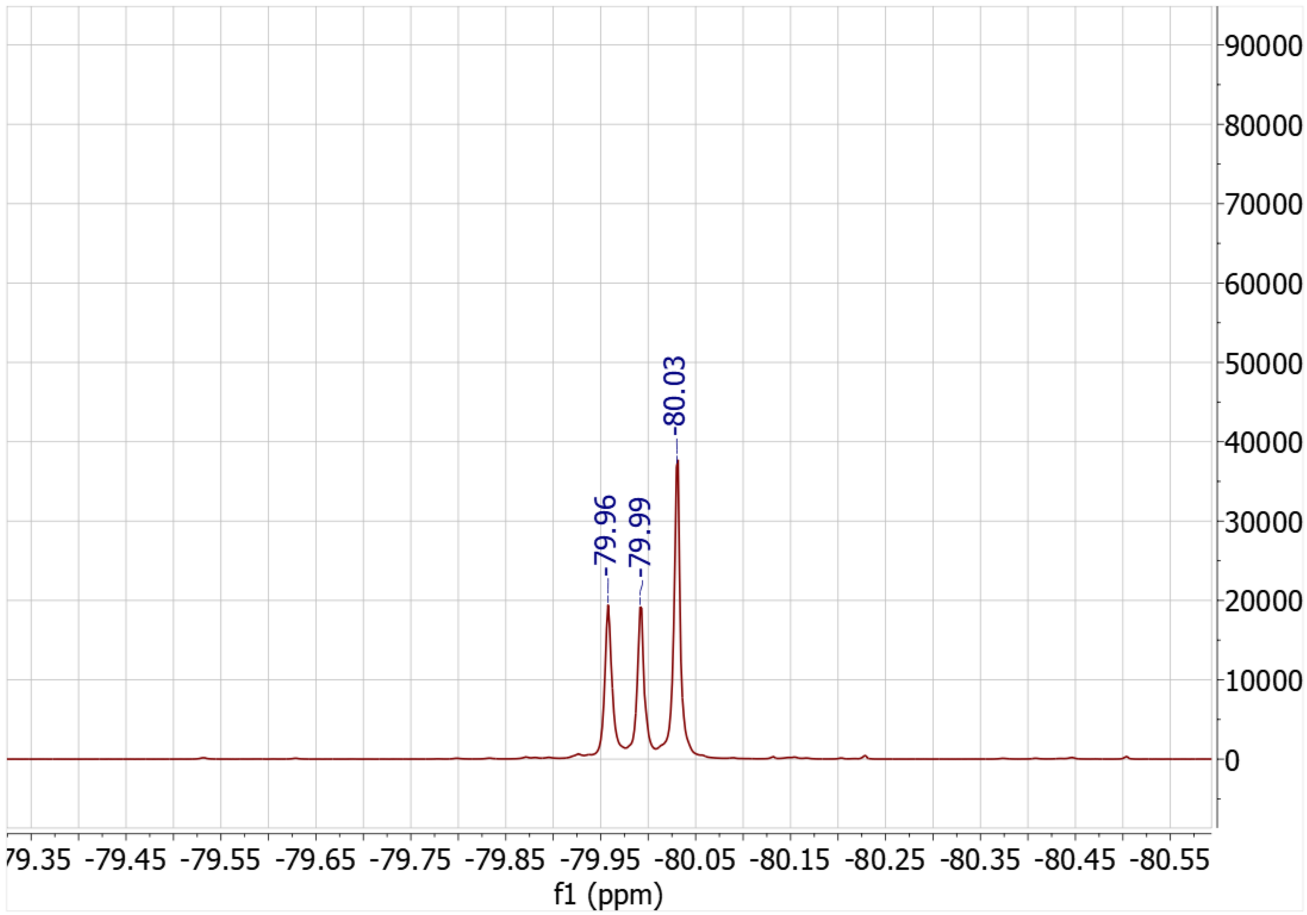

Figure S14 ^{19}F NMR (595 MHz, CD_3CN, 298 K) spectrum of **Tf-SA4m**.

S2.14 ESI-HRMS spectrum of H-SA4

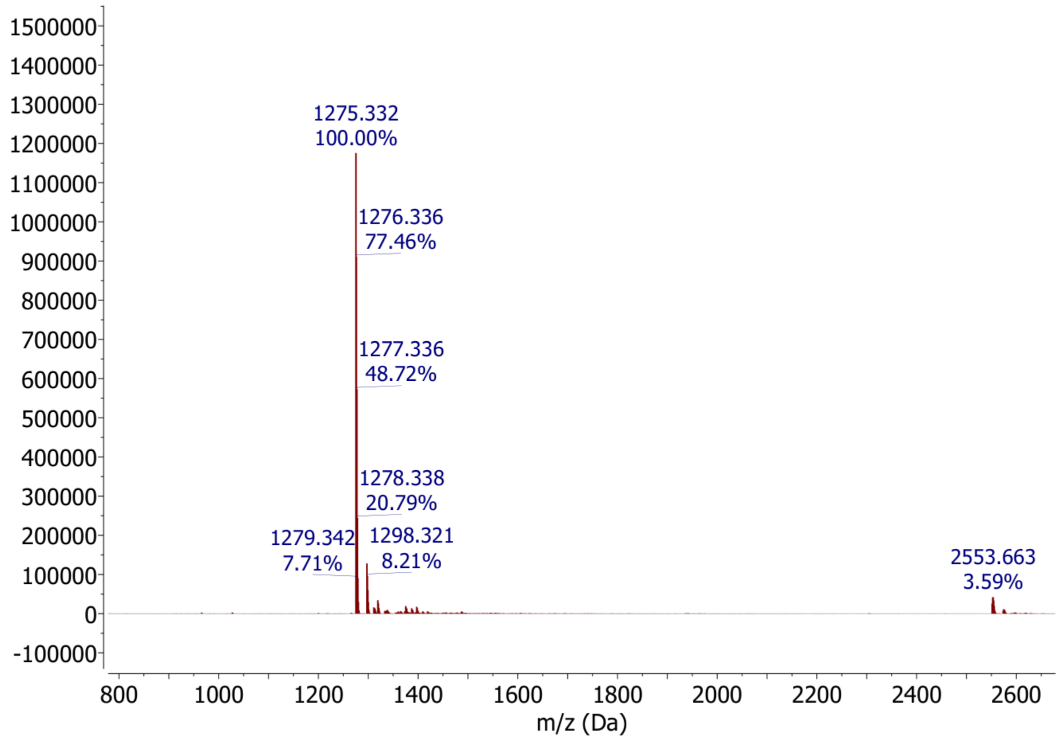

Figure S15 ESI-HRMS (negative mode) spectrum **of H-SA4**.

S2.15 ESI-HRMS spectrum of CN-SA4

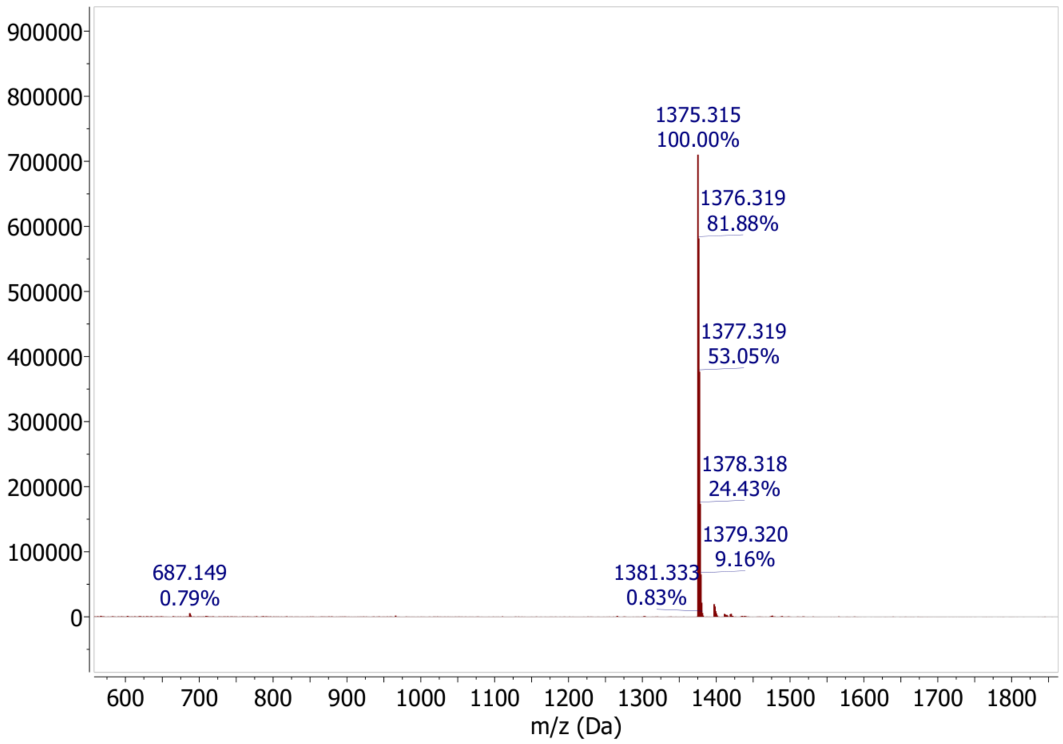

Figure S16 ESI-HRMS (negative mode) spectrum of **CN-SA4**.

S2.16 ESI-HRMS spectrum of Tf-SA4

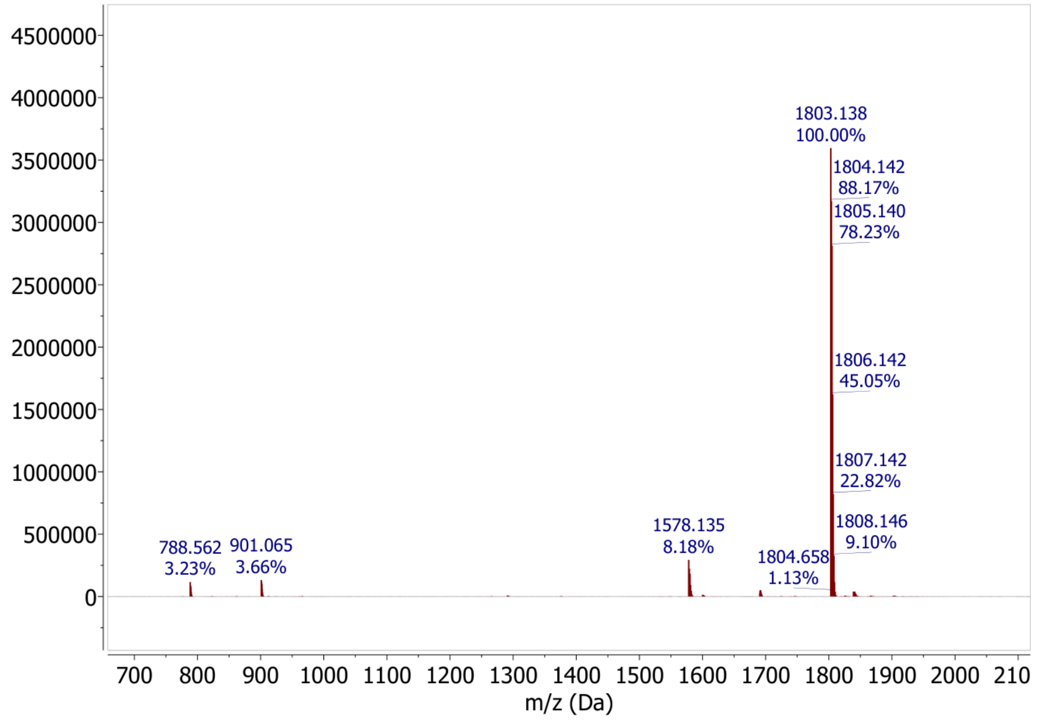

Figure S17 ESI-HRMS (negative mode) spectrum of **Tf-SA4**. 1758.135 *m/z* and 788.562 *m/z* peaks are charged fragmented species of **Tf-SA4**.

S2.17 ESI-HRMS spectrum of Tf-SA4m

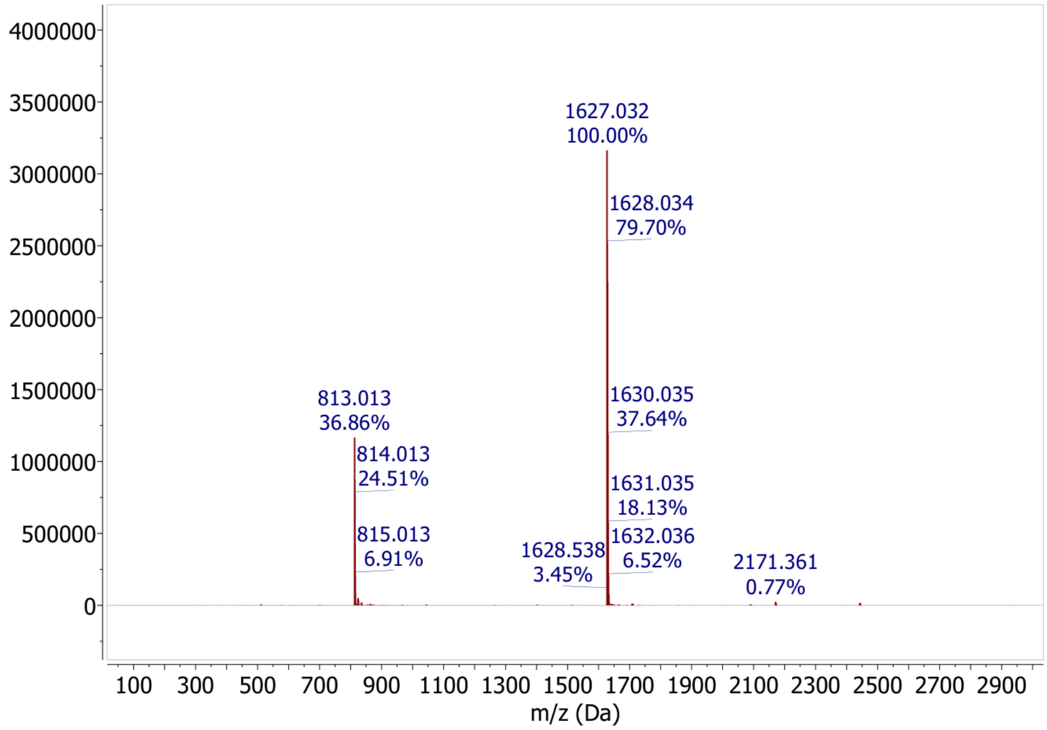

Figure S18 ESI-HRMS (negative mode) spectrum of **Tf-SA4m**.

S3. ^{1}H and ^{19}F NMR spectrum of Tf-SA4 at different pH$_{app}$ values

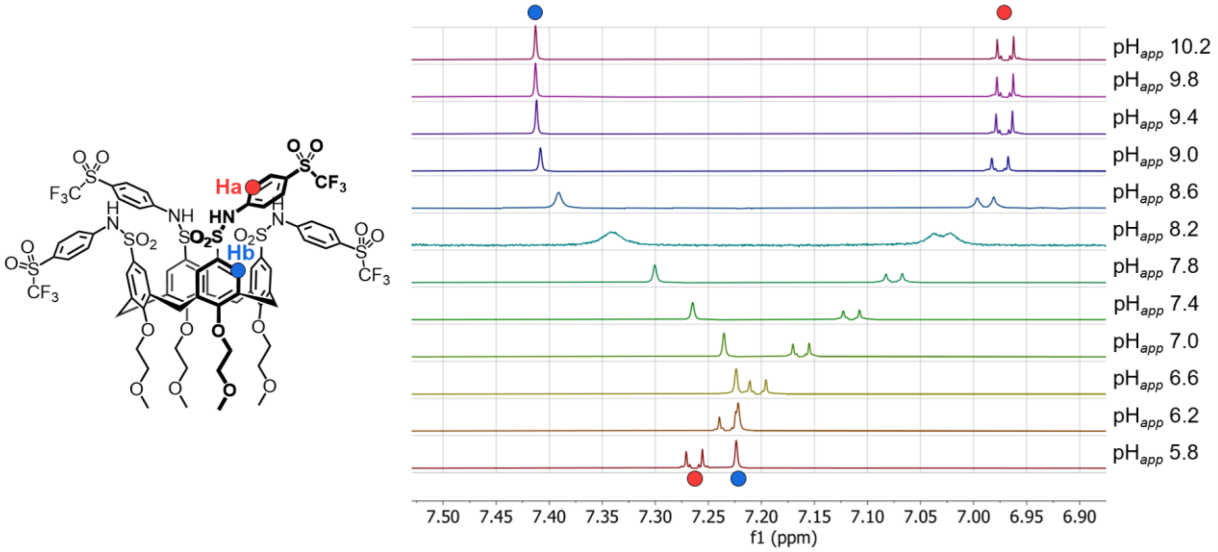

Figure S19. Partial ^{1}H NMR (600 MHz, 298K, CD_3CN:H_2O 1:1 (v/v)) spectrum of **Tf-SA4** showing Ha and Hb chemical shift perturbations across pH$_{app}$ 5.8 to 10.2 (Sodium phosphate for pH 5.8 to 7.8 and sodium borate for pH 8.2 to 10.2. Buffer concentration 10 mM).

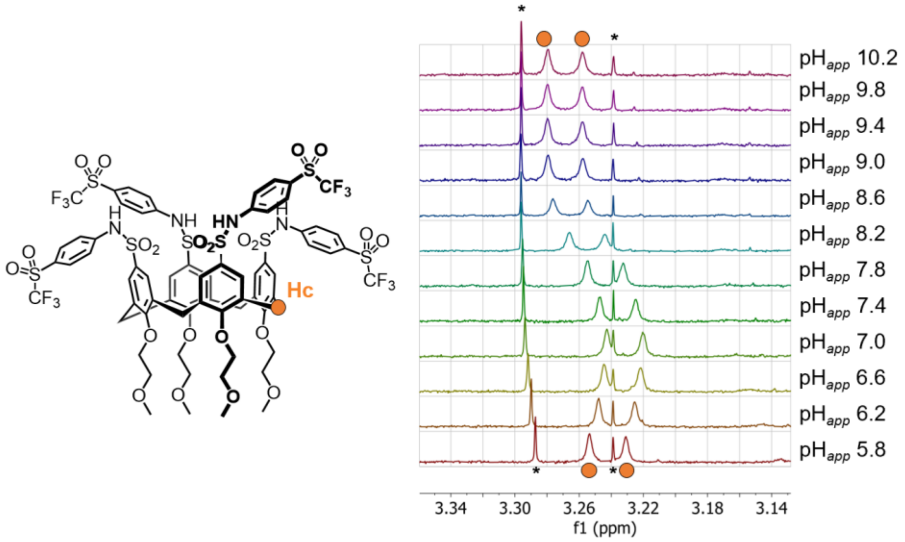

Figure S20 Partial ^{1}H NMR (600 MHz, 298K, CD_3CN:H_2O 1:1 (v/v)) spectrum of **Tf-SA4** showing Hc chemical shift perturbations across pH$_{app}$ 5.8 to 10.2 (Sodium phosphate for pH 5.8 to 7.8 and sodium borate for pH 8.2 to 10.2. Buffer concentration 10 mM). (* = Methanol traces, slow exchange C**H$_3$**OH/C**H$_3$**OD.)

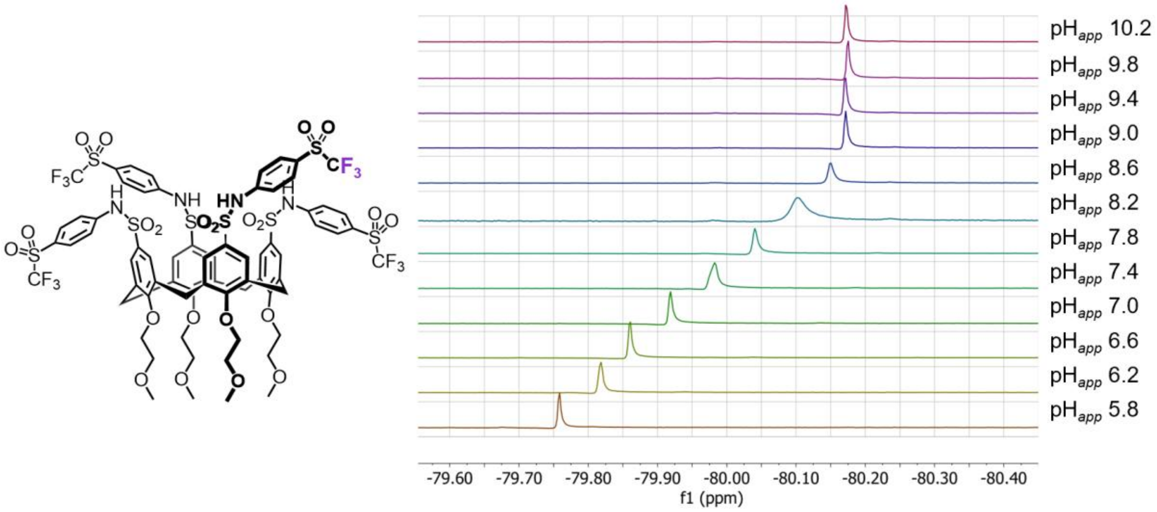

Figure S21 Partial ^{19}F NMR (595 MHz, 298K, CD_3CN:H_2O 1:1 (v/v)) spectrum of **Tf-SA4** showing F_3 chemical shift perturbations across pH$_{app}$ 5.8 to 10.2 (Sodium phosphate for pH 5.8 to 7.8 and sodium borate for pH 8.2 to 10.2. Buffer concentration 10 mM).

S4. X-ray crystallography data

S4.1 Single crystal X-ray structure of H-SA4$^{(0)}$

Colourless plates of **H-SA4** were obtained after concentration in a rotary evaporator after purification using DCVC with 5% methanol in chloroform. The crystal of **H-SA4$^{(0)}$** with dimensions of 0.039 x 0.181 x 0.367 mm, selected under the polarising microscope (Leica M165Z), was picked up on a MicroMount (MiTeGen, USA) consisting of a thin polymer tip with a wicking aperture. The X-ray diffraction measurements were carried out on a Bruker D8Quest diffractometer at 120 K using IμS Incoatec Microfocus Source with Mo-Kα radiation (λ = 0.710723 Å). The single crystal, mounted on the goniometer using a cryo loop for intensity measurements, was coated with immersion oil type NVH and then quickly transferred to the cold nitrogen stream generated by an Oxford Cryostream 800 series. Symmetry-related absorption corrections using the program SADABS[45] were applied and the data were corrected for Lorentz and polarisation effects using Bruker APEX4 software.[45] The structure was solved by program SHELXT[46] (with intrinsic phasing) and the full-matrix least-square refinements were carried out using SHELXL[47] through Olex2 suite of software[48]. The non-hydrogen atoms were refined anisotropically and H-atoms were refined isotropically and were fixed at stereo chemically reasonable positions. Crystallographic data are summarised in Table S1.

	**H-SA4$^{(0)}$**
**Crystal data**	
**Chemical formula**	$C_{64}H_{68}N_4O_{16}S_4 \cdot 2(H_2O)$
**Mr**	1313.49
**Crystal system, space group**	Monoclinic, *P21/c*
**Temperature (K)**	120
a, b, c **(Å)**	21.9582 (11), 15.4314 (9), 20.0065 (9)
**β (°)**	109.032 (2)
V **(Å^3)**	6408.5 (6)
Z	4
**Radiation type**	Mo *K*α
**μ (mm^{-1})**	0.22
**Crystal size (mm)**	0.37 × 0.18 × 0.04
Data collection	
**Diffractometer**	Bruker D8 Quest
**Absorption correction**	Multi-scan<br>SADABS (Bruker,2021) was used for absorption correction. wR2(int) was 0.1162 before and 0.0780 after correction. The Ratio of minimum to maximum transmission is 0.9020. The λ/2 correction factor is Not present.
T_{min}, T_{max}	0.672, 0.745
**No. of measured, independent and observed [I > 2σ(I)] reflections**	265339, 11393, 9572
R_{int}	0.127
**(sin θ/λ)$_{max}$ (Å^{-1})**	0.597
**Refinement**	
$R[F^2 > 2\sigma(F^2)]$, $wR(F^2)$, S	0.066, 0.149, 1.09
**No. of reflections**	11393
**No. of parameters**	861
**No. of restraints**	8
**H-atom treatment**	H atoms treated by a mixture of independent and constrained refinement
	$w = 1/[\sigma^2(F_o^2) + (0.0451P)^2 + 18.1532P]$<br>where $P = (F_o^2 + 2F_c^2)/3$
$\Delta\rho_{max}$, $\Delta\rho_{min}$ (e Å^{-3})	1.57, −0.91

Table S1 Crystallographic details of H-SA4$^{(0)}$.

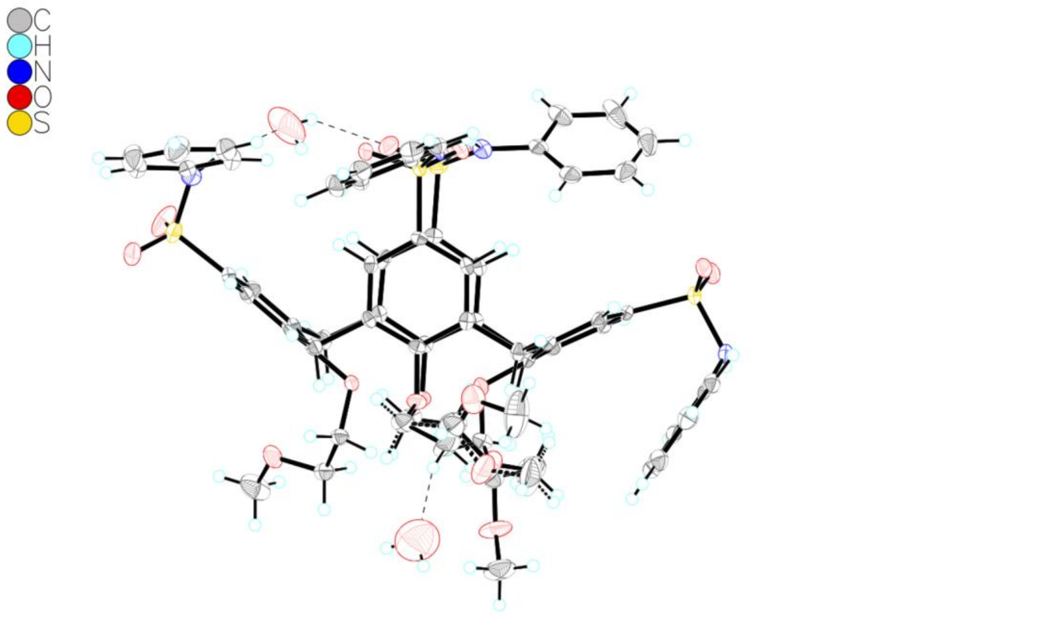

Figure S22. ORTEP representation of the structure of H-SA4$^{(0)}$ (side view). Thermal ellipsoids are shown at 50% probability level. Atoms are coloured based on elements, as displayed in the figure.

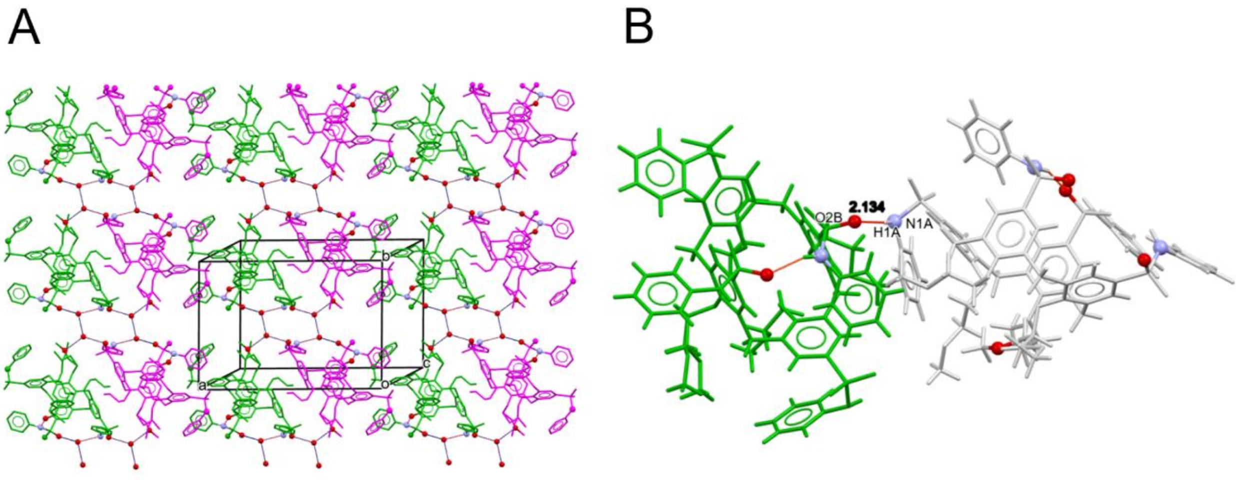

Figure S23. (A) Crystal packing of H-SA4$^{(0)}$ showing two distinct layers of calix[4]arene molecules. Molecules in each layer are colored differently to highlight their symmetry-related arrangement. Coordinated water molecules bridge adjacent molecules through hydrogen bonds (dashed lines). All hydrogen atoms are shown. **(B)** Stick representation of molecules showing an intermolecular hydrogen bond (N1A-H1A···O2B, 2.134 Å) between sulfonamides.

S4.2 Single crystal X-ray structure of Tf-SA4$^{(-4)}$

Tf-SA4$^{(-4)}$ was obtained after dissolution of Tf-SA4 in a 1:1 (v/v) water: acetonitrile mixture with 4 equivalents of NaOH and the solvent removed under reduced pressure. Then, the solid was redissolved in MeOH and colourless needles were grown by slow evaporation. The crystal of **Tf-SA4$^{(-4)}$** selected under the polarising microscope (Leica M165Z) was picked up on a Micro Mount (MiTeGen, USA) consisting of a thin polymer tip with a wicking aperture. The X-ray diffraction measurements were carried out on a Bruker D8Quest CCD diffractometer at 120 K using IµS Incoatec Microfocus Source with Mo-K*α* radiation (λ = 0.710723 Å). The single crystal, mounted on the goniometer using a cryo loop for intensity measurements, was coated with immersion oil type NVH and then quickly transferred to the cold nitrogen stream generated by an Oxford Cryostream 800 series. Symmetry-related absorption corrections using the program SADABS[45] were applied, and the data were corrected for Lorentz and polarisation effects using Bruker APEX4 software.[45] The structure was solved by program SHELXT[46] (with intrinsic phasing), and the full-matrix least-square refinements were carried out using SHELXL[47] through the Olex2[48] software suite. The non-hydrogen atoms were refined anisotropically, and H-atoms were refined isotropically and were fixed at stereochemically reasonable positions. A methanol molecule was identified on a crystallographic two-fold axis, positioned at the centre of the bowl-shaped cavity formed by the open conformation of the calixarene molecule. The methanol was refined with an occupancy of 0.75, indicating partial disorder or dynamic presence within the site. The difference Fourier map revealed several residual electron density peaks above the cavity. These were addressed using the solvent masking procedure available in the Olex2 software, which effectively accounted for diffuse or disordered electron density.

Based on the masked electron count within the void, this corresponds to four sodium (Na^+) ions per full calixarene molecule. Given that the asymmetric unit represents half of the calixarene molecule, this corresponds to two Na^+ ions per asymmetric unit. Crystallographic data supporting this interpretation are summarised in Table S2.

	**Tf-SA4(-4)**
**Crystal data**	
**Chemical formula**	$C_{68}H_{64}F_{12}N_4NaO_{24}S_8·2[Na_2]$
***M*r**	1920.66
**Crystal system, space group**	Monoclinic, *I*12/*c*1
**Temperature (K)**	120
***a*, *b*, *c* (Å)**	20.840 (3), 19.926 (2), 22.459 (3)
**β (°)**	99.284 (10)
***V* (Å^3)**	9204 (2)
Z	4
**Radiation type**	Mo *K*α
**μ (mm^{-1})**	0.31
**Crystal size (mm)**	0.024×0.032 ×0.16
**Data collection**	
**Diffractometer**	Bruker D8 Quest
**Absorption correction**	Multi-scan<br>*SADABS* (Bruker,2021) was used for absorption correction. wR2(int) was 0.1664 before and 0.1156 after correction. The Ratio of minimum to maximum transmission is 0.4998. The λ/2 correction factor is Not present.
**T_{min}, T_{max}**	0.372, 0.745
**No. of measured, independent and observed [*I* > 2σ(*I*)] reflections**	154779, 6722, 5246
**R_{int}**	0.258
**$θ_{max}$ (°)**	23.5
**$(\sin θ/λ)_{max}$ (Å^{-1})**	0.561
**Refinement**	
**$R[F^2 > 2σ(F^2)]$, $wR(F^2)$, *S***	0.117, 0.343, 1.33
**No. of reflections**	6722
**No. of parameters**	544
**No. of restraints**	62
**H-atom treatment**	H-atom parameters constrained
**$Δρ_{max}$, $Δρ_{min}$ (e Å^{-3})**	1.36, −1.04

Table S2 Crystallographic details of H-SA4(0).

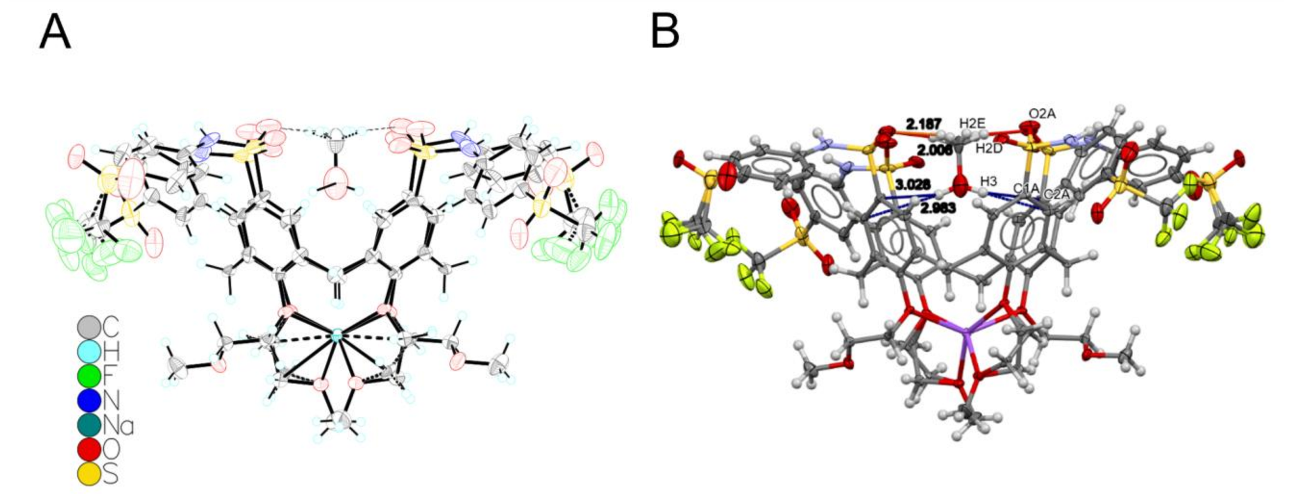

Figure S24. (A) ORTEP representation of the structure of Tf-SA4$^{(-4)}$ (side view). Thermal ellipsoids are shown at 50% probability level. Atoms are coloured based on elements, as displayed in the figure. A methanol molecule is included in the cavity. An adventitious sodium ion is coordinated with the oxygens from the ethers, other lattice sodium ions are omitted due to diffuse electron density. **(B)** Detailed view of the compound showing the included MeOH molecule. C–H···O and O–H···π interactions are indicated by dashed lines, with involved atoms labeled and interaction distances measured in Å.

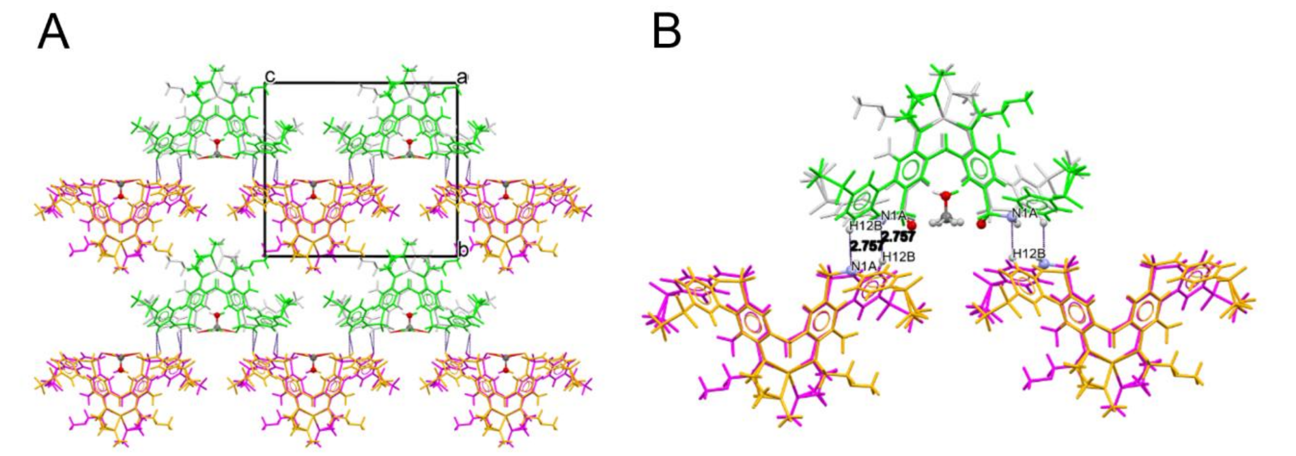

Figure S25. (A) Crystal packing of Tf-SA4$^{(-4)}$ (side view). Calixarene molecules including methanol are arranged in bilayers with the host "heads" facing each other, connected by C–H···N contacts (dashed lines). Molecules in each layer are colored differently to highlight symmetry-related sets. **(B)** Zoomed-in view highlighting short Calixarene···Calixarene contacts, with C–H···N interactions indicated by dashed lines and involved atoms labelled.

S5. 2-DASPI and 4-DASPI emission spectra from the semi-quantitative fluorescence assay

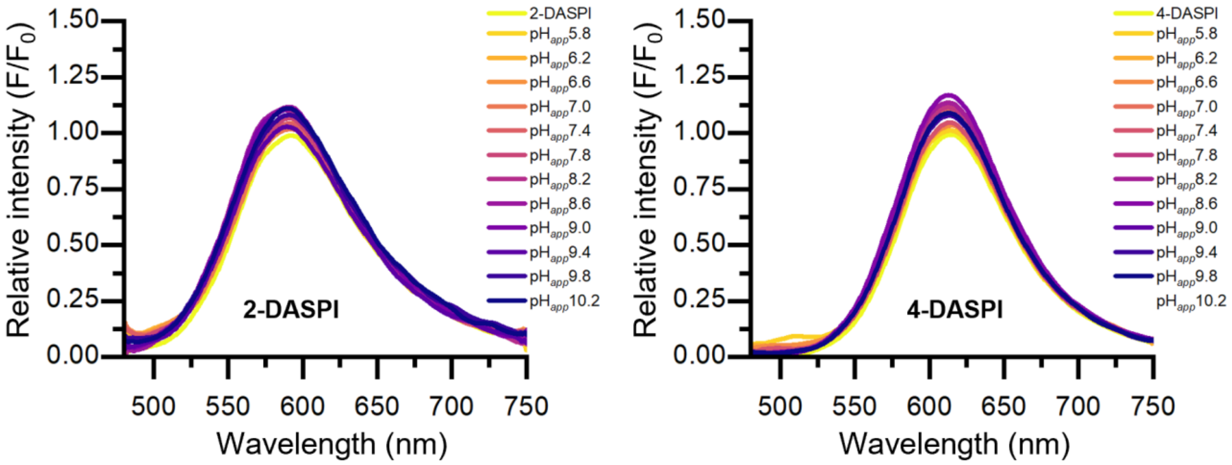

Figure S26 Relative emission spectra of **2-DASPI** (left panel, 2 µM, λ_{ex} 450 nm, λ_{em} 590 nm) and **4-DASPI** (right panel, 2 µM, λ_{ex} 460 nm, λ_{em} 613 nm) in absence and presence of **Tf-SA4m** (0.1 mM) in 1:1 (v/v) water:acetonitrile using 12 buffered (10 mM) solutions from pH_{app} 5.8 to 10.2.

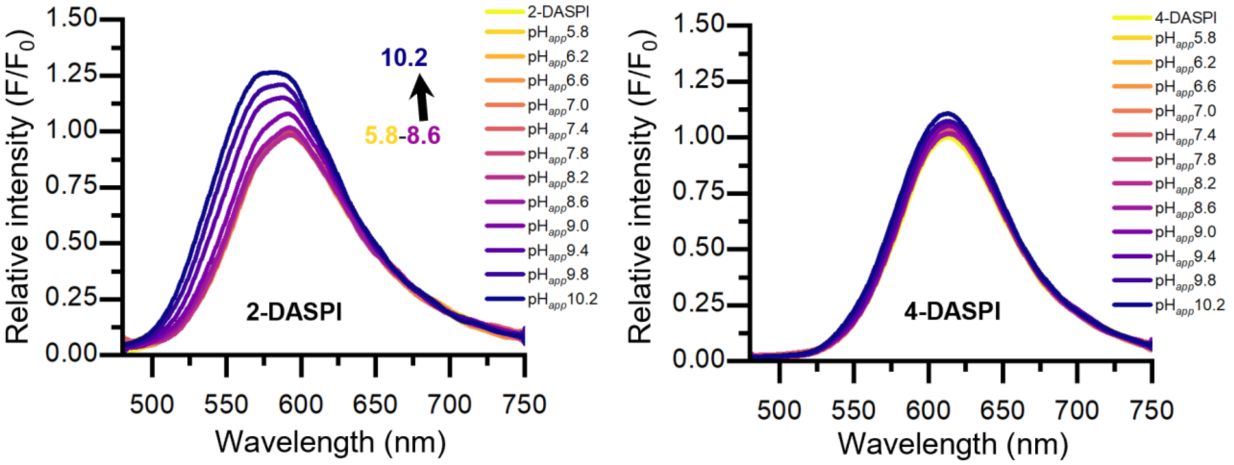

Figure S27 Relative emission spectra of **2-DASPI** (left panel, 2 µM, λ_{ex} 450 nm, λ_{em} 590 nm) and **4-DASPI** (right panel, 2 µM, λ_{ex} 460 nm, λ_{em} 613 nm) in absence and presence of **H-SA4** (0.1 mM) in 1:1 (v/v) water:acetonitrile using 12 buffered (10 mM) solutions from pH_{app} 5.8 to 10.2.

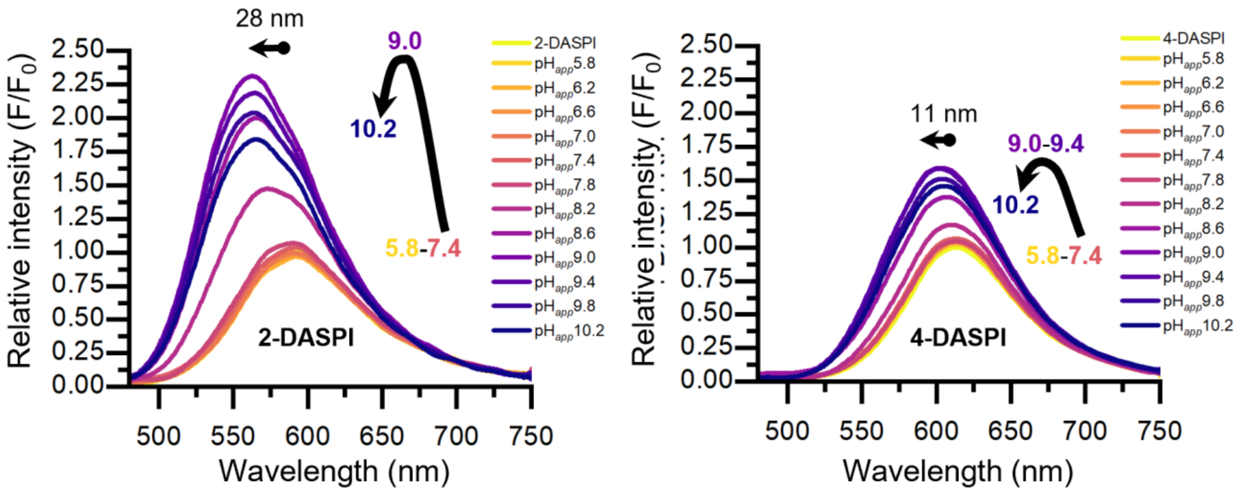

Figure S28 Relative emission spectra of **2-DASPI** (left panel, 2 µM, λ_{ex} 450 nm, λ_{em} 590 nm) and **4-DASPI** (right panel, 2 µM, λ_{ex} 460 nm, λ_{em} 613 nm) in absence and presence of **CN-SA4** (0.1 mM) in 1:1 (v/v) water:acetonitrile using 12 buffered (10 mM) solutions from pH$_{app}$ 5.8 to 10.2.

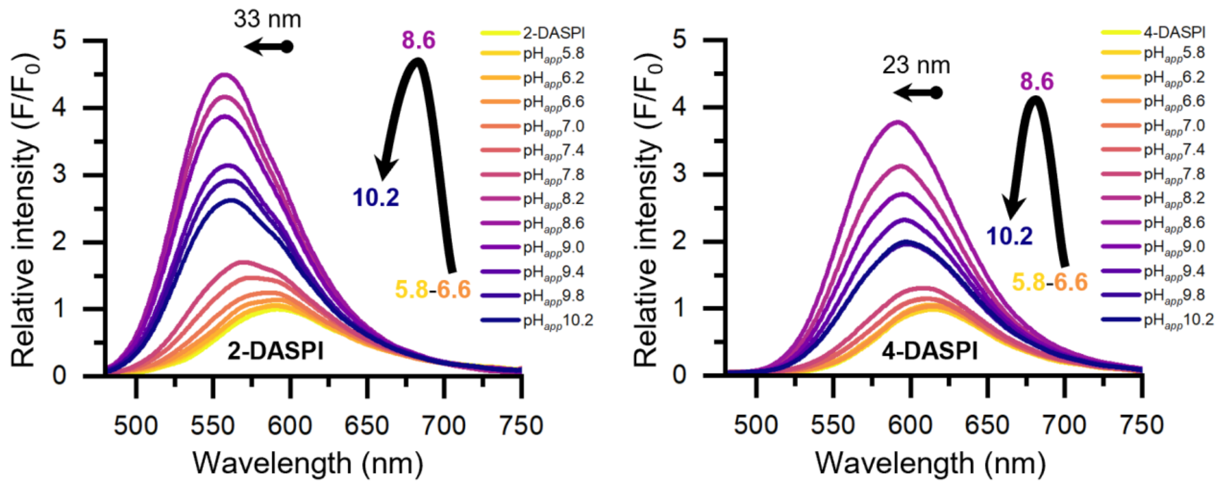

Figure S29 Relative emission spectra of **2-DASPI** (left panel, 2 µM, λ_{ex} 450 nm, λ_{em} 590 nm) and **4-DASPI** (right panel, 2 µM, λ_{ex} 460 nm, λ_{em} 613 nm) in absence and presence of **Tf-SA4** (0.1 mM) in 1:1 (v/v) water:acetonitrile using 12 buffered (10 mM) solutions from pH$_{app}$ 5.8 to 10.2.

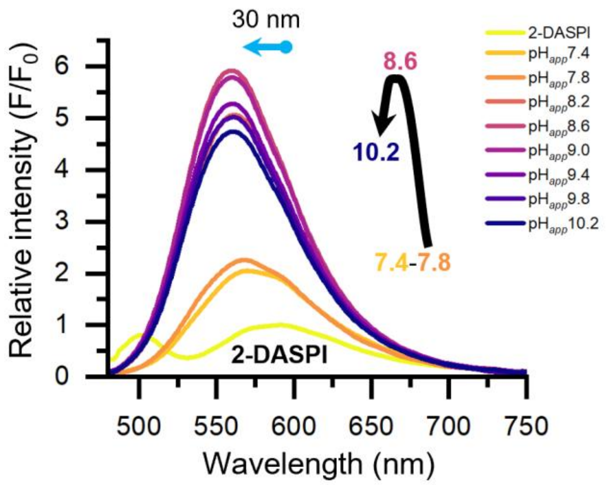

Figure S30 Relative emission spectra of **2-DASPI** (2 µM, λ_{ex} 450 nm, λ_{em} 590 nm) in absence and presence of **Tf-SA4** (0.1 mM) in 7:3 (v/v) water:acetonitrile using 12 buffered (10 mM) solutions from pH$_{app}$ 5.8 to 10.2.

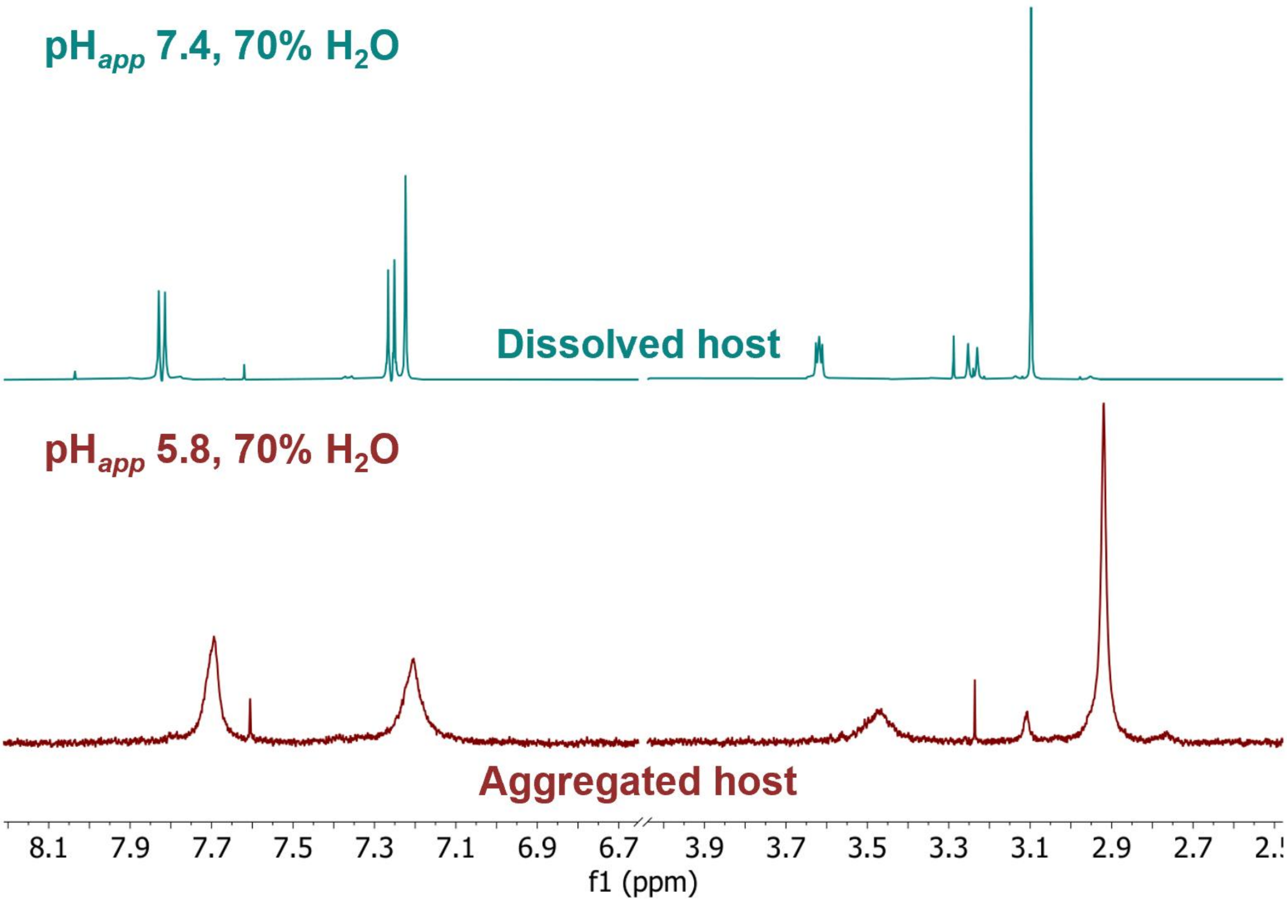

Figure S31 Partial ^{1}H NMR spectra of **Tf-SA4** at pH$_{app}$ 7.4 (upper spectrum) and pH$_{app}$ 5.8 (lower spectrum) using 10 mM sodium phosphate buffer in a 7:3 (v/v) H_2O:CD_3CN mixture. The fluorescence assay at 70% water starts at pH$_{app}$ 7.4 because at pH$_{app}$ 5.8, Tf-SA4 is forming aggregates (broad signals). However, it is soluble at pH$_{app}$ 7.4, due to increased ionization.

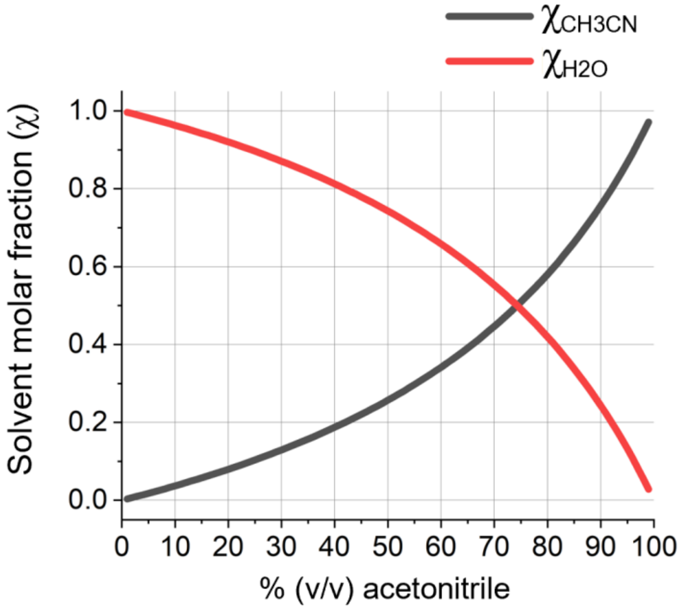

Figure S32 Molar fraction of water and acetonitrile in binary mixtures, as a function of acetonitrile content.

S6. NMR titrations and statistical analysis

Titrations were performed using **Tf-SA4** (1 mM) in a 1:1 (v/v) mixture of H_2O and CD_3CN, buffered at pH_{app} 7.4, 7.8, 8.2, or 8.6 with 50 mM of the corresponding buffer. A total of 800 µL of this solution was prepared for each titration. To generate the titrant solution, 2.2 mg of **2-DASPI** were accurately weighed and dissolved in 240 µL of the same buffered **Tf-SA4** solution, ensuring that the host concentration remained constant during titration.

For each titration, 500 µL of the **Tf-SA4** solution were transferred into a 5 mm NMR tube. Using a positive displacement micropipette (Gilson, 10 µL and 100 µL models), 15 successive aliquots of the titrant solution were added to the NMR tube. After each addition, the tube was gently flipped upside down and back several times to allow gravity-driven mixing using a Teflon insert NMR cap. The total volume in the NMR tube after the final addition was 704 µL, corresponding to 6.96 equivalents of 2-DASPI. ^{1}H NMR spectra (600 MHz) were recorded after each addition and at the start of the experiment. Each titration was repeated in triplicate for each pH_{app} condition

The chemical shifts of protons Hb and Hc (Figure S28), which report on the calix[4]arene cavity environment, were used for binding model fitting. These values from each titration step were uploaded to the supramolecular.org (Bindfit)[27] web tool for non-linear regression analysis. All available binding models were tested to assess binding stoichiometry. For the 1:2 and 2:1 stoichiometries, multiple submodels ("flavours")[28] were evaluated; a brief summary of their differences is provided in Table S1.

**Binding stoichiometry**[a]	**Binding "flavour"**	**Number of fitted parameters**[b]	**Relationship between**	
			**K_1 and K_2**	**$\delta_{\Delta HG}$ and $\delta_{\Delta H\cdot 2G}$**[a]
1:1	N/A	3	N/A	N/A
1:2	Full	6	$K_1 \neq K_2$	$\delta_{\Delta HG} \neq \delta_{\Delta H\cdot 2G}$
1:2	Non-cooperative	5	$K_1 = 4K_2$	$\delta_{\Delta HG} \neq \delta_{\Delta H\cdot 2G}$
1:2	Additive	4	$K_1 \neq K_2$	$\delta_{\Delta HG} = \delta_{\Delta H\cdot 2G}$
1:2	Statistical	3	$K_1 = 4K_2$	$\delta_{\Delta HG} = \delta_{\Delta H\cdot 2G}$

Table S3. Key features and differences of the binding models or "flavours" used by supramolecular.org (Bindfit). Information about flavours obtained from Howe (2014),[28] see their Supporting Information for more detailed explanations. [a]For 2:1 stoichiometries, the variable labels are swapped by defining the guest species as [H] and the host as [G] in the binding fitting process. [b]For this work, 2 proton resonances are used in the global fitting process.

The quality of each fit was assessed using the covariance of the fit (cov_{fit})[29] and the Bayesian Information Criterion (BIC)[30] for each model at each pH_{app} value. For comparative analysis, ΔBIC values were also calculated as the difference between the BIC of a given model/flavour and that of the best-fitting model (the one with the lowest BIC) within the same titration iteration. A ΔBIC > 10 indicates strong evidence against the compared model in favour of the best one.

S6.1 NMR titration of Tf-SA4 and 2-DASPI at pH$_{app}$ 7.4

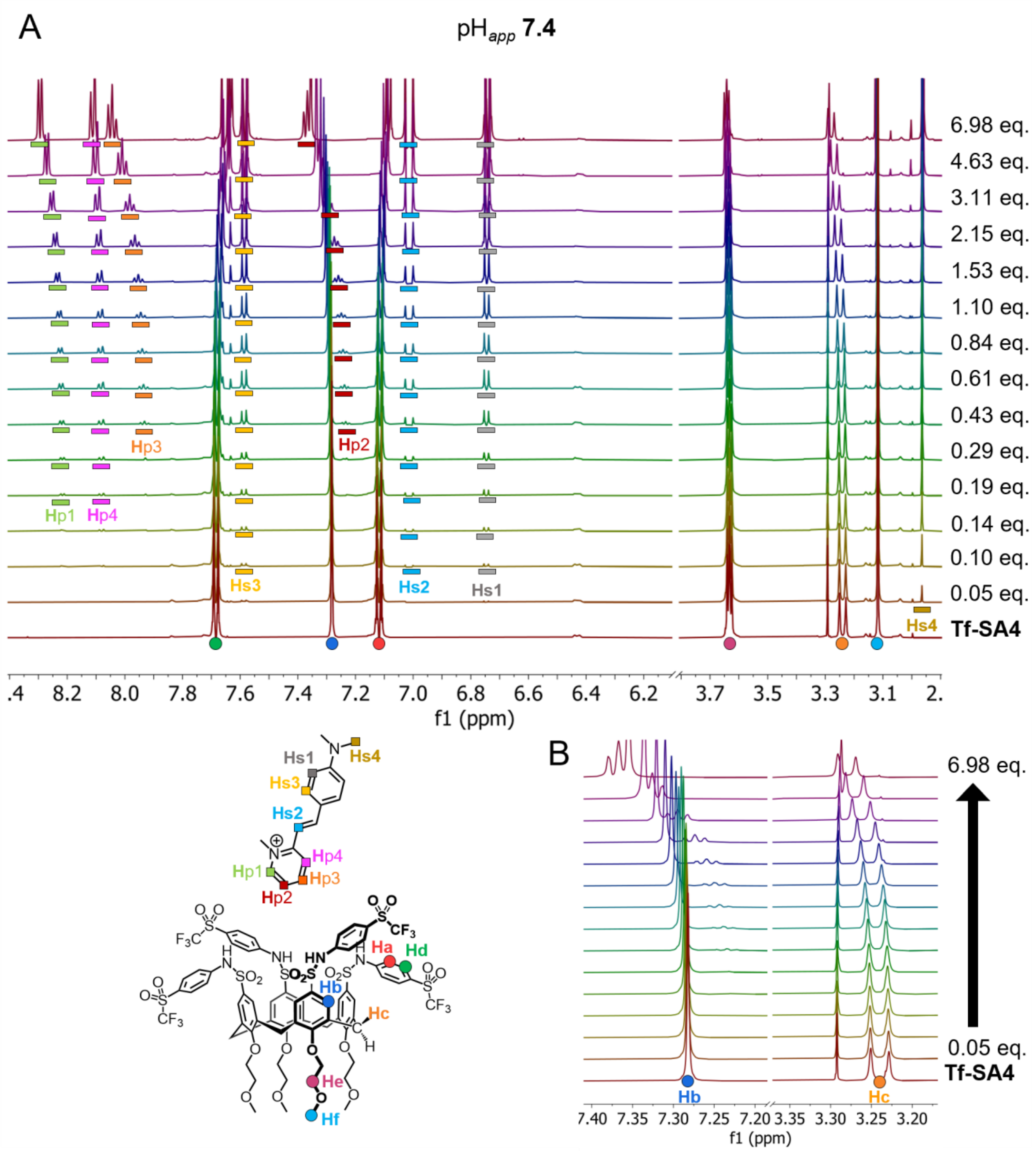

Figure S33. **A)** Representative ^{1}H NMR titration (600 MHz, 298 K) from one of three independent replicates at pH$_{app}$ 7.4 in 1:1 (v/v) H_2O:CD_3CN, 50 mM buffer of **Tf-SA4** with **2-DASPI** (guest equivalents added to host (constant concentration, 1 mM)). Host and guest protons are assigned with coloured shapes in their respective structures: **Tf-SA4** signals as (●) and **2-DASPI** signals as (■). **2-DASPI** resonances are displayed with bar below with their assigned color. **B) Tf-SA4** cavity signals used for the fitting process.

S6.2 NMR titration of Tf-SA4 and 2-DASPI at pH$_{app}$ 7.8

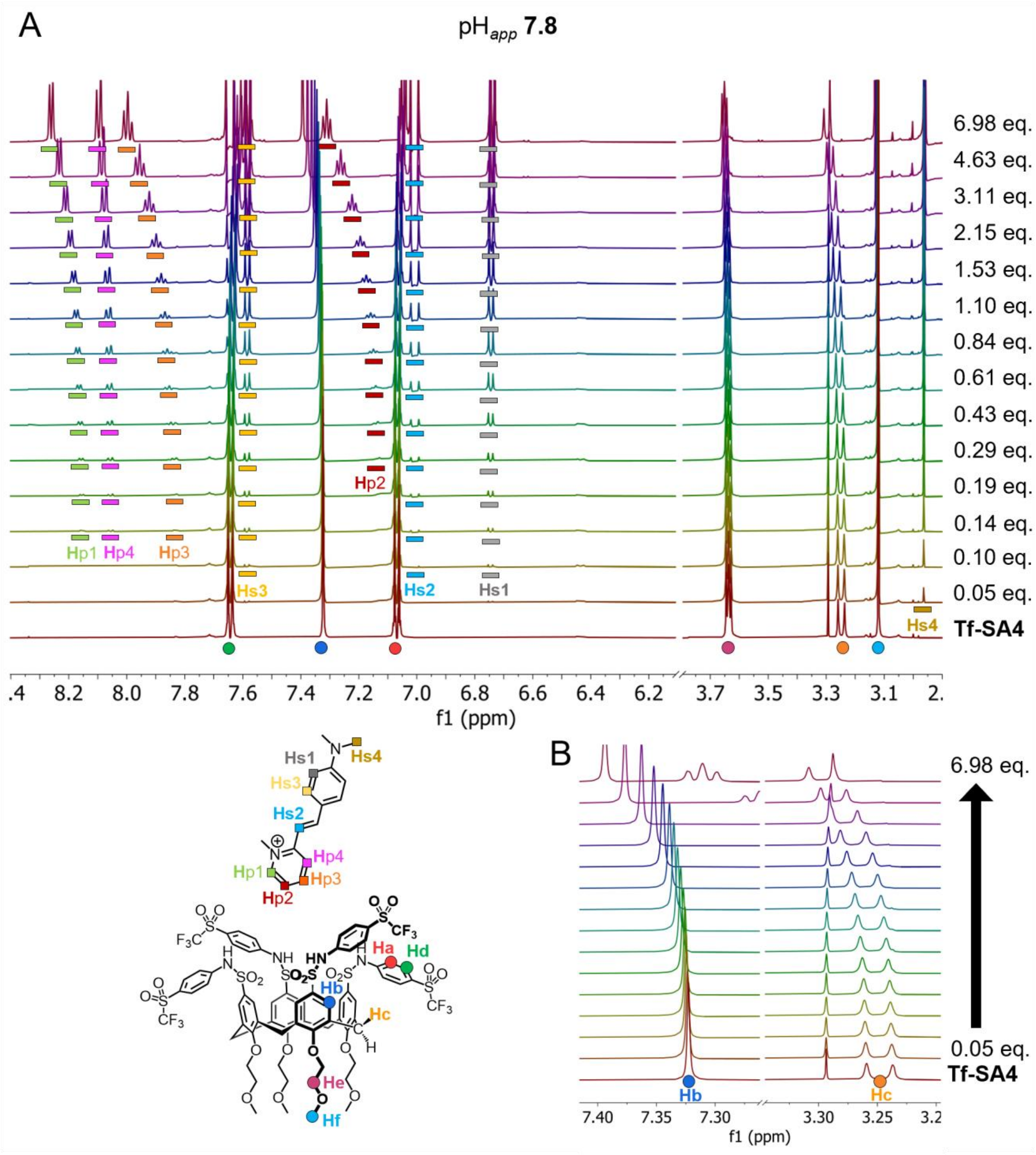

Figure S34. **A)** Representative ^{1}H NMR titration (600 MHz, 298 K) from one of three independent replicates at pH$_{app}$ 7.8 in 1:1 (v/v) H_2O:CD_3CN, 50 mM buffer of **Tf-SA4** with **2-DASPI** (guest equivalents added to host (constant concentration, 1 mM)). Host and guest protons are assigned with coloured shapes in their respective structures: **Tf-SA4** signals as (●) and **2-DASPI** signals as (■). **2-DASPI** resonances are displayed with bar below with their assigned color. **B) Tf-SA4** cavity signals used for the fitting process.

S6.3 NMR titration of Tf-SA4 and 2-DASPI at pH$_{app}$ 8.2

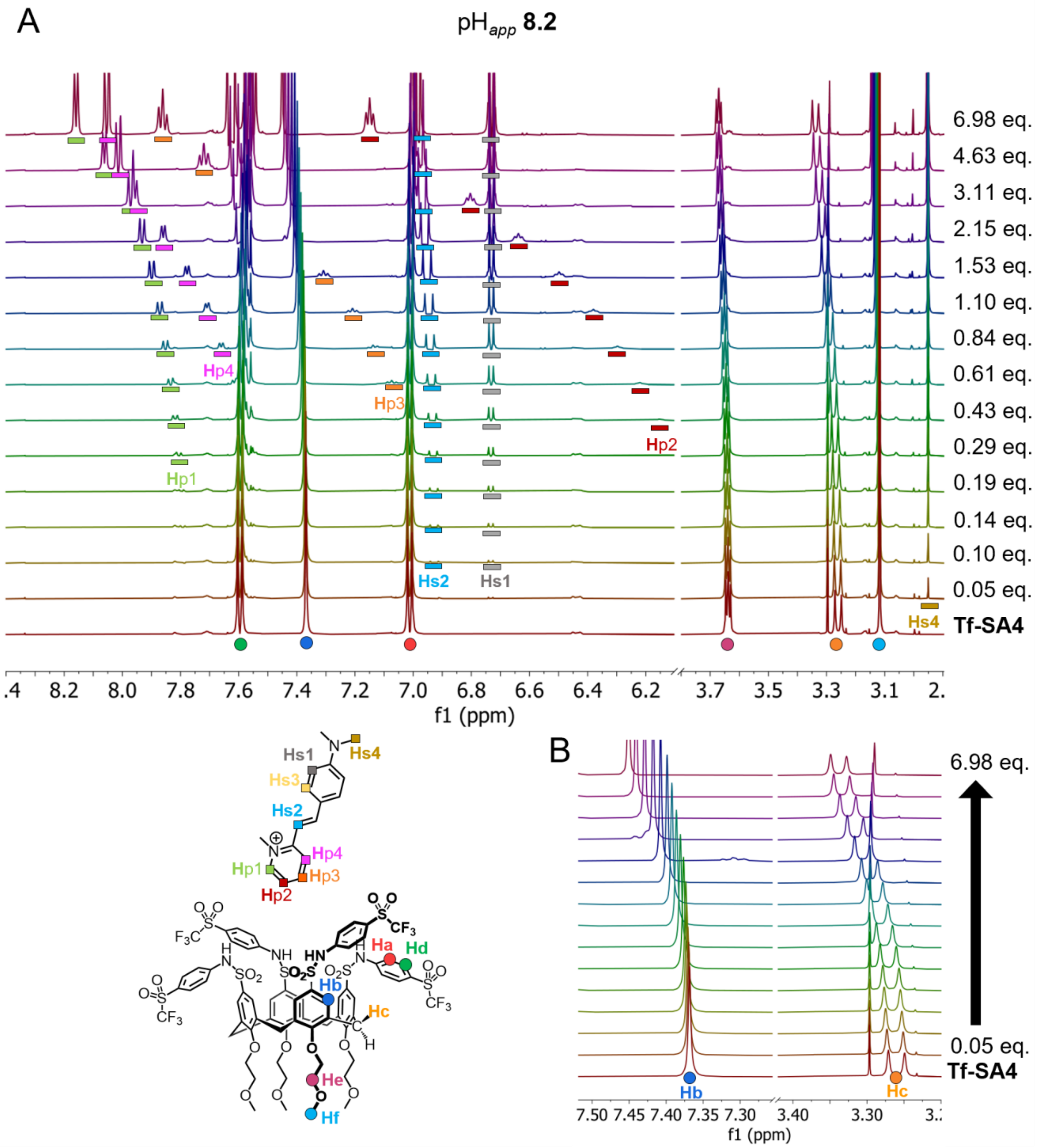

Figure S35. A) Representative ^{1}H NMR titration (600 MHz, 298 K) from one of three independent replicates at pH$_{app}$ 8.2 in 1:1 (v/v) H_2O:CD_3CN, 50 mM buffer of **Tf-SA4** with **2-DASPI** (guest equivalents added to host (constant concentration, 1 mM)). Host and guest protons are assigned with coloured shapes in their respective structures: **Tf-SA4** signals as (●) and 2-DASPI signals as (■). 2-DASPI resonances are displayed with bar below with their assigned color. **B) Tf-SA4** cavity signals used for the fitting process.

S6.4 NMR titration of Tf-SA4 and 2-DASPI at pH$_{app}$ 8.6

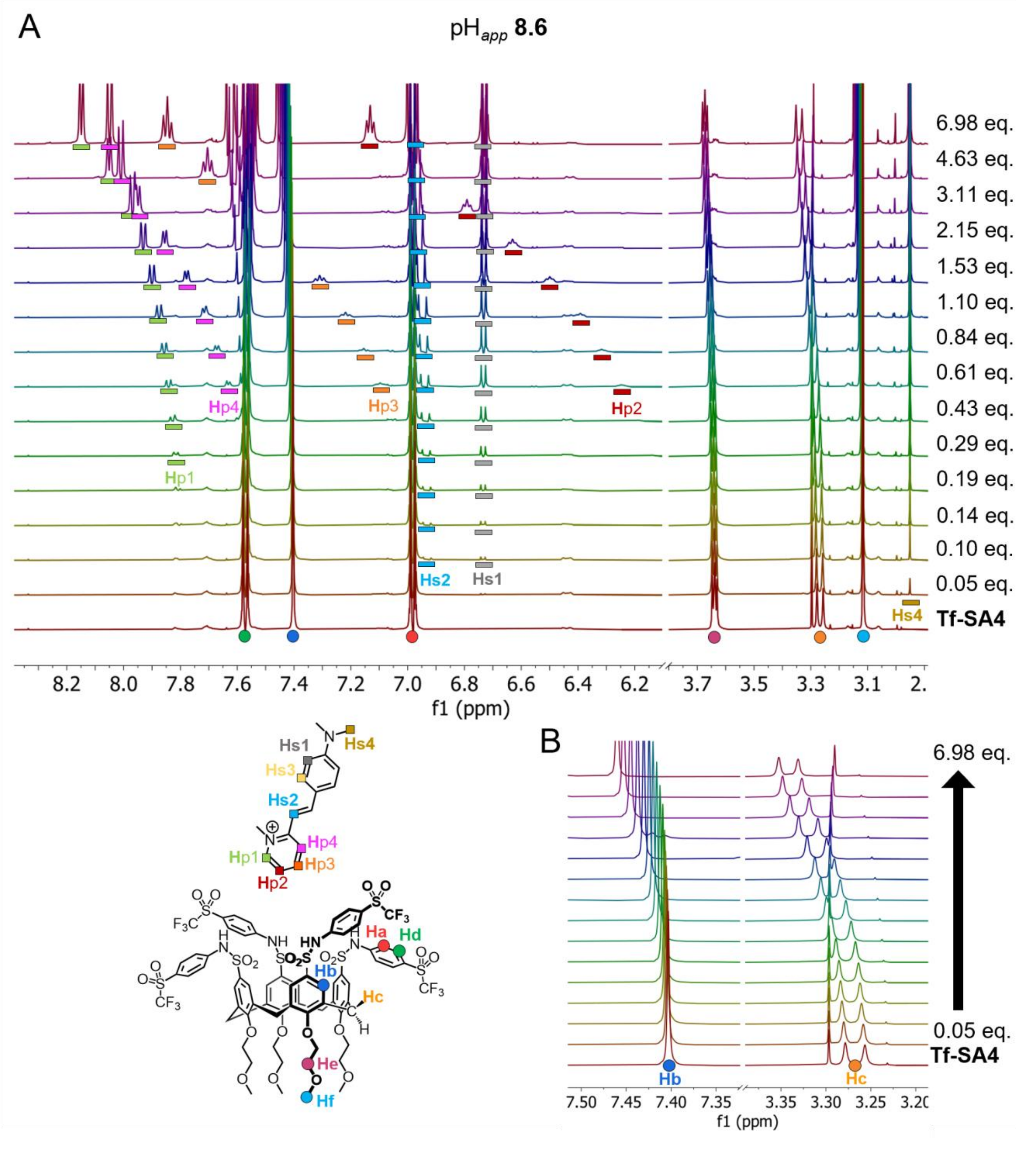

Figure S36. **A)** Representative ^{1}H NMR titration (600 MHz, 298 K) from one of three independent replicates at pH$_{app}$ 8.6 in 1:1 (v/v) H_2O:CD_3CN, 50 mM buffer of **Tf-SA4** with **2-DASPI** (guest equivalents added to host (constant concentration, 1 mM)). Host and guest protons are assigned with coloured shapes in their respective structures: **Tf-SA4** signals as (●) and **2-DASPI** signals as (■). **2-DASPI** resonances are displayed with bar below with their assigned color. **B) Tf-SA4** cavity signals used for the fitting process.

S6.5 Binding isotherms of Tf-SA4 protons Hb and Hc

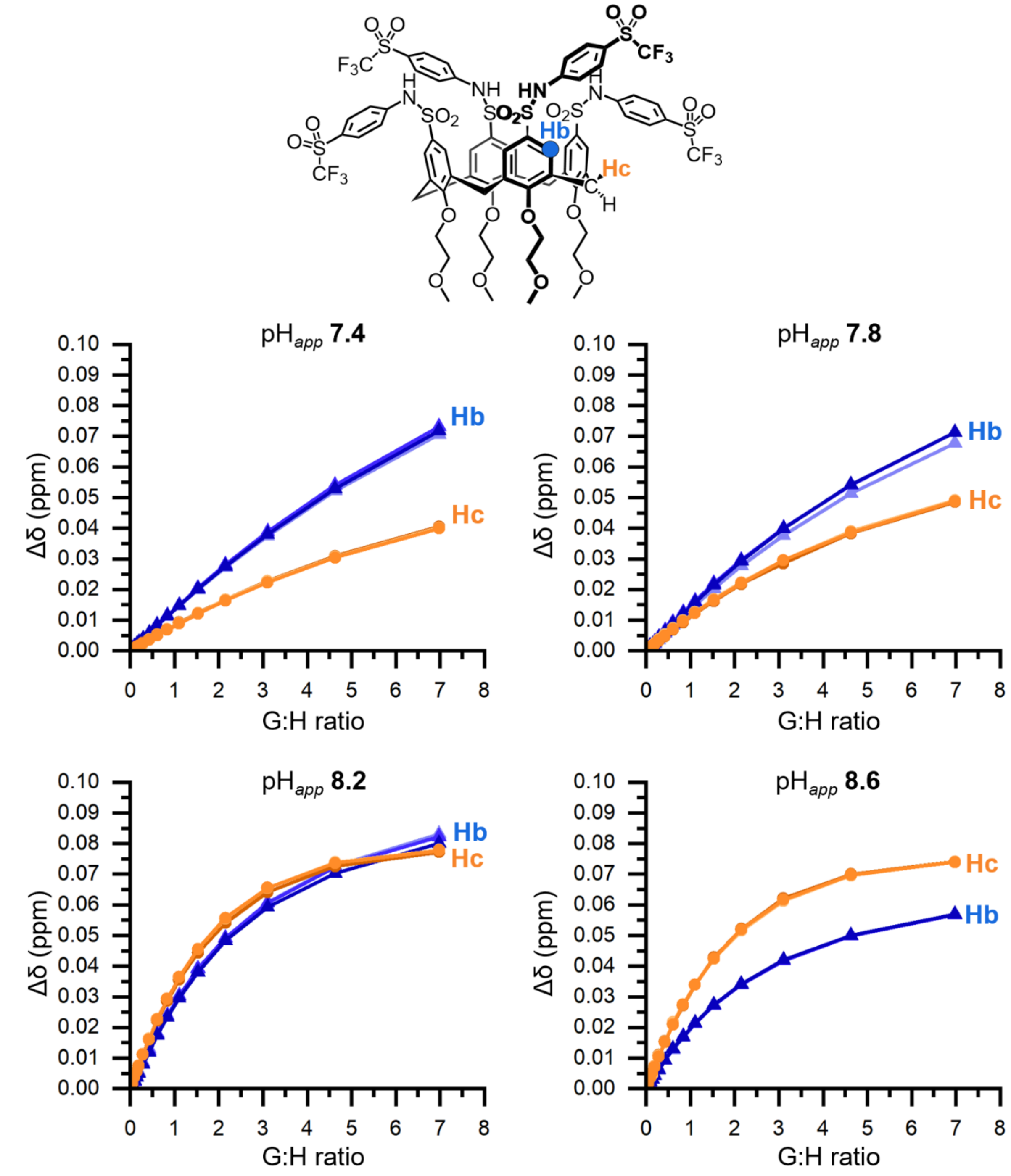

Figure S37 ^{1}H NMR (600 MHz, 298K) titration isotherms of selected **Tf-SA4** (1 mM) protons Hb and Hc upon addition of 2-DASPI in 1:1 (v/v) $H_2O:CD_3CN$ using buffered (50 mM) solutions from pH$_{app}$ 7.4, 7.8, 8.2 and 8.6. Experiments made in triplicate with 15 points each, and replicates are displayed as symbols and lines of similar colours for each proton.

S6.1 Analysis for experiment at pH$_{app}$ 7.4

Model (C:D)	*n*	Flavour	K_1	K_2	cov_{fit}	BIC	ΔBIC
1:1	1		68.22		2.94E-04	-392.70	82.98
	2		65.96		3.72E-04	-383.65	69.13
	3		65.23		2.79E-04	-393.77	94.87
	Mean(SD)		*66.47 (1.56)*		*3.15E-04 (5.02E-05)*	*-390.04 (5.56)*	
1:2	1	Full	54.04	30.23	1.32E-05	-475.68	0.00
	2		135.22	77.65	2.57E-05	-451.77	1.01
	3		41.78	17.61	8.13E-06	-488.64	0.00
	Mean(SD)		*77.01 (50.78)*	*41.83 (31.66)*	*1.57E-05 (9.02E-06)*	*-472.03 (18.70)*	
1:2	1	Non-Coop	246.85	61.71	2.02E-05	-466.19	9.49
	2		306.92	76.73	2.79E-05	-452.78	0.00
	3		237.13	59.28	1.57E-05	-472.34	16.30
	Mean(SD)		*263.63 (37.80)*	*65.91 (9.45)*	*2.13E-05 (6.20E-06)*	*-463.77 (10.00)*	
1:2	1	Additive	187.54	61.73	2.72E-04	-391.38	84.30
	2		201.63	78.68	3.16E-04	-385.39	67.39
	3		179.93	59.59	2.59E-04	-392.40	96.24
	Mean(SD)		*189.70 (11.01)*	*66.67 (10.46)*	*2.82E-04 (3.01E-05)*	*-389.72 (3.79)*	
1:2	1	Statistical	154.96	38.74	2.89E-04	-393.26	82.42
	2		149.35	37.34	3.64E-04	-384.37	68.41
	3		147.42	36.86	2.74E-04	-394.28	94.36
	Mean(SD)		*150.58 (3.92)*	*37.64 (0.98)*	*3.09E-04 (4.83E-05)*	*-390.64 (5.45)*	
2:1	1	Full	58.61	-193.51			
	2		127.09	-216.01			
	3		45.45	-177.65			
	Mean(SD)		77.05 (43.83)	-195.72 (19.28)			
2:1	1	Non-Coop	NF	NF			
	2		NF	NF			
	3		NF	NF			
	Mean(SD)						
2:1	1	Additive	118.93	-71.20			
	2		143.04	-91.19			
	3		115.10	-72.05			
	Mean(SD)		125.69 (15.15)	-78.15 (11.30)			
2:1	1	Statistical	NF	NF			
	2		NF	NF			
	3		NF	NF			
	Mean(SD)						

Table S4 Isotherms fitting results from supramolecular.org (Bindfit) for the NMR titration of **Tf-SA4** (1 mM) and **2-DASPI** in 1:1 (v/v) H_2O:CD_3CN mixture at pH$_{app}$ 7.4 (sodium phosphate buffer 50 mM). NF: no fitting possible. ΔBIC values were calculated from the lowest BIC value for each *n* (comparison for each sample).

Model	Flavour	*n*	Supramolecular.org (Bindfit) URL link
1:1		1	http://app.supramolecular.org/bindfit/view/922416e1-25d3-4112-ac9c-5b7c6c0ac6f1
		2	http://app.supramolecular.org/bindfit/view/5e1b0b90-5bb5-4332-b17d-ea7f8edb08a2
		3	http://app.supramolecular.org/bindfit/view/fef3aa3d-36f7-4d24-bc5e-6d0bf73c136c
1:2	Full	1	http://app.supramolecular.org/bindfit/view/451b5858-a7a2-4c6a-b95f-ebd0ee9814ac
	Full	2	http://app.supramolecular.org/bindfit/view/0cf4e5d4-7ad1-4672-bc55-5a236b056c25
	Full	3	http://app.supramolecular.org/bindfit/view/103a7f0d-50fe-41da-b3b2-50c414f2c98c
	Non-Coop	1	http://app.supramolecular.org/bindfit/view/45e61e03-29e4-4593-bfd8-5d0118654aca
	Non-Coop	2	http://app.supramolecular.org/bindfit/view/f406da3c-e587-47fe-acb9-8421fbf0f0b1
	Non-Coop	3	http://app.supramolecular.org/bindfit/view/38159cfa-75f4-484c-a771-fc37eed75bc4
	Additive	1	http://app.supramolecular.org/bindfit/view/86795df4-8590-437a-b68e-3d23cbb896fb
	Additive	2	http://app.supramolecular.org/bindfit/view/fe7c7574-9d79-46c1-9237-a40bbfd409fc
	Additive	3	http://app.supramolecular.org/bindfit/view/8b68a0ef-8a30-49a9-9f18-c25b800bdf84
	Statistical	1	http://app.supramolecular.org/bindfit/view/6c43a631-4948-49ee-b1aa-0ba4a3900de1
	Statistical	2	http://app.supramolecular.org/bindfit/view/35326db9-cd16-4a96-ba12-f42bfbaa0a0c
	Statistical	3	http://app.supramolecular.org/bindfit/view/41df9213-112a-40fe-874f-2a0dc851b03d

Table S5 The supramolecular.org (Bindfit) permanent URL links for the fitting results for each iteration and each binding model for the NMR titration of **Tf-SA4** (1 mM) and **2-DASPI** in 1:1 (v/v) H_2O:CD_3CN mixture at pH$_{app}$ 7.4 (sodium phosphate buffer 50 mM).

S6.2 Analysis for experiment at pH$_{app}$ 7.8

Model (C:D)	*n*	Flavour	K$_1$	K$_2$	*cov*$_{fit}$	BIC	ΔBIC
1:1	1		95.34		8.60E-04	-357.97	58.68
	2		107.75		7.05E-04	-362.62	107.08
	3		99.04		8.60E-04	-356.31	86.57
	Mean(SD)		*100.71 (6.37)*		*8.08E-04 (8.96E-05)*	*-358.97 (3.27)*	
1:2	1	Full	1192.70	106.87	8.81E-05	-414.20	2.45
	2		227.20	62.41	1.54E-05	-466.91	2.79
	3		196.94	105.94	3.19E-05	-442.88	0.00
	Mean(SD)		*538.95 (566.37)*	*91.74 (25.40)*	*4.51E-05 (3.81E-05)*	*-441.33 (26.39)*	
1:2	1	Non-Coop	444.81	111.20	9.01E-05	-416.65	0.00
	2		265.55	66.39	1.58E-05	-469.70	0.00
	3		432.05	108.01	3.74E-05	-441.86	1.02
	Mean(SD)		*380.80 (100.02)*	*95.20 (25.00)*	*4.78E-05 (3.82E-05)*	*-442.74 (26.54)*	
1:2	1	Additive	286.99	111.61	7.65E-04	-358.93	57.72
	2		264.55	67.70	6.97E-04	-359.45	110.25
	3		293.55	110.06	7.60E-04	-356.90	85.98
	Mean(SD)		*281.70 (15.21)*	*96.46 (24.92)*	*7.40E-04 (3.79E-05)*	*-358.43 (1.35)*	
1:2	1	Statistical	226.97	56.74	8.34E-04	-358.98	57.67
	2		260.96	65.24	6.97E-04	-362.85	106.86
	3		237.17	59.29	8.31E-04	-357.38	85.50
	Mean(SD)		*241.70 (17.44)*	*60.43 (4.36)*	*7.88E-04 (7.82E-05)*	*-359.73 (2.81)*	
2:1	1	Full	198.83	747.68	7.04E-05	-422.88	-6.23
	2		117.72	-71.74			
	3		165.84	-209.39			
	Mean(SD)		*160.80 (40.79)*	*155.52 (517.43)*	*2.35E-05 (4.06E-05)*		
2:1	1	Non-Coop	NF	NF			
	2		NF	NF			
	3		NF	NF			
	Mean(SD)						
2:1	1	Additive	118.93	-71.20			
	2		143.04	-91.19			
	3		115.10	-72.05			
	Mean(SD)		*125.69 (15.15)*	*-78.15 (11.30)*			
2:1	1	Statistical	NF	NF			
	2		NF	NF			
	3		NF	NF			
	Mean(SD)						

Table S6 Isotherms fitting results from supramolecular.org (Bindfit) for the NMR titration of **Tf-SA4** (1 mM) and **2-DASPI** in 1:1 (v/v) H_2O:CD_3CN mixture at pH$_{app}$ 7.8 (sodium phosphate buffer 50 mM). NF: no fitting possible. ΔBIC values were calculated from the lowest BIC value for each *n* (comparison for each sample).

Model	Flavour	*n*	Supramolecular.org (Bindfit) URL link
1:1		1	http://app.supramolecular.org/bindfit/view/1675df8b-8579-47e8-a22b-5f5becbc83c5
1:1		2	http://app.supramolecular.org/bindfit/view/7372c38a-badb-4b58-bc84-17ed9f05e5e8
1:1		3	http://app.supramolecular.org/bindfit/view/ba2e6a90-c7e6-46f0-a029-bf4e613e4624
1:2	Full	1	http://app.supramolecular.org/bindfit/view/6efe35e5-de1d-473f-b4a5-db9d7537cd0b
1:2	Full	2	http://app.supramolecular.org/bindfit/view/fb8efaaf-ffd9-435a-a618-900a0a3c868b
1:2	Full	3	http://app.supramolecular.org/bindfit/view/6da38d51-7735-4a91-a41c-0a516ddbc729
1:2	Non-Coop	1	http://app.supramolecular.org/bindfit/view/34deab66-84a1-47d7-ac94-c16055f977ab
1:2	Non-Coop	2	http://app.supramolecular.org/bindfit/view/d01a701f-0198-4bb7-afaa-e0b9331ca50e
1:2	Non-Coop	3	http://app.supramolecular.org/bindfit/view/3d045ef4-9314-4710-b67c-cf776ea4108e
1:2	Additive	1	http://app.supramolecular.org/bindfit/view/c904d736-f1f4-4403-b093-f4b5dabb7c84
1:2	Additive	2	http://app.supramolecular.org/bindfit/view/bb0ac317-e8b8-4255-b921-a267d255f13e
1:2	Additive	3	http://app.supramolecular.org/bindfit/view/df6e736b-143b-4390-8e2d-20fa0e74564c
1:2	Statistical	1	http://app.supramolecular.org/bindfit/view/611a6383-142a-46e1-bec6-6fbce47126c6
1:2	Statistical	2	http://app.supramolecular.org/bindfit/view/3b33d763-b8e9-4d72-bbbc-371123375fd9
1:2	Statistical	3	http://app.supramolecular.org/bindfit/view/900797f5-2169-474e-8ea6-f8c13dee095f
2:1	Full	1	http://app.supramolecular.org/bindfit/view/d047c3e1-9aae-44fa-83a2-f11ca1f5bba2

Table S7 The supramolecular.org (Bindfit) permanent URL links for the fitting results for each iteration and each binding model for the NMR titration of **Tf-SA4** (1 mM) and 2-**DASPI** in 1:1 (v/v) H_2O:CD_3CN mixture at pH$_{app}$ 7.8 (sodium phosphate buffer 50 mM).

S6.3 Analysis for experiment at pH$_{app}$ 8.2

Model (C:D)	*n*	Flavour	K$_1$	K$_2$	*cov*$_{fit}$	BIC	ΔBIC
1:1	1		592.80		4.52E-03	-264.97	111.94
	2		610.57		4.52E-03	-281.15	93.42
	3		620.78		4.92E-03	-281.87	94.73
	Mean(SD)		*608.05 (14.16)*		*4.65E-03 (2.33E-04)*	*-276.00 (9.56)*	
1:2	1	Full	831.61	245.90	7.65E-05	-376.90	0.00
	2		1028.41	373.45	2.01E-04	-374.58	0.00
	3		1079.88	419.51	1.96E-04	-376.60	0.00
	Mean(SD)		*979.97 (131.03)*	*346.29 (89.94)*	*1.58E-04 (7.04E-05)*	*-376.03 (1.27)*	
1:2	1	Non-Coop	1003.53	250.88	8.32E-05	-367.23	9.68
	2		1632.07	408.02	2.15E-04	-366.35	8.23
	3		1814.09	453.52	2.12E-04	-368.21	8.39
	Mean(SD)		*1483.23 (425.28)*	*370.81 (106.32)*	*1.70E-04 (7.54E-05)*	*-367.26 (0.93)*	
1:2	1	Additive	438.31	-12.82			
	2		402.96	-16.68			
	3		-16.68	-17.81			
	Mean(SD)		*274.86 (253.10)*	*-15.77 (2.62)*			
1:2	1	Statistical	2700.57	675.14	5.03E-03	-261.76	115.14
	2		2918.09	729.52	4.96E-03	-281.47	93.10
	3		3032.12	758.03	4.75E-03	-283.54	93.06
	Mean(SD)		*2883.59 (168.45)*	*720.90 (42.11)*	*4.91E-03 (1.46E-04)*	*-275.59 (12.02)*	
2:1	1	Full	366.82	-172.62			
	2		448.90	-237.92			
	3		459.20	-256.68			
	Mean(SD)		424.97 (50.62)	-222.41 (44.13)			
2:1	1	Non-Coop	904.04	226.01	5.05E-04	-319.69	57.22
	2		1113.35	278.34	5.84E-04	-338.88	35.70
	3		1177.59	294.40	6.37E-04	-337.10	39.50
	Mean(SD)		*1064.99 (143.04)*	*266.25 (35.76)*	*5.75E-04 (6.66E-05)*	*-331.89 (10.60)*	
2:1	1	Additive	788.45	-47.22			
	2		932.51	-67.53			
	3		985.33	-73.11			
	Mean(SD)		*902.10 (101.90)*	*-62.62 (13.63)*			
2:1	1	Statistical	NF	NF			
	2		NF	NF			
	3		NF	NF			
	Mean(SD)						

Table S8 Isotherms fitting results from supramolecular.org (Bindfit) for the NMR titration of **Tf-SA4** (1 mM) and **2-DASPI** in 1:1 (v/v) H_2O:CD_3CN mixture at pH$_{app}$ 8.2 (sodium borate buffer 50 mM). NF: no fitting possible. ΔBIC values were calculated from the lowest BIC value for each *n* (comparison for each sample).

Model	Flavour	*n*	Supramolecular.org (Bindfit) URL link
1:1		1	http://app.supramolecular.org/bindfit/view/540dc277-cc19-472e-8d7f-1a25bad43800
1:1		2	http://app.supramolecular.org/bindfit/view/2b856e06-7ca0-4b0e-a355-d01a29c219c0
1:1		3	http://app.supramolecular.org/bindfit/view/205a9724-05bb-4c06-91af-c0426f3b211d
1:2	Full	1	http://app.supramolecular.org/bindfit/view/5ff859bc-38d5-4128-bc23-96a453b1d762
1:2	Full	2	http://app.supramolecular.org/bindfit/view/92752179-be46-40a1-9cc6-a12a48323e4a
1:2	Full	3	http://app.supramolecular.org/bindfit/view/83f30dee-4a3f-4633-899a-b35f6b04c7fb
1:2	Non-Coop	1	http://app.supramolecular.org/bindfit/view/0a4c70f3-57e2-47b1-a335-a1faca157cff
1:2	Non-Coop	2	http://app.supramolecular.org/bindfit/view/b3911a59-3239-4e45-a46d-d51399f11dbc
1:2	Non-Coop	3	http://app.supramolecular.org/bindfit/view/0041dbe8-3815-43d6-b0f2-eea817b6d06e
1:2	Statistical	1	http://app.supramolecular.org/bindfit/view/acd9fafa-f4d8-4f66-97e2-af3115f70a80
1:2	Statistical	2	http://app.supramolecular.org/bindfit/view/7abe19c5-0286-4103-a12a-37b9198b3ce7
1:2	Statistical	3	http://app.supramolecular.org/bindfit/view/0ef94382-dad6-4216-a012-0aacc7d57f40
2:1	Non-Coop	1	http://app.supramolecular.org/bindfit/view/865606e0-75ce-49d7-a050-f742b6848993
2:1	Non-Coop	2	http://app.supramolecular.org/bindfit/view/6764d64e-2e7b-472f-82ef-097272686ddd
2:1	Non-Coop	3	http://app.supramolecular.org/bindfit/view/3707bbce-4c0e-440a-b524-1c1168afb4cb

Table S9 The supramolecular.org (Bindfit) permanent URL links for the fitting results for each iteration and each binding model for the NMR titration of **Tf-SA4** (1 mM) and **2-DASPI** in 1:1 (v/v) H_2O:CD_3CN mixture at pH$_{app}$ 8.2 (sodium borate buffer 50 mM).

S6.4 Analysis for experiment at pH$_{app}$ 8.6

Model (C:D)	*n*	Flavour	K_1	K_2	cov_{fit}	BIC	ΔBIC
1:1	1		670.21		3.16E-03	-305.18	126.52
	2		675.58		2.73E-03	-309.41	78.95
	3		671.98		2.81E-03	-308.22	120.39
	Mean(SD)		*672.59 (2.74)*		*2.90E-03 (2.26E-04)*	*-307.60 (2.18)*	
1:2	1	Full	539.16	126.56	3.70E-05	-428.43	3.26
	2		659.81	125.61	1.49E-04	-386.10	2.26
	3		524.99	114.79	3.52E-05	-425.65	2.96
	Mean(SD)		574.65 (74.09)	*122.32 (6.54)*	*7.36E-05 (6.49E-05)*	*-413.39 (23.68)*	
1:2	1	Non-Coop	544.99	136.25	3.71E-05	-431.70	0.00
	2		713.41	178.35	1.57E-04	-388.36	0.00
	3		536.23	134.06	3.64E-05	-428.60	0.00
	Mean(SD)		*598.21 (99.86)*	*149.55 (24.97)*	*7.68E-05 (6.93E-05)*	*-416.22 (24.18)*	
1:2	1	Additive	458.37	-15.67			
	2		517.16	-11.68			
	3		457.12	-15.84			
	Mean(SD)		477.55 (34.31)	-14.40 (2.35)			
1:2	1	Statistical	3348.59	837.15	3.72E-03	-299.49	132.21
	2		3329.06	832.27	3.89E-03	-298.56	89.80
	3		3380.21	845.05	3.36E-03	-302.96	125.64
	Mean(SD)		*3352.62 (25.81)*	*838.16 (6.45)*	*3.66E-03 (2.73E-04)*	*-300.34 (2.32)*	
2:1	1	Full	229.19	-91.10			
	2		266.46	-90.28			
	3		270.28	-141.24			
	Mean(SD)		*255.31 (22.70)*	*-107.54 (29.19)*			
2:1	1	Non-Coop	1016.80	254.20	8.13E-04	-339.30	92.39
	2		999.61	249.90	6.33E-04	-347.10	41.26
	3		1109.92	277.48	7.09E-04	-343.52	85.08
	Mean(SD)		*1042.11 (59.35)*	*260.53 (14.84)*	*7.18E-04 (9.01E-05)*	*-343.31 (3.90)*	
2:1	1	Additive	892.60	-47.44			
	2		841.20	-36.78			
	3		933.07	-53.68			
	Mean(SD)		*888.96 (46.04)*	*-45.97 (8.55)*			
2:1	1	Statistical	411.10	102.78	3.71E-03	-299.95	131.75
	2		413.65	103.41	3.16E-03	-304.49	83.87
	3		411.30	102.83	3.41E-03	-301.91	126.69
	Mean(SD)		*412.38 (1.80)*	*103.00 (0.35)*	*3.42E-03 (2.74E-04)*	*-302.12 (2.28)*	

Table S10 Isotherms fitting results from supramolecular.org (Bindfit) for the NMR titration of **Tf-SA4** (1 mM) and **2-DASPI** in 1:1 (v/v) H_2O:CD_3CN mixture at pH$_{app}$ 8.6 (sodium borate buffer 50 mM). NF: no fitting possible. ΔBIC values were calculated from the lowest BIC value for each *n* (comparison for each sample).

Model	Flavour	*n*	Supramolecular.org (Bindfit) URL link
1:1		1	http://app.supramolecular.org/bindfit/view/7604e0e9-f67e-4fe6-a953-d68e3a3a3c69
1:1		2	http://app.supramolecular.org/bindfit/view/420b9a2a-6dfb-4d9b-ad1f-da9728ba34e6
1:1		3	http://app.supramolecular.org/bindfit/view/36627c70-e914-4aa0-a5dc-4abca570d27c
1:2	Full	1	http://app.supramolecular.org/bindfit/view/3fbb4cca-e5eb-4b72-b160-f1602d3e1ea4
1:2	Full	2	http://app.supramolecular.org/bindfit/view/bbafc3d7-1e84-4dab-9de2-6887c27272f4
1:2	Full	3	http://app.supramolecular.org/bindfit/view/ebcc4ef7-61e1-4d8f-baa4-0937092abb80
1:2	Non-Coop	1	http://app.supramolecular.org/bindfit/view/3c8b6143-73be-419f-91cb-3bb7ec2d1d0d
1:2	Non-Coop	2	http://app.supramolecular.org/bindfit/view/f331ce70-d671-4bd5-8d99-5e8d3bb36ac3
1:2	Non-Coop	3	http://app.supramolecular.org/bindfit/view/9e9df776-cd15-44d6-9a6d-d9010123442c
1:2	Statistical	1	http://app.supramolecular.org/bindfit/view/f189737f-d993-47f0-903b-462b6b523f54
1:2	Statistical	2	http://app.supramolecular.org/bindfit/view/d8f9d719-568e-46c3-9ea4-dd4891e024dc
1:2	Statistical	3	http://app.supramolecular.org/bindfit/view/067292e8-aba3-49d9-aead-508a3c562ab3
2:1	Non-Coop	1	http://app.supramolecular.org/bindfit/view/7c01f4fd-ea60-492b-8473-674d8fd2ae69
2:1	Non-Coop	2	http://app.supramolecular.org/bindfit/view/82053896-adb9-4f59-90a7-c68bb9a31793
2:1	Non-Coop	3	http://app.supramolecular.org/bindfit/view/a36ff888-603b-4b1e-bbaa-46cc12b4993c
2:1	Statistical	1	http://app.supramolecular.org/bindfit/view/a5fb0774-0aa9-411b-9ea6-c8413c425f36
2:1	Statistical	2	http://app.supramolecular.org/bindfit/view/8a40eb07-8ab4-4ef9-b9a9-c8c0678112b0
2:1	Statistical	3	http://app.supramolecular.org/bindfit/view/548ce881-1a72-41f3-9782-4a988435ec0e

Table S11 The supramolecular.org (Bindfit) permanent URL links for the fitting results for each iteration and each binding model for the NMR titration of **Tf-SA4 (**1 mM) and **2-DASPI** in 1:1 (v/v) H_2O:CD_3CN mixture at pH$_{app}$ 8.6 (sodium borate buffer 50 mM).

S6.5 Comparative analysis across studied pH$_{app}$ range

pH$_{app}$	cov_{fit} (avg (SD)) 1:1 model	1:2 Full model	1:2 Non-coop model	BIC (avg (SD)) 1:1 model	1:2 Full model	1:2 non-coop model
7.4	3.15E-04 (5.02E-05)	1.57E-05 (9.02E-06)	2.13E-05 (6.20E-06)	-390.04 (5.56)	-472.03 (18.70)	-463.77 (10.00)
7.8	8.08E-04 (8.96E-05)	4.51E-05 (3.81E-05)	4.78E-05 (3.82E-05)	-358.97 (3.27)	-441.33 (26.39)	-442.74 (26.54)
8.2	4.65E-03 (2.33E-04)	1.58E-04 (7.04E-05)	1.70E-04 (7.54E-05)	-276.00 (9.56)	-376.03 (1.27)	-367.26 (0.93)
8.6	2.90E-03 (2.26E-04)	7.36E-05 (6.49E-05)	7.68E-05 (6.93E-05)	-307.60 (2.18)	-413.39 (23.68)	-416.22 (24.18)

Table S12 Average covariance of the fit (cov_{fit}) and Bayesian Information Criterion (BIC) values for the NMR titrations at pH$_{app}$ 7.4, 7.8, 8.2, and 8.6, using different binding models: 1:1, 1:2 full, and 1:2 non-cooperative.

S7. nESI-MS spectra of mixture of Tf-SA4 with 2-DASPI at pH$_{app}$ 8.2

For mass spectrometry analysis, nanoelectrospray ionization (nESI) emitter tips were prepared by pulling single-barrel borosilicate capillaries (1.2 mm outer diameter, 0.69 mm inner diameter, Harvard Apparatus) using a P-97 Flaming/Brown Micropipette Puller (Sutter Instrument). The tips, with an approximate orifice size of 600 nm, were sputter-coated with a platinum layer and then loaded with 10 µL of the prepared sample solution (**Tf-SA4** 1 mM, **2-DASPI** 4 mM and ammonium acetate buffer 50 mM at pH$_{app}$ 8.2 in 1:1 (v/v) water:acetonitrile mixture). The data were acquired on an LTQ-Orbitrap-XL mass spectrometer (Thermo Scientific) operated in negative FTMS ion mode. Ionization was achieved using the nESI source with the following settings: source voltage of 1 kV, source temperature at 100 °C, capillary voltage of -23 V, and tube lens voltage of -38 V. Mass spectra were collected over m/z range of 200-2000 for a duration of 1 minute.

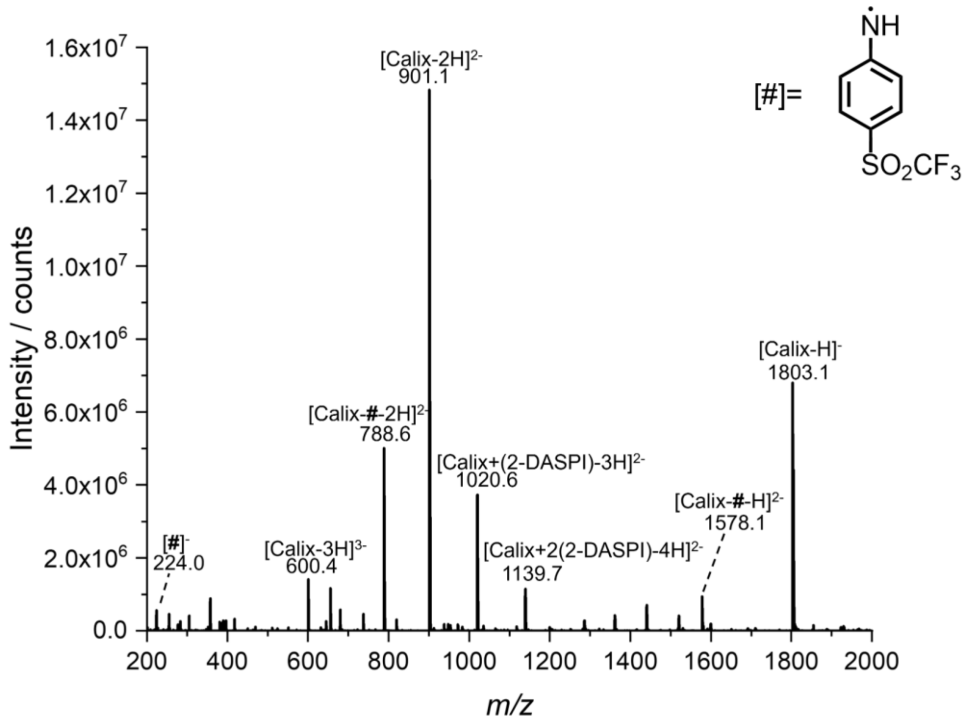

Figure S38 Whole nESI-MS spectrum of a mixture of Tf-SA4 (1 mM, "Calix" in figure), 2-DASPI (4 mM) and ammonium acetate buffer (50 mM) at pH$_{app}$ 8.2 in 1:1 (v/v) water:acetonitrile.

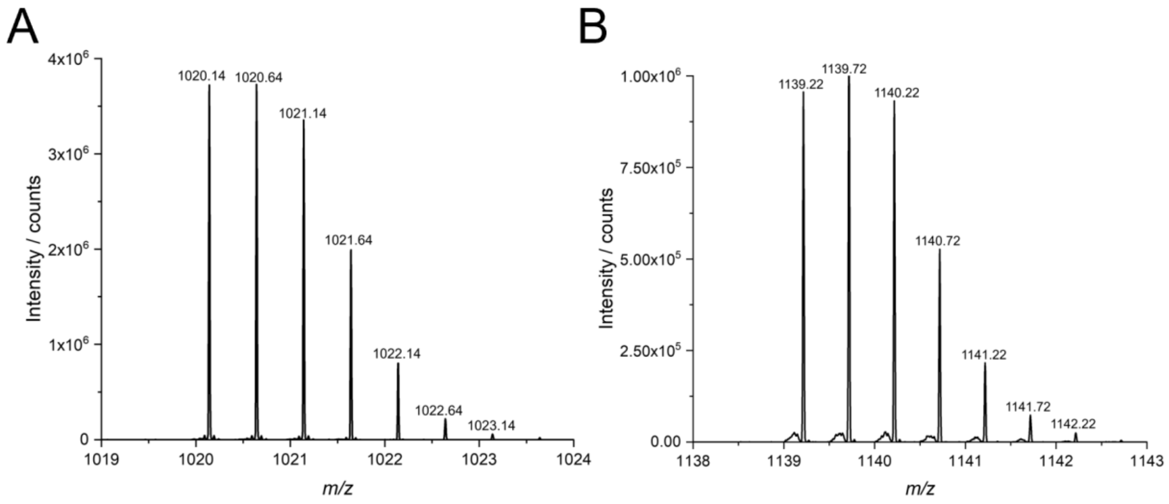

Figure S39 Zoomed nESI-MS spectrum showing isotopic pattern to reveal the doubly charged ion of **(A)** the 1:1 complex at *m/z* 1020 and **(B)** the 1:2 complex at *m/z* 1040. These ions can be assigned to $[\text{Calix+(2-DASPI)-3H}]^{2-}$ and $[\text{Calix+2(2-DASPI)-4H}]^{2-}$, where the charge state of the host is 3- and 4-, respectively.

S8. NOESY NMR experiment of Tf-SA4·(2-DASPI)$_2$ complex at pH$_{app}$ 8.2

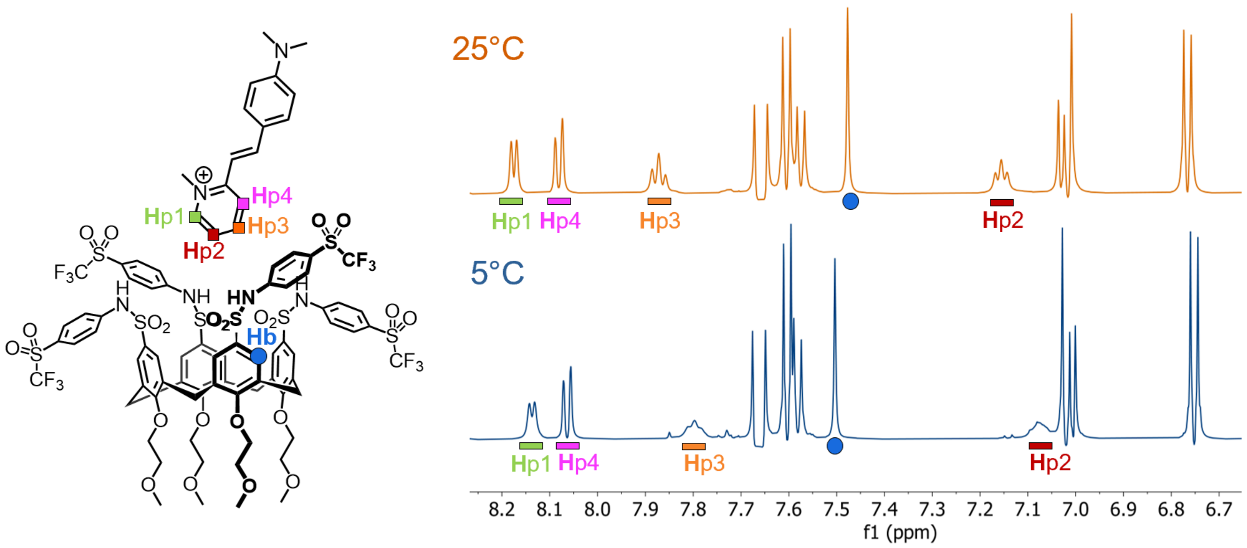

Figure S40 Partial ^{1}H NMR (600 MHz) spectra of **Tf-SA4** (1 mM) and **2-DASPI** (4.5 mM) at pH$_{app}$ 8.2 (sodium borate buffer 50 mM) in 1:1 (v/v) H_2O:CD_3CN, at 25 °C (top spectrum) and 5 °C (bottom spectrum). Selected protons were marked and it can be observed that the **2-DASPI** signals shifted upfield (Hp1 Δδ 0.0388 ppm, Hp4 Δδ 0.0175 ppm, Δδ Hp3 0.0744, Δδ Hp2 0.0776 ppm), as the binding constant increases at lower temperatures.

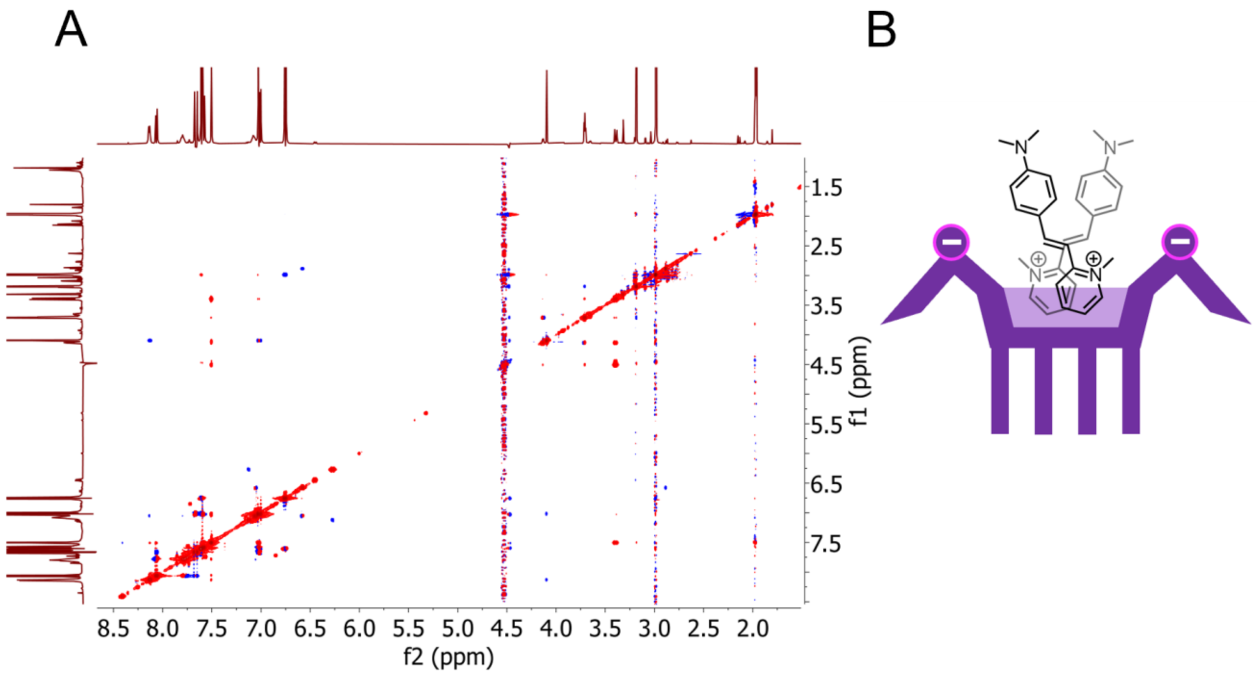

Figure S41 (A) NOESY NMR (600 MHz, 278 K) spectra of **Tf-SA4** (1 mM) and **2-DASPI** (4.5 mM) at pH_{app} 8.2 (sodium borate buffer 50 mM) in 1:1 (v/v) H_2O:CD_3CN, at 5 °C. **(B)** Proposed binding mode of the 1:2 complex of **Tf-SA4** and **2-DASPI**, based on the NMR titration data and NOESY experiment.

S9. Comparative analysis of pH responsiveness of WP6 and Tf-SA4

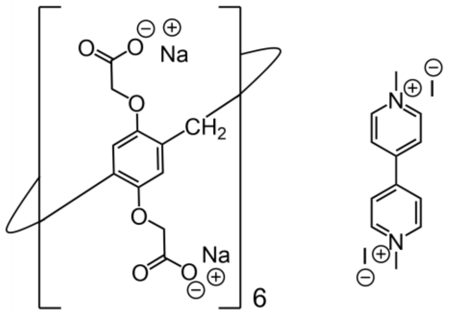

WP6 PQ

Figure S42 WP6 and paraquat (PQ) structures.

Host·Guest	pH range	Δ pH	$[H^+]_{min\ pH}$ / $[H^+]_{max\ pH}$	$K_{(max\ pH)}$ (M^{-1})	ΔG ($K_{(max\ pH)}$) (kJ/mol)	$K_{(min\ pH)}$ (M^{-1})	ΔG ($K_{(min\ pH)}$) (kJ/mol)	ΔΔG (kJ/mol)	(ΔΔG / ΔG ($K_{(max\ pH)}$)) ×100
WP6·PQ[37]	7.8 → 4.8	3.0	1000	$1.53 \times 10^{6\ b}$	–35.3	$1.64 \times 10^{5\ b}$	–29.7	5.6	16
Tf-SA4·(2-DASPI)$_2$	8.2 → 7.8 ^{a}	0.4	2.5	$7.4 \times 10^{2\ c}$	−16.4	$1.9 \times 10^{2\ c}$	−13.0	3.4	21

Table S13 Comparative analysis of pH responsiveness of WP6 and Tf-SA4. ^{a} Apparent pH of 1:1 (v/v) water:CD_3CN mixture. ^{b} 1:1 stoichiometry, determined by ITC. ^{c} 1:2 complex, microscopic binding constant was used ($K_m = \sqrt{\beta_{12}} = \sqrt{K_1 K_2}$).

S10. References

[38] C. D. Gutsche, "*p-tert*-butylcalix[4]arene" *Org. Synth.* **1990**, *68*, 234.

[39] C. D. Gutsche, L.-G. Lin, "The synthesis of functionalized calixarenes" *Tetrahedron* **1986**, *42*, 1633–1640.

[40] I. Bitter, A. Grün, B. Ágai, L. Tôke, "An easy access to tetra-o-alkylated calix[4]arenes of cone conformation" *Tetrahedron* **1995**, *51*, 7835–7840.

[41] D. Pedersen, C. Rosenbohm, "Dry Column Vacuum Chromatography" *Synthesis* **2004**, *2001*, s-2001-18722.

[42] M. Takahashi, N. Tsuji, K. Yazaki, Y. Sei, M. Obata, "A fluorescent calix[4]arene with naphthalene units at the upper rim exhibits long fluorescence emission lifetime without fluorescence quenching" *RSC Adv.* **2021**, *11*, 11651–11654.

[43] C. D. Gutsche, B. Dhawan, J. A. Levine, K. Hyun No, L. J. Bauer, "Calixarenes 9 : Conformational isomers of the ethers and esters of calix[4]arenes" *Tetrahedron* **1983**, *39*, 409–426.

[44] B. Genorio, T. He, A. Meden, S. Polanc, J. Jamnik, J. M. Tour, "Synthesis and Self-Assembly of Thio Derivatives of Calix[4]arene on Noble Metal Surfaces" *Langmuir* **2008**, *24*, 11523–11532.

[45] Bruker. APEX4, SAINT and SADABS, 2021. Bruker AXS Inc., Madison, Wisconsin, USA

[46] Sheldrick, G. M. SHELXT-Integrated Space-Group and Crystal Structure Determination., 2015.

[47] Sheldrick, G. M. SHELXL-Program for Crystal Structure Refinement, 2015.

[48] O. V. Dolomanov, L. J. Bourhis, R. J. Gildea, J. A. K. Howard, H. Puschmann, "OLEX2: a complete structure solution, refinement and analysis program" *Journal of Applied Crystallography* **2009**, *42*, 339–341.
